# miR-130a and Tgfβ Content in Extracellular Vesicles Derived from the Serum of Subjects at High Cardiovascular Risk Predicts their *In-Vivo* Angiogenic Potential

**DOI:** 10.1038/s41598-019-55783-7

**Published:** 2020-01-20

**Authors:** Claudia Cavallari, Federico Figliolini, Marta Tapparo, Massimo Cedrino, Alessandra Trevisan, Lorenza Positello, Pietro Rispoli, Anna Solini, Giuseppe Migliaretti, Giovanni Camussi, Maria Felice Brizzi

**Affiliations:** 1 2i3T Scarl, University of Turin, Turin, Italy; 2 Department of Medical Sciences, University of Turin, Turin, Italy; 3 Department of Surgical Sciences, University of Turin, Turin, Italy; 4 Department of Surgical, Medical, Molecular and Critical Area Pathology, University of Pisa, Pisa, Italy; 5 Department of Public Health and Pediatric Sciences, University of Turin, Turin, Italy

**Keywords:** Predictive markers, Metabolic disorders

## Abstract

Serum-derived extracellular vesicles (sEV) from healthy donors display *in-vivo* pro-angiogenic properties. To identify patients that may benefit from autologous sEV administration for pro-angiogenic purposes, sEV angiogenic capability has been evaluated in type 2 diabetic (T2DM) subjects (D), in obese individuals with (OD) and without (O) T2DM, and in subjects with ischemic disease (IC) (9 patients/group). sEV display different angiogenic properties in such cluster of individuals. miRNomic profile and TGFβ content in sEV were evaluated. We found that miR-130a and TGFβ content correlates with sEV *in-vitro* and *in-vivo* angiogenic properties, particularly in T2DM patients. Ingenuity Pathway Analysis (IPA) identified a number of genes as among the most significant miR-130a interactors. Gain-of-function experiments recognized homeoboxA5 (HOXA5) as a miR-130a specific target. Finally, ROC curve analyses revealed that sEV ineffectiveness could be predicted (Likelihood Ratio+ (LH+) = 3.3 IC 95% from 2.6 to 3.9) by comparing miR-130a and TGFβ content ‘in Series’. We demonstrate that sEV from high cardiovascular risk patients have different angiogenic properties and that miR-130a and TGFβ sEV content predicts ‘true ineffective sEVs’. These results provide the rationale for the use of these assays to identify patients that may benefit from autologous sEV administration to boost the angiogenetic process.

## Introduction

Cardiovascular complications are relevant causes of morbidity and mortality in patients with diabetes and obesity^1^. Recent evidence suggests that extracellular vesicles (EVs) may act as mediators of many pathophysiological processes^2–4^. EVs are small vesicles that are released from different cell types under normal and pathological conditions^5,6^. Increased levels of circulating EVs have been associated with vascular impairment and hypercoagulability, particularly in patients with diabetes and acute coronary syndrome, suggesting that they play a role in driving cardiovascular diseases^7,8^. Moreover, increased levels of circulating EVs, mainly from platelets and endothelial cells, has been proposed as a hallmark of cell dysfunction^9^. It has been extensively reported that EVs act as biological active vectors, and participate in the exchange of information between circulating and resident cells, including endothelial cells^2,10^. It has also been proposed that platelet-derived EVs play a role in the pathogenesis of atherosclerosis^11^.

EVs act as biological intermediaries, and mainly do so by delivering proteins, active lipids and extracellular RNAs^12^. However, the most frequently studied EV-mediated biological processes rely on microRNA (miRNA) transfer. miRNAs are a class of small noncoding RNAs post-transcriptionally regulating gene expression^13^. miRNAs are present in serum/plasma and it has been proposed that their distinctive expression patterns serve as disease fingerprints in many clinical settings^14^. Moreover, it has been shown that activated platelets can transfer functional miRNAs into vascular cells using EVs^15^. Similarly, EVs released from cytokine-induced inflamed ECs impact on both ECs and monocytes, and regulate the vascular inflammatory response via the expression of the intercellular adhesion molecule 1, ICAM-1, and the recruitment of inflammatory cells^16^. Indeed, changes in circulating EV cargo have been associated with endothelial^17^ and smooth muscle cell dysfunction in type 2 diabetes (T2DM)^18^.

There is a growing body of evidence to suggest that EVs can act as potential therapeutic, diagnostic and prognostic tools^19,20^. We have previously developed an *in-vitro* test of potency to predict healthy subject-derived serum-EV (sEV) pro-angiogenic properties^21^. Since angiogenesis, particularly in the heart, peripheral arteries, and kidney is impaired in patients with a high cardiovascular risk profile^22,23^, the aims of the present study are: (*i*) to investigate *in-vitro* and *in-vivo* sEV proangiogenic capability by assessing endothelial cell proliferation/vessel-like structure formation *in-vitro* and neoangiogenesis in male severe combined immunodeficiency (SCID) mice; (ii) to explore the possibility that a specific sEV cargo could mark effective and ineffective sEV in these high-risk patients making relevant their more safe and feasible autologous clinical application. Additionally, particular attention has been devoted to the analysis of sEV TGFβ content as a pre-selected target, and miRNAs by miRNome profiling.

## Results

### sEV characterisation

**Figure 1:**
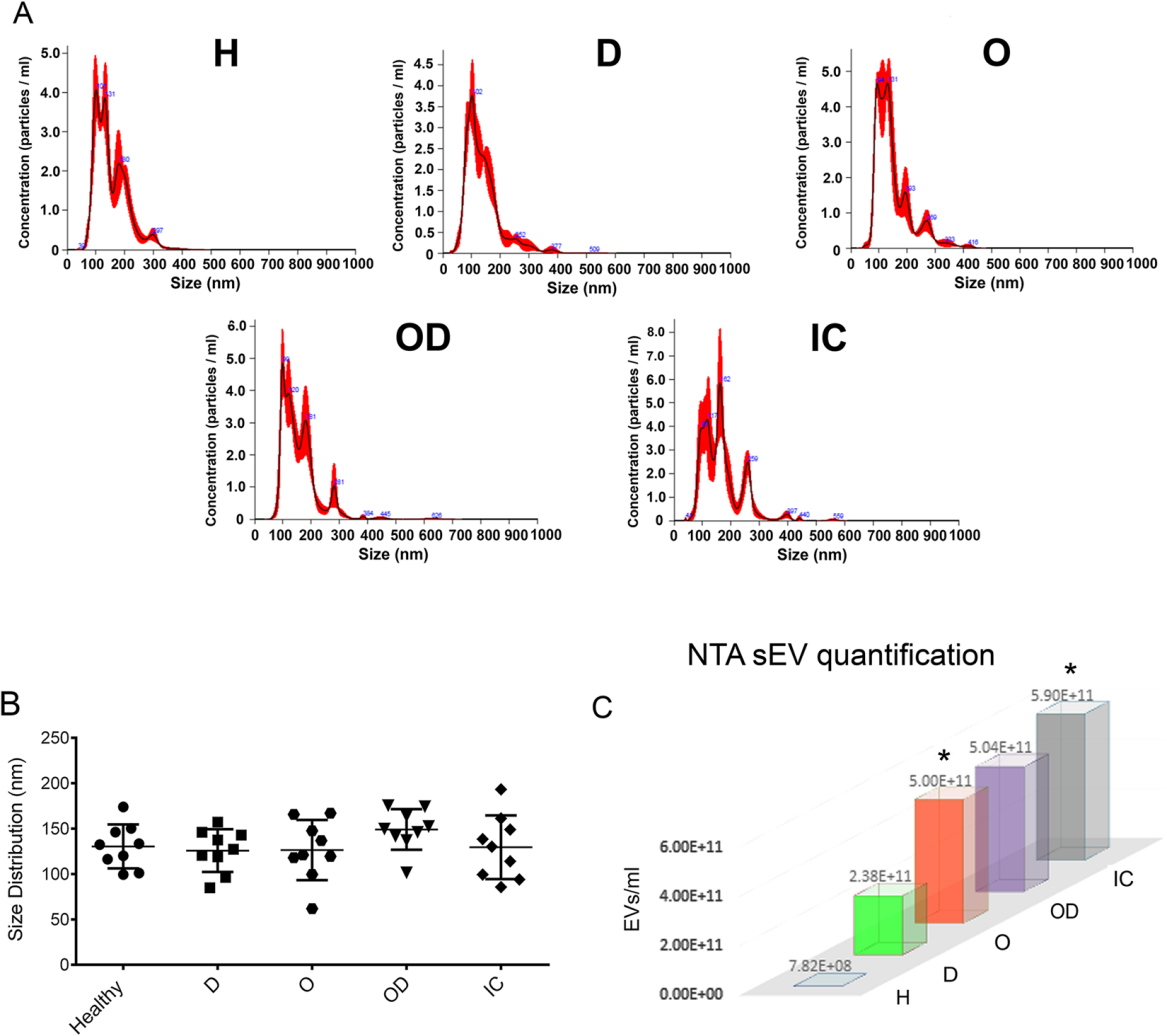
Nanosight sEV characterisation. (**A**) Representative images of NTA analysis referred to each group of patients. **(B)** Dot-plot graph representing NTA size distribution with mean size values for healthy, obese, T2DM, obese/T2DM and ischemic subjects. **(C)** The histogram reports the number of EVs recovered from the serum of each group of patients. *p < 0.05 obese and ischemic patients vs healthy subjects (One-way ANOVA followed by Multiple Comparison Test). (n = 9 patients/group). H = Healthy subjects; D = Diabetic patients; O = Obese patients; OD = Obese/Diabetic patients; IC = Ischemic patients.

9 sEV samples derived from healthy individuals (H) and 36 derived from patients were examined for their number and size. Patient clinical data are reported in Table 1. The size distributions of sEV from healthy individuals and patients did not show any significant differences (Fig. 1A,B). The average particle size was around 138 nm. A significantly higher sEV concentrations were detected in obese (O) and ischemic patients (IC) (Fig. 1C). The Nanoparticle Tracking Analysis (NTA) quantification values for each subject are reported in Supplementary Table S1. Characterisation by Guava FACS analyses revealed that sEV expressed CD144 and CD42b, as is typical of endothelial and platelet markers, respectively. VEGFR3+ sEVs were also found (Fig. S1).

**Table 1:** Healthy, Diabetic, Obese, Diabetic/Obese, and Ischemic patients’ clinical data.

	H	D	O	OD	IC
Age	58.0 ± 3.9	58.9 ± 6.5	49.7 ± 11.0	53.2 ± 4.1	67.2 ± 4.7
F/M	4/5	4/5	4/5	4/5	4/5
BMI	25.1 ± 0.9	25.6 ± 1.3	48.1 ± 4.9	40.9 ± 2.4	29.3 ± 6.1
HbA1c%	4.5 ± 0.2	7.9 ± 1.0	4.9 ± 0.5	7.2 ± 0.7	6.3 ± 1.7
Total Col.	163.2 ± 11.7	169.3 ± 34.5	182.3 ± 62.1	172.0 ± 44.3	123.0 ± 20.1
Smokers	5	6	6	6	5

F/M = female/male; BMI = Body Mass Index; Total Col = Total cholesterol (mg/dL); HbA1c = % Glycated Hemoglobin. H = Healthy; D = Diabetic patients; O = Obese patients; OD = Obese/Diabetic patients; IC = Ischemic patients. The IC group includes: patients with diabetes (IC26, IC28), obesity (IC24), and both diseases (IC25, IC27, IC30).

### Patient derived sEV pro-angiogenic activity

**Figure 2:**
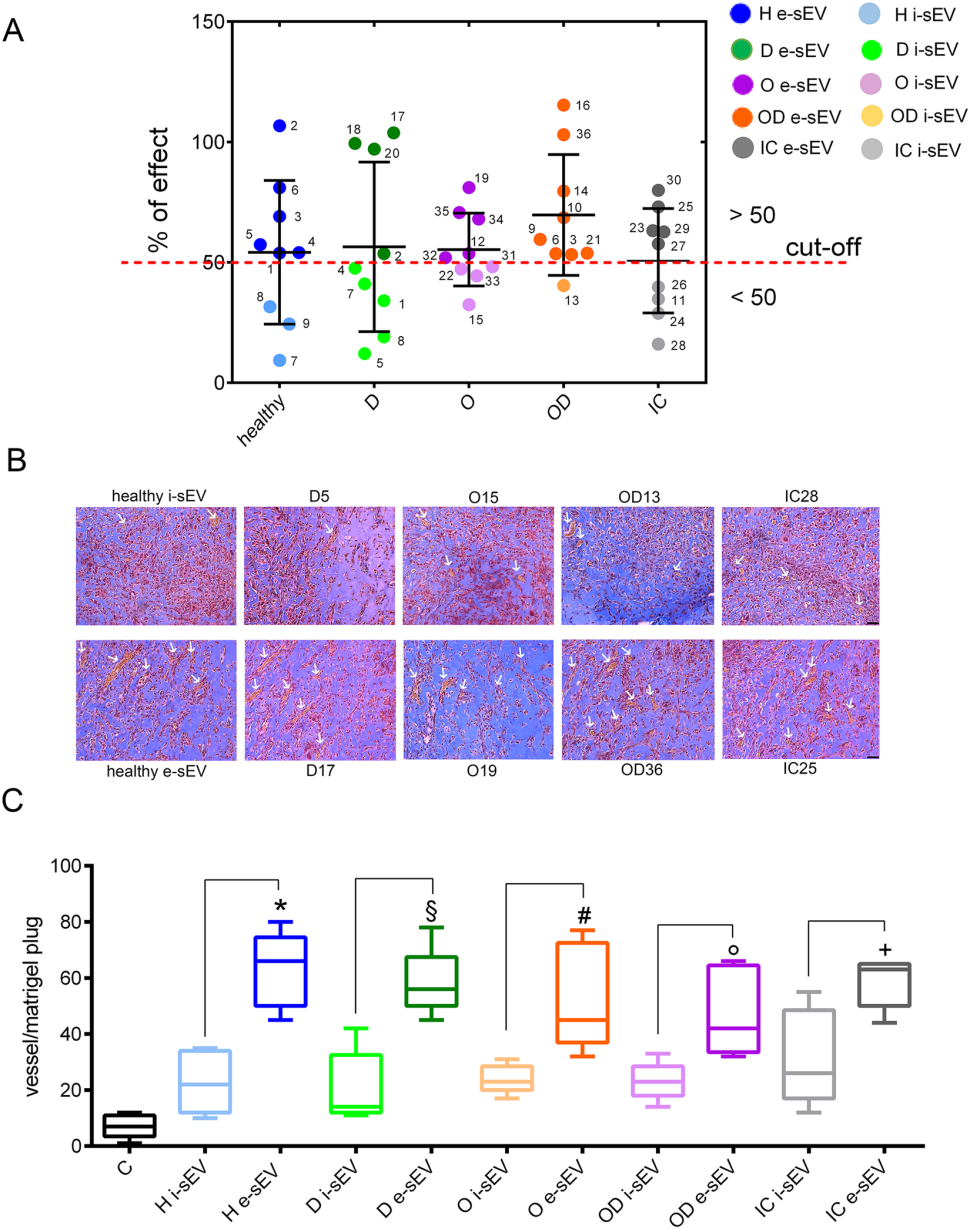
*In-vitro* and *in-vivo* angiogenesis in response to sEVs. (**A**) Dot plot graph reporting the *in-vitro* proangiogenic activity of sEV recovered from each patient. The number corresponds to each patient per group (see Supplementary Table 3). The dotted line defines the cut-off for effective and ineffective sEV. The light colour corresponds to ineffective sEV per each group. **(B)** Representative images of vessels formed in response to effective and ineffective sEV. The number refers to patient sEV. (n = 3 each group except for OD. The same sample was used in 3 independent experiments). **(C)** *In-vivo* quantitative analysis of vessels counted in 10 sections of Matrigel for each experimental condition. Data represent the mean value of untreated (**C**) (n = 3) and treated mice with: healthy ineffective sEV (i-sEV), healthy effective sEV (e-sEV); T2DM ineffective sEV (D i-sEV), T2DM effective sEV (D e-sEV); obese ineffective sEV (O i-sEV), obese effective sEV (O e-sEV); obese/T2DM ineffective sEV (OD i-sEV), obese/T2DM effective sEV (OD e-sEV); ischemic ineffective sEV (IC i-sEV), ischemic effective sEV (IC e-sEV). *p < 0.05 healthy e-sEV vs. healthy i-sEV; ^§^p < 0.05 T2DMe-sEV vs. T2DM i-sEV; ^#^p < 0.05 obese e-sEV vs. obese i-sEV; °p < 0.05 obese/T2DM e-sEV vs. obese/T2DM i-sEV; ^+^p < 0.05 ischemic e-sEV vs. ischemic i-sEV ischemic; (One-way ANOVA followed by Multiple Comparison Test). (n = 3 each group except for OD. The same sample was used in 3 independent experiments) ECs (red), erythrocytes (yellow) and Matrigel (light blue) staining in Matrigel plugs. (Original magnification: x200; scale bar: 12 µm).

sEV from the different patient groups were evaluated for their angiogenic potential *in-vitro* according to our angiogenesis potency test^21^. As shown in Supplementary Table S2, we were able to distinguish effective and inefficient sEV in all patient groups. Unlike in healthy subjects, almost all obese/T2DM (OD) patients are characterised by the presence of sEV with proangiogenic capability. Individually detailed results are shown in Fig. 2A. An *in-vivo* angiogenic assay was performed using effective and ineffective sEV from the different patient groups to validate our *in-vitro* data in patients. As shown in Fig. 2B,C, we were able to demonstrate the pro-angiogenic capability of patient-derived sEV *in-vivo*. No statistical differences in the expression of CD144, CD42b and VEGFR3 were found when sEV with or without angiogenic potential from both healthy subjects and patients were compared.

### TGFβ EV content and sEV angiogenic potential

**Figure 3:**
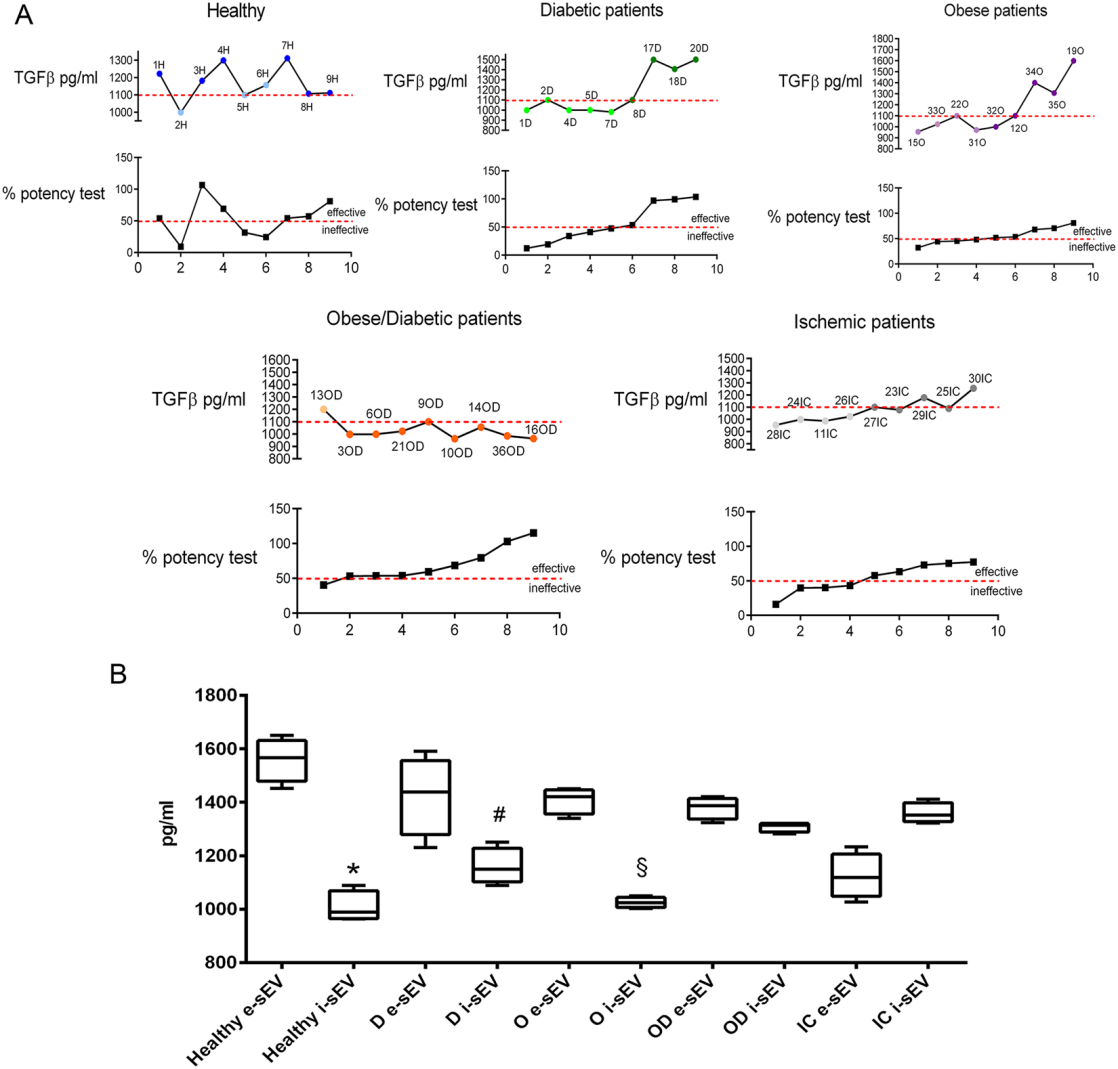
TGFβ sEV content. (**A**) Data obtained per patient/group are reported. The upper curve refers to sEV TGFβ content, while the lower to the % of the test of potency. The dotted line defines the cut-off for effective and ineffective sEV. The number corresponds to each patient (n = 3 each group, except for OD. The same sample was used in 3 independent experiments). (**B**) Min to max columns represent TGFβ content in effective and ineffective sEVs from each group. *p < 0.05 healthy e-sEV vs. healthy i-sEV; ^#^p < 0.05 T2DM e-sEV vs. T2DM i-sEV; ^§^p < 0.05 obese e-sEV vs. obese i-sEV; (One-way ANOVA followed by Multiple Comparison Test). (n = 3, except for OD. The same sample was used in 3 independent experiments).

We have previously shown that TGFβ is crucial for sEV-mediated angiogenic effects in healthy subjects^21^. ELISA was therefore performed to analyse the TGFβ content in sEV from our patients and results were compared to those of healthy subjects. As shown in Fig. 3A, TGFβ sEV content in obese and T2DM patients was significantly associated with their angiogenic activity. No significant differences in TGFβ content were detected in OD patients (Fig. 3A,B), in accordance with their functional activity. Similar results were found in IC patients. However, the TGFβ sEV content in the IC-patient group correlated with their angiogenic properties (Fig. 3B, Supplementary Table S3), suggesting that, unlike in OD patients, TGFβ cargo could be a relevant driver of their angiogenic potential. Accordingly, sEV from patient OD13 were enriched in TGFβ (Fig. 3A) even though they were ineffective.

### sEV miRNome profile and target validation

**Figure 4:**
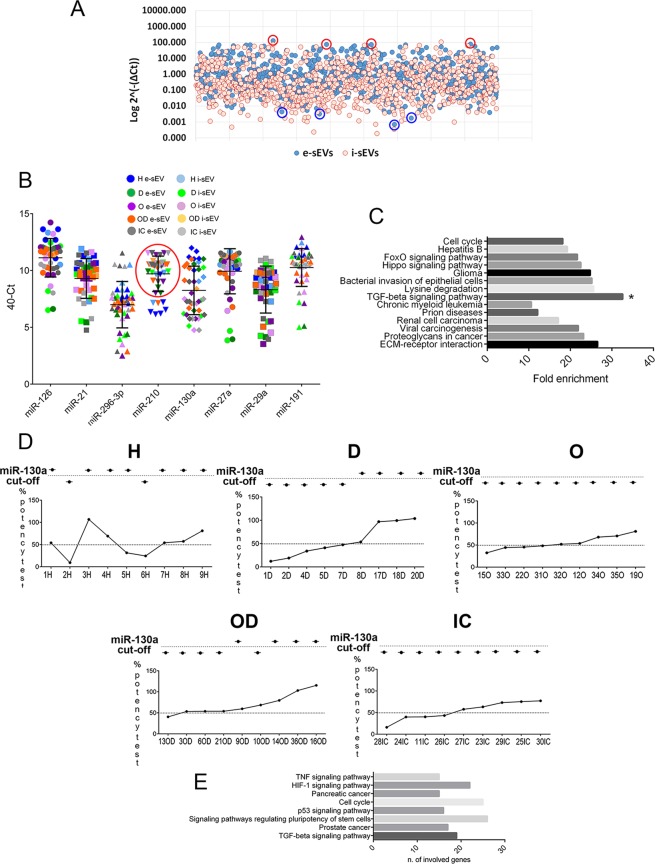
miR expression and pattern analysis of sEV. (**A**) miRNome miRNA qPCR profiler array analysis on healthy effective (e-sEV) and ineffective (i-sEV). miRNAs were detected by RT-PCR. Results are reported as Log2^−(∆Ct)^ (Normalized Relative Quantities) values. Dark blues circles correspond to e-sEV, while light red corresponds to i-sEV (n = 3/each). In red and blue, miRNAs upregulated and down regulated, respectively (t-test, p value < 0.05, see Supplementary Table 5). (**B**) miRNA validation in patient sEV. RT-PCR for miR-126, miR-21, miR-296-3p, miR-210, miR-130a, miR-27a, miR-29a and miR-191 was performed in all patients and healthy donors. Results are reported as 40-Ct. The red circle indicates patients with high miR-210 expression. (**C**) Gene Ontology analysis. Network analysis of pathways positively correlated with the miRNAs indicated above. Data were obtained from DIANA-mirPath analyses. The significant enrichment of the TGFβ-associated pathway was identified. (**D**) miR-130a distribution, patient-by-patient, including healthy subjects is reported and compared with the % of the test of potency. (**E**) Network analysis of pathways positively correlated with miR-130a. Data were obtained from DIANA-mirPath analysis. Only pathways including at least 15 genes were selected.

The role of miRNAs in angiogenic processes has been extensively documented^24,25^. The possibility that differential miRNA content in sEV could also contribute to their functional capability was therefore investigated. A miRNome analysis (Fig. 4A and Supplementary Table S4), performed using healthy effective and ineffective sEV (3 samples/each), led to the identification of 8 angio-miRNAs as the most differentially expressed: miR-126, miR-21, miR-296-3p, miR-210, miR-130a, miR-27a, miR-29a and miR-191 (Fig. 4B and Supplementary Table S5). miR-126, miR-130a, miR-27a and miR-296-3p were up-regulated, while miR-21, miR-29a, miR-191 and miR-210 were down-regulated in sEV with proangiogenic capability (Fig. 4A). The expression level of differentially expressed miRNAs were evaluated in all patients and healthy subjects and reported in Fig. 4B. This miRNA-list was submitted to DIANA-mirPath analysis. As shown in Fig. 4C, the TGFβ signalling pathway (p-value: 9.11E-08) was found to be significantly enriched. To investigate whether this difference was also associated with patient-sEV functional activity, their expression, evaluated using real-time PCR (cut-off Ct value 30), was compared to sEV angiogenic potential. We found an enrichment of miR-210 in almost patient-derived sEV (Fig. 4B), in accordance with results from Shalaby *et al*.^26^. However, no significant correlation with biological activity was detected. Interestingly, we were able to demonstrate that, unlike miR-210, miR-130a showed the highest level of correlation between Ct distribution and the angiogenesis potency test (3 out of 9 in O patients, 5 out of 9 in OD and 4 out of 9 in IC patients), and in all T2DM (D) patients (Fig. 4D and Table 2). These results indicate that in the D group both TGFβ and miR-130a sEV content strongly correlated with their angiogenic properties (Figs. 3A and 4D).

**Table 2:** miR-130a and TGFβ ‘in series’ combination test.

Parameters		95% Conf. Int.	
		Inf	Sup
Se	0.824	0.59	0.94
Sp	0.750	0.57	0.87
ACC	0.778	0.64	0.87
PPV	0.667	0.45	0.83
NPV	0.875	0.69	0.96
LH+	3294118	2.62	3.97
LH−	0.235294	−0.81	1.28

List of values obtained by combining the two measures ‘in series’ (taking as ‘ineffective’ those sEV defined as ‘ineffective’ in both measures). Good levels of sensitivity and increased specificity values were detected (Se = 0.82; Sp = 0.75). Se = Sensitivity; Sp = Specificity; ACC = Accuracy; PPV = Positive Predictive Value; NPV = Negative Predictive Value; LH = Likelihood Ratio.

**Figure 5:**
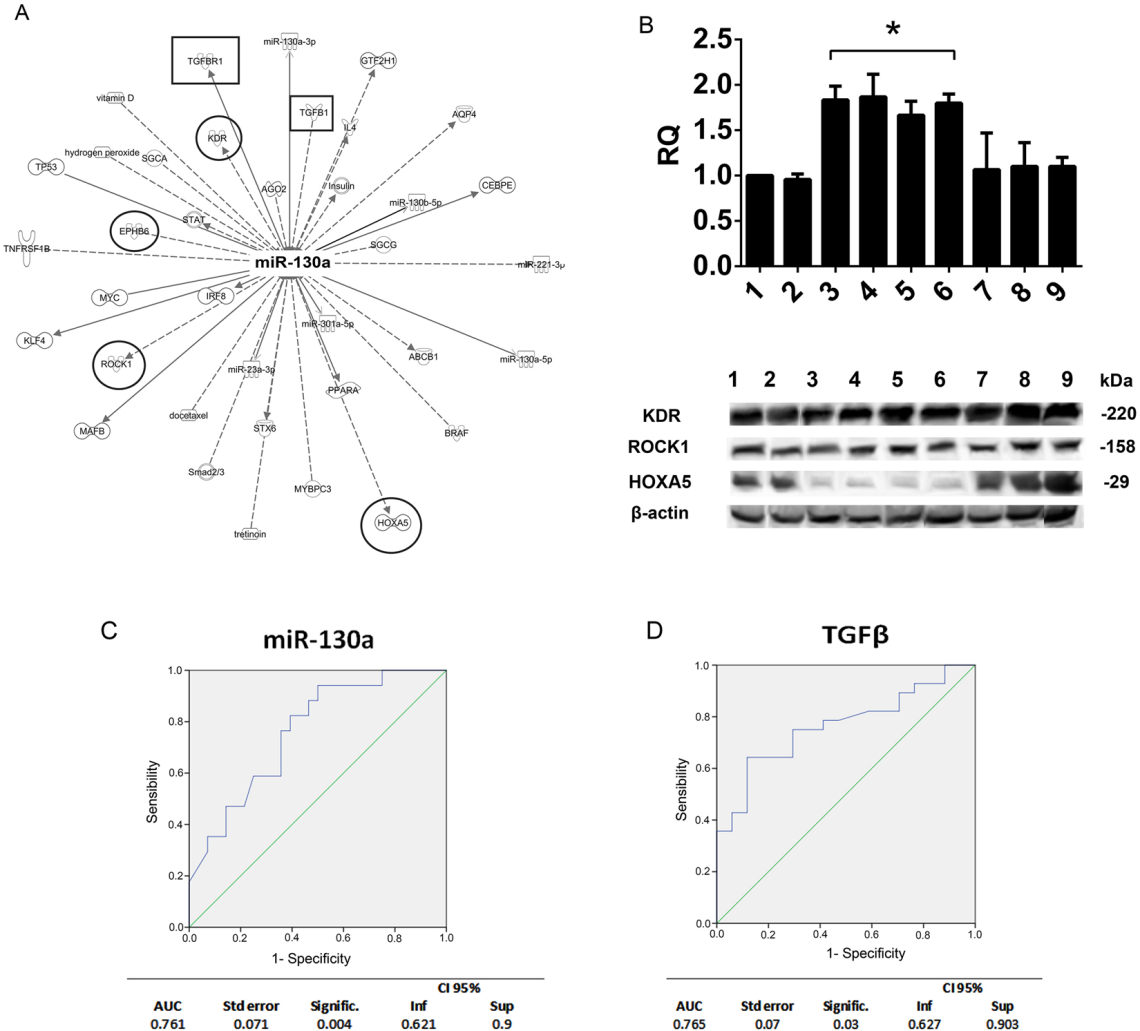
miR-130a integrated interaction networks, target validation and ROC analysis. (**A**) Network analysis between miR-130a and mRNA targets. Lines represent interactions between genes and miR-130a predicted using the IPA Software; indirect interactions (dotted lines), direct interactions (continuous lines). Squares include TGFβ and TGFBR. Circles include genes involved in angiogenesis (KDR, EPHB6, ROCK1, HOXA5). (**B**) Validation of miR-130a target(s). Upper panel RT-PCR relative quantification (RQ): miR-130a relative amount in untreated ECs (1), in ECs transfected with miRNA Scramble (Scr) (2), with mimic-miR-130a (3), or treated with sEV from patients D17 (4), D18 (5), D20 (6), D1 (7), D2 (8), and D4 (9) is reported. (*p < 0.05 samples 3, 4, 5, 6 vs. samples 1, 2, 7, 8, 9). Lower panel: KDR, ROCK1, HOXA5 and β-actin expression was evaluated in the above samples. sEV from patients D17, D18, D20 are enriched in miR-130a, while sEV from D1, D2 and D4 carry low miR-130a level. (**C**) ROC analysis. miR-130a and TGFβ sEV content from all patients and healthy subjects were analysed. Predictive capacity was evaluated for each of the two measures individually. A table reporting the AUC values, standard errors, p-values and threshold values is also reported.

DIANA-mirPath analyses were interrogated, using miR-130a, by selecting pathways involving at least 15 genes. Once again, a significant enrichment in genes involved in the TGFβ pathway, among others, was detected (Fig. 4E). The network predicted by IPA for miR-130a target genes identified several genes (including: *KDR*, *HOXA5*, *ROCK1*, *EPHB6*) that are clearly related to the angiogenic process (Fig. 5A)^27–30^. Moreover, TGFβ and TGFBR1 genes were found among the miR-130a interactors (Fig. 5A). These results support the miR-130a contribution to the sEV-mediated mechanisms of action. To validate predicted miR-130a target(s), ECs were either transfected with mimic-miR-130a or stimulated with sEV enriched in miR-130a and evaluated by RT-PCR and Western blot. Scramble miRNA and sEV carrying low miR-130a served as controls. Cell miR-130a enrichment was demonstrated by RT-PCR (Fig. 5B upper panel). The results of protein expression reported in Fig. 5B (lower panel) show that, among the predicted miR-130a targets, only homeoboxA5 (*HOXA5*) was downregulated in ECs enriched in miR-130a. These results are consistent with the observation that suppression of *HOXA5* attenuates the angiogenic process^30^.

### miR-130a and TGFβ sEV content are valuable predictive markers for the identification of ‘true’ ineffective sEVs

ROC curves were interrogated to identify a simple method to discriminate sEV functional capability in all patient’s groups, as reported by C. Gai *et al*.^31^. In detail, ROC analyses assessed whether miR-130a and TGFβ sEV content have the predictive capacity to identify sEV that display proangiogenic capability from potency test findings. The results demonstrated that both miR-130a and TGFβ can be considered good predictive measures of the ‘true ineffective sEV’ that were identified by RS, with statistically significant AUC values (Fig. 5B,C). Both measures displayed good sensitivity in identifying as ‘ineffective’ the ‘true ineffective sEV’ identified by RS. This was particularly evident and further underlined by the LH+ = 1.88 (IC95% from 1.49 to 2.27), for miR-130a (Se = 0.94 IC95% from 0.73 to 0.99) and for TGFβ (Se = 0.88 IC95% from 0.66 to 0.97). However, both measurements had a low specificity value (miR-130a: Sp = 0.50; TGFβ Sp = 0.64).

A good sensitivity level and increased specificity were detected (Sp = 0.75; Se = 0.82) by combining the two measures ‘in series’ (taking as ‘ineffective’ those sEV defined as ‘ineffective’ in both measures). The LH + value (Table 2) further supports these results.

## Discussion

The present study demonstrates that a varied cluster of chronic diseases that are characterised by high cardiovascular risk are associated with different sEV angiogenic properties. We also provide evidence to show that, as in healthy subjects, the high TGFβ content of sEV in T2DM and obese patients predicts their proangiogenic capability (Fig. 3A). Moreover, miRnomic analyses demonstrated that miR-130a is the most relevant sEV miRNA, as it can predict their angiogenic properties. This is particularly true in T2DM patients (Fig. 5D). Finally, the ROC curve that combines data from miR-130a and TGFβ sEV content ‘in series’ demonstrated that this easy and simple assay is able to predict ‘true ineffective sEV.

The increased risk of cardiovascular events is a common feature in T2DM and obese patients^32^. Impaired vessel formation is still considered a relevant mechanism in abnormal vascular remodelling in these clinical settings^33^. Moreover, (micro)vessel dysfunction and vessel regression/rarefaction combined with poor neovascularization contribute to pathophysiology of systemic metabolic syndrome/diabetic heart failure^34^. Therefore, boosting the neovascularisation of damaged tissues remains necessary for the improvement of patient outcomes.

Several therapeutic approaches to improve vascular remodelling in patients at high cardiovascular risk have been proposed^35^. In particular, angiogenic growth factors, and cell-based therapy exploiting bone marrow, peripheral blood, mesenchymal, and adipose tissues as stem/progenitor cell sources have been evaluated in clinical trials to improve long-term patient outcomes^36^. Unfortunately, they have failed to provide real benefits^37^, meaning that novel treatment options are still required. In this paper, the angiogenic properties of sEV collected from patients at high risk of cardiovascular events have been thoroughly investigated, as has the role of their cargo in promoting vessel formation.

EVs are released by many cell types and exert their biological effects via the transfer of mRNAs, proteins, lipids and a wide range of non-coding RNA, such as miRNAs^38^. EVs, and in particular their composite cargos, have been consistently demonstrated to contribute to the development of cardiovascular diseases^39^. However, the therapeutic delivery of stem cell- and serum-derived EVs have been proposed as cell-free approaches for cardioprotection in models of cardiac ischemic injury^8,20^. We have recently demonstrated that the sera of healthy donors could contain pro-angiogenic EVs, which principally express endothelial and platelet markers. Moreover, we found that, when effective in promoting vessel formation *in-vivo*, healthy subjects-derived sEV also prevent skeletal-muscle damage in animals with lower limb ischemia^21^. This indicates that sEV angiogenic properties can be simply provided by the *in-vivo* angiogenic assay. Since angiogenesis is dysfunctional in obese, obese/T2DM, IC, and more importantly in T2DM patients, sEV angiogenic capability was investigated as part of the search for novel therapeutic options. We first noticed that patients with higher cardiovascular risk have a higher number of circulating sEV than healthy subjects. Endothelial and platelet antigens were expressed on these sEV. VEGFR3-expressing sEV were also found, indicating that EVs from lymphatic vessels can be also detected in circulation. VEGFR3 is expressed by lymphatic vessels and drives lymphangiogenesis by binding to VEGF-C^40^. The impairment of VEGF-C–induced lymphangiogenesis via the epsin-mediated degradation of VEGFR3 has recently been reported in T2DM^41^. No differences in the number of VEGFR3 + sEV were detected. The possibility that the release of VEGFR3 + EVs may be a protective mechanism against VEGFR3 degradation can therefore be postulated.

The role of sEV in driving vascular complications in diabetes and obesity have been extensively documented^42,43^. Moreover, increases in the numbers of circulating EVs^44–46^ that display platelet^47^, monocyte^48^ and endothelium markers^49^ have been reported in individuals with cardiovascular risk factors. Fittingly, we have demonstrated that IC and obese individuals showed a significantly increased number of sEV. Increases in the number of circulating EVs that display adipose tissue markers have also been reported^50^. Although our data are in line with the study by Murakami *et al*.^51^, the possibility that adipose tissue-derived EVs may be a relevant source of sEV in obese patients cannot be ignored.

The role of sEV in boosting the activation of the coagulation cascade has been proposed as an explanation for the increased risk of cardiovascular events in obese and T2DM patients^52^. By contrast, whether and how sEV, obtained from sera of patients with cardiovascular-risk factors, display proangiogenic properties is still matter of study. The analysis of patient-derived sEV showed that obese/T2DM subjects had a much more active sEV population than healthy donors, while T2DM had the lowest activity. These data were also confirmed by the IC group that includes patients with diabetes (IC26, IC28), obesity (IC24), and both diseases (IC25, IC27, IC30). The pro-angiogenic potential of patient-derived sEV that was validated *in-vivo* further supported the *in-vitro* results. No significant correlation between sEV number and angiogenic activity was detected in patient’s groups. This was particularly true in IC patients (see Figs. 1C and 3C). This suggests that sEV angiogenic properties may depend on their specific cargo rather than their absolute number. The possibility that sEV from OD patients are much more enriched with pro-angiogenic factors should be considered.

The role of TGFβ during embryogenesis and in adult-vessel homeostasis has been highlighted by gene inactivation^53^. We have previously shown that sEV TGFβ content was a relevant mediator of sEV proangiogenic activity^21^. We wondered whether sEV TGFβ content could also account for the angiogenic potential of patient-derived sEV. The ELISA assay demonstrated that sEV TGFβ content correlated with their angiogenic potential in only two patient groups: T2DM and obese. This relationship was not significant in the other two subject groups, possibly because of the small number of recruited patients. In all patient group, TGFβ sEV content correlated with the proangiogenic activity when it was compared to sEV angiogenic capability patient-by-patient except for the obese/T2DM group. Therefore, the enrichment of proangiogenic mediators, which are different from TGFβ, can be proposed to explain this difference. Further studies are required to validate this possibility.

However, the pro-fibrotic properties of TGFβ should not be neglected. Indeed, besides acting on ECs to induce the angiogenic switch, TGFβ is a crucial mediator of fibrosis in different organs^54,55^. This would be relevant in all patient groups analysed in this study, since additional kidney, liver, and heart scarring and thickening would eventually lead to permanent organ failure. Therefore, being such effect particularly relevant for sEV clinical application, additional studies should be performed to investigate sEV pro-fibrotic effect in the heart, kidney, and liver before exploiting sEV to improve angiogenesis in patients at high cardiovascular risk.

Besides EV protein cargo, EV miRNA content has been reported to impact in their mechanism of action^56^. Indeed, differentially expressed angiogenic miRNAs were detected in sEVs from healthy donors that display different biological responses (with or without angiogenic potential). Interestingly, DIANA-mirPath analyses that included the indicated miRNAs (miR-126, miR-130a, miR-27a, miR-296-3p, miR-21, miR-29a, miR-191 and miR-210) demonstrated a significant enrichment in genes that are involved in the TGFβ-signaling pathways. High miR-210 levels have been proposed as a novel diagnostic biomarker for patients with cardiovascular complications^26^. Our finding that sEV from almost all patients (Fig. 5B) were enriched in miR-210 sustains the possibility that it can also be considered a biomarker for increased cardiovascular risk. This is consistent with the finding that an extensive spectrum of miR-210 targets that mainly regulate mitochondria metabolism, angiogenesis, DNA repair and cell survival have been reported^49^. Interestingly, we noticed that miR-130a content correlated with sEV angiogenic properties in all T2DM patients and in diabetic IC patients (IC26, IC28). Several genes involved in the angiogenic process, such as *KDR*, *HOXA5*, *ROCK1* and *EPHB6*^27–30^, were identified among the most relevant miR-130a interactors by IPA. Indeed, miR-130a overexpression or EC treatment with sEV enriched in miR-130a led to *HOXA5* downregulation. This may be a proof of concept for its contribution to the angiogenic process. Moreover, the finding that TGFβ and TGFBR1 were also found among the genes that are under the control of miR-130a further confirms the cooperation between miR-130a and TGFβ in driving the proangiogenic activity of biologically active sEV.

In general, EVs are considered main paracrine and endocrine players, and their application as therapeutic agents would allow a safe regenerative approach. We focused on the angiogenic switch as a showcase model for the characterisation, efficacy testing and potential clinical translation of sEV as therapeutics. We provide evidence that sEV package can theoretically act on different tissues requiring new vascularization via TGFβ and post-transcriptional regulation of gene involved in new vessel formation. However, the therapeutic effects of the off-the-shelf sEV “secretome” largely depend on the complex interaction of all miRNAs and proteins taken together. This implies that, besides TGFβ and/or miR-130a, other proteins or genetic information transferred by sEV may drive a diverse range of biological processes in different target cells, dictating their fate.

Various biofluids and, more importantly, sera have been investigated as promising biomarkers for many diseases, including diabetes^57^. This would be particularly relevant if predictions of the response to treatment could be obtained. We have herein identified in T2DM patients criteria to distinguish between subjects with effective and ineffective sEV by analysing their TGFβ and miR-130a content. We have also provided evidence that TGFβ and miR-130a sEV content can predict sEV ineffectiveness in all patient’s groups. In particular, combining data from TGFβ and miR-130a ‘in series’ has demonstrated that the test possesses good specificity (75% with IC: 95% vs 50%) and sensitivity (82.4% with IC: 95% vs 94%) in identifying individuals with ‘true ineffective sEV’. Our study therefore suggests that TGFβ and miR-130a content may be a promising non-invasive detection method for the recognition of individuals, especially those with T2DM, that may benefit from autologous sEV treatment. A larger study is required to reinforce this observation.

Of note, all obese and 5 out of 9 obese/diabetic patients displayed low sEV-miR-130a content. Low miR-130 and high peroxisome proliferator-activated receptor gamma (PPARγ) were detected in adipose tissue from obese, but not lean women^58^. This suggests that miR-130a sEV content may predict adipose tissue perturbation, and thereby may be considered a specific signature of adipose tissue dysfunction in obese subjects. Finally, a significant negative correlation between PPARγ and circulating miR-130 level was reported in obese patients with colorectal cancer^59^. Therefore, to exploit miR-130 sEV content as a potential biomarker of colorectal cancer susceptibility in obese patients should be a further future challenge.

One limitation of the study is the small clinical sample, requiring external validation in cohorts from other/multiple centres. Nevertheless, this is the first study which reports a sensitive and specific assay with which to evaluate sEV effectiveness in patients with different sEV angiogenic properties. Moreover, our results provides the rational to explore, in different cluster of patients at high cardiovascular risk, and in particular in T2DM patients, autologous sEV administration to improve angiogenesis. Since EV immunogenicity, isolation and scalability are still a matter of debate, to move from preclinical to clinical studies, autologous samples would avoid concerns on EV safety, isolation and storage.

## Methods

### Patients

Thirty-six patients at high cardiovascular risk and nine sex-matched healthy volunteers were included in the study. In detail, nine T2DM patients (D:n = 9), nine obese individuals (O:n = 9), nine obese T2DM patients (OD:n = 9) with no previous cardiovascular events, and nine ischemic patients, some of whom display diabetes and/or obesity while others do not, that were undergoing surgical treatment for hind limb ischemia in our Clinic (IC:n = 9) were herein examined. No T2DM patients received insulin treatment. No T2DM, obese, obese/T2DM patients were under statin treatment, whereas all IC patients received statins. Patient clinical data are reported in Table 1. All human experiments were performed in accordance with European Guidelines and policies, and were approved by the Ethical Committee of the University of Turin. Serum from all patients was obtained after admission to our Clinics (D, O, OD) and before surgery, for ischemic patients (IC). Written informed consent was obtained from all patients. Human serum from healthy donors (n = 9) was provided by the Blood Bank of ‘Città della Salute e della Scienza di Torino’, after informed consent and approval by the internal Review Board of the Blood Bank. Approval was obtained from Ethical Committee of the of ‘Città della Salute e della Scienza di Torino’ (protocol no: 0007814).

### Study approval

Animal studies were conducted in accordance with the Italian National Institute of Health Guide for the Care and Use of Laboratory Animals. Approval was obtained from Ethical Committee of the Italian National Institute of Health (protocol no: 490/2105-PR). Mice were housed according to the Guidelines of the Federation of European Laboratory Animal Science Association and the Ethical Committee of the University of Turin. All experiments were performed according to relevant guidelines and regulations.

### Isolation of EVs from sera

Blood was obtained via venipuncture, with 9 ml serum/each participant being collected and stored at −80 °C. After thawing, total EVs were isolated and purified by Ultracentrifugation at 100,000 × g for 2 h, which was preceded by centrifugation at 3000 g to remove debris. Pellets were washed once with PBS and centrifuged at 100,000 × g, 4 °C for 1 h. Samples were either used fresh, or thawed after being stored at −80°^21^.

### Nanoparticle-tracking analyses

EVs underwent nanoparticle tracking analyses (NTA) that were performed using the NanoSight LM10 system (NanoSight Ltd., Amesbury, UK), to delineate their size and profile. All acquisitions were carried out at a Camera level setting of 14, and three videos of 30 s duration were recorded for each sample. sEV were diluted (1: 1000) in 1 ml of vesicle-free physiological solution (Fresenius Kabi, Runcorn, UK) and analysed as previously described^21^.

### FACS analysis

sEV FACS analyses were performed using GUAVA (Guava easyCyte™ Flow Cytometer, Millipore, Germany) as previously described^60^. The detection of nanoparticles was facilitated by the use of flow cytometry beads (Aldehyde/Sulfate latex 4% w/v 4 µm, Invitrogen, Germany) and APC-, PE- and FITC-conjugated antibodies directed to CD42b, VEGFR3 and CD144, (Dako Denmark A/S, Copenhagen, Denmark). FITC and PE mouse non-immune Isotypic IgG (Beckton Dickinson, Franklin Lakes, NJ) served as controls. EVs were incubated with each antibody, or isotype control antibody at 4 °C in 100 μl PBS containing 0.1% bovine serum albumin, and analyzed. EVs were detected mainly below the forward scatter signal corresponding to 1 mm beads (data not shown). All samples were gated on the basis of negative controls and compensated appropriately prior to analyses. Population percentages/numbers were generated for gated populations from each experiment using Guava Incyte software (Millipore).

### sEV angiogenic assay

Primary macrovascular endothelial cells (ECs) and microvascular endothelial cells (HMEC) were purchased from Lonza (Basel, Switzerland) and cultured as indicated in the manufacturer’s instructions. *In-vitro* tests of potency and *in-vivo* angiogenesis were performed as previously described^21^. Briefly, all the *in-vitro* study were performed administering 5 × 10^4^ sEV/target cells. BrdU and *in-vitro* tubulogenesis assays were used to assess single sample sEV pro-angiogenic activity. EVs from of all the analysed groups were classified as effective or ineffective sEV (% cut-off value = 50). *In-vivo* angiogenesis was assessed by measuring the growth of blood vessels as previously described^60,61^. Briefly, ECs (1 × 10^6^ cells/injection) were incubated overnight with sEV (5 × 10^10^ EVs per 1 × 10^6^ ECs). After incubation, sEV-treated ECs were mixed with Matrigel immediately before their subcutaneous injection. Male severe combined immunodeficiency (SCID) mice (6 weeks old/24 g) (3/each group) were then injected subcutaneously. Since the female estrus cycle could introduce unexpected variables, only male mice have been used in this study. An equal number of non-stimulated ECs were used as a negative control. The Matrigel plugs, containing wheals, were recovered on day 7 and then fixed and stained using the trichrome stain method. The vessel lumen area was determined as previously described^21^. Two different blind observers performed the *in-vivo* analysis. Animals have been used after *in-vitro* studies which have help to obtain the preliminary and key information to select the most effective therapeutic tools to be validated *in-vivo*. For animal experiments three mice have been placed in each cage. Housing of animals have been standardised to the following procedures: Room temperature: 22+/− 1 °C; U.R: 55+/− 10%; 12/12 light /dark cycle. Caging condition: SPF animal facilities. Group housing. Environmental Enrichment. Cage size 350 cm^2^ minimum, 100 cm^2^ per animal minimum. Animals have been anesthetised by gas anaesthesia prior to any “surgical” procedure. Animals have been sacrificed when they show signs of distress. We were careful in selecting a number of animals that was sufficient to provide statistically significant results while not unnecessary wasting them.

### TGFβ ELISA assay

sEV (2.5 × 10^8^ particles) were lysed from all healthy subjects and patients to evaluate TGFβ EV content (performed in triplicate). A solid phase sandwich Enzyme Linked-Immuno-Sorbent Assay (ELISA, Invitrogen Multispecies TGF-β1 kit, Germany) was used, according to manufacturer’s instructions.

### miR Screening

sEV miRNome was assessed via the RT-PCR of 1140 microRNA, using miRNome microRNA Profilers QuantiMir (SBI, System Biosciences). The kit includes assays in pre-formatted plates for human microRNAs with three endogenous reference RNA controls (U6, RNU43 and Hm/Ms/Rt U1) on each plate. We profiled the miRNome of effective (n = 3) and ineffective sEV (n = 3)^21^ collected from the serum of healthy subjects. miRNA Ct values were extrapolated for each EV sample analysed. The Ct averages from the different samples (n = 3) of both EV populations were normalised to endogenous controls and converted into 2^−(∆Ct)^ values. miRNA validation was performed using the miScript SYBR Green PCR Kit (Qiagen, Valencia, CA, USA). 100 ng of input RNA were reverse transcribed and the cDNA used to detect and quantify miRNAs of interest. Experiments were run in triplicate using 3 ng of cDNA/each reaction as described in the protocol (Qiagen). The following miRNAs were screened in all patient-derived EVs: miR-126, miR-21, miR-296-3p, miR-210, miR-130a, miR-27a, miR-29a and miR-191. qRT-PCR data were normalised using RNU6B and RNU43 as internal controls.

### Pathways and miR target prediction analysis of EV content

The web-based program DIANA-mirPath was used for EV miRNA target prediction and biological pathway enrichment analyses, as previously reported^62^. The algorithm microT-CDS was chosen to predict EV-derived miRNA targets using a default threshold (microT = 0.8). Only biological pathways with a P value < 0.01 to all known Kyoto Encyclopaedia of Genes and Genomes (KEGG) pathways were considered as significantly enriched. IPA was used to predict the target genes for miR-130a. We set up the miRNA Target Filter tool on IPA (Qiagen: http://www.qiagen-bioinformatics.com/products/ingenuity-pathway-analysis/) to associate miR-130a with predicted mRNA targets.

### miR-130a target(s) validation

Transfection of mimic-miR-130a (10 nM) was performed on ECs using HiPerfect Transfection method (Qiagen, Valencia, USA)^63^. Total RNA from untreated ECs, ECs transfected with mimic-miR-130a, treated with sEV with high (D17, D18, D20) or low miR-130a content (D1, D2, D4) was extracted using All-in-One Purification kit (Norgen, Thorold, ON Canada). miR-Scramble (Scr) was used as transfection reference sample. The expression of miR-130a was evaluated by RT-PCR^63^. miR-130a targets were analyzed by Western Blot analysis. Proteins were obtained using RIPA buffer, as previously described^63^. Anti-KDR (1:200, Santa Cruz), anti-ROCK1 (1:200, Abcam), and anti-HOXA5 (1:200, Abcam) antibody, together with anti-βactin, were used (1:200, Santa Cruz).

### Statistical analysis

Data were analysed using the GraphPad Prism 6.0 Demo program. Results are expressed as means ± SD or ±SEM, unless otherwise reported. 1-way ANOVA, followed by Tukey’s *post hoc* or multiple comparison, Student t tests for 2-group comparison and Newman-Keuls Multiple Comparison Test where appropriate were used. The cut-off for statistical significance was set at p < 0.05 (*p < 0.05, **p < 0.01, ***p < 0.001). Our data passed normality and equal variance tests. According to our previous data, the minimum sample size that has permitted us to detect a 40% difference between the experimental groups, with 90% power and a probability level of 0.05 in a two-tailed hypothesis, was n = 3 mice/group.

ROC analysis: Principal data are presented as means, standard deviations (SD), median and 95% confidence intervals for the two investigated groups - ‘True effective sEV’/‘True ineffective sEV’ considered as the Reference Standard (RS). The achievement of RS was evaluated using ROC curves^31,64–67^ to evaluate predictability for miR-130a and TGFβ. sEV were classified into the following categories according to the Ct value of miR-130a and pg/ml of TGFβ:

1. sEV displaying a miR-130a Ct value ≥ 30 were considered to be ineffective sEV;
2. sEV displaying TGFβ content < 1100 pg/ml were considered to be ineffective sEV.

Finally, we evaluated the ‘goodness’ of the cut-off score used for the two measures to predict ‘True ineffective sEV’ as defined by the RS. The predictive capacity was evaluated both for each of the two measures individually and by combining the two measures ‘in series’ (sEV ineffective for both measures were considered ‘ineffective sEV’). The analysis was based on Sensitivity (Se), Specificity (Sp), positive Likelihood ratio (LH+) (Probability of identifying a ‘true ineffective sEV’ as an ‘ineffective sEV’ compared to that of identifying a ‘true effective sEV’ as an ‘ineffective sEV’) and relative 95% Confidence Intervals values^68,69^.

## Supplementary information

Supplementary Material

## References

[1] Bastien M, Poirier P, Lemieux I, Després JP (2014). Overview of epidemiology and contribution of obesity to cardiovascular disease. Prog. Cardiovasc. Dis..

[2] Deregibus Maria Chiara, Cantaluppi Vincenzo, Calogero Raffaele, Lo Iacono Marco, Tetta Ciro, Biancone Luigi, Bruno Stefania, Bussolati Benedetta, Camussi Giovanni (2007). Endothelial progenitor cell–derived microvesicles activate an angiogenic program in endothelial cells by a horizontal transfer of mRNA. Blood.

[3] Camussi G, Deregibus MC, Cantaluppi V (2013). Role of stem-cell-derived microvesicles in the paracrine action of stem cells. Biochem. Soc. Trans..

[4] Tetta C, Ghigo E, Silengo L, Deregibus MC, Camussi G (2013). Extracellular vesicles as an emerging mechanism of cell-to-cell communication. Endocrine.

[5] Yuana Y, Sturk A, Nieuwland R (2013). Extracellular vesicles in physiological and pathological conditions. Blood Rev..

[6] Robbins PD, Dorronsoro A, Booker CN (2016). Regulation of chronic inflammatory and immune processes by extracellular vesicles. J. Clin. Invest..

[7] Shanmuganathan M, Vughs J, Noseda M, Emanueli C (2018). Exosomes: Basic Biology and Technological Advancements Suggesting Their Potential as Ischemic Heart Disease Therapeutics. Front. Physiol..

[8] Belting M, Christianson HC (2015). Role of exosomes and microvesicles in hypoxia-associated tumour development and cardiovascular disease. Journal of Internal Medicine.

[9] Faure V (2006). Elevation of circulating endothelial microparticles in patients with chronic renal failure. J. Thromb. Haemost..

[10] Mulcahy LA, Pink RC, Carter DRF (2014). Routes and mechanisms of extracellular vesicle uptake. J. Extracell. Vesicles.

[11] Gidlof O (2013). Platelets activated during myocardial infarction release functional miRNA, which can be taken up by endothelial cells and regulate ICAM1 expression. Blood.

[12] Pathan M (2018). Vesiclepedia 2019: a compendium of RNA, proteins, lipids and metabolites in extracellular vesicles. Nucleic Acids Res..

[13] Catalanotto, C., Cogoni, C. & Zardo, G. MicroRNA in Control of Gene Expression: An Overview of Nuclear Functions. *Int*. *J*. *Mol*. *Sci*. **17** (2016). 10.3390/ijms17101712 PMC5085744 27754357

[14] Kondkar AA, Abu-Amero KK (2015). Utility of circulating microRNAs as clinical biomarkers for cardiovascular diseases. Biomed Res. Int..

[15] Pan Y (2014). Platelet-secreted microRNA-223 promotes endothelial cell apoptosis induced by advanced glycation end products via targeting the insulin-like growth factor 1 receptor. J. Immunol..

[16] Hosseinkhani B, Kuypers S, van den Akker NMS, Molin DGM, Michiels L (2018). Extracellular Vesicles Work as a Functional Inflammatory Mediator Between Vascular Endothelial Cells and Immune Cells. Front. Immunol..

[17] Zhang H (2018). Serum exosomes mediate delivery of arginase 1 as a novel mechanism for endothelial dysfunction in diabetes. Proc. Natl. Acad. Sci..

[18] Togliatto G (2018). PDGF-BB Carried by Endothelial Cell-Derived Extracellular Vesicles Reduces Vascular Smooth Muscle Cell Apoptosis in Diabetes. Diabetes.

[19] Louise Schacht Revenfeld, A. *et al*. *Diagnostic and Prognostic Potential of Extracellular Vesicles in Peripheral Blood*. *Clinical Therapeutics* **36** (2014). 10.1016/j.clinthera.2014.05.008 24952934

[20] Bei Y (2017). Exercise-induced circulating extracellular vesicles protect against cardiac ischemia–reperfusion injury. Basic Res. Cardiol..

[21] Cavallari C (2017). Serum-derived extracellular vesicles (EVs) impact on vascular remodeling and prevent muscle damage in acute hind limb ischemia. Sci. Rep..

[22] Khurana R, Simons M, Martin JF, Zachary IC (2005). Role of angiogenesis in cardiovascular disease: A critical appraisal. Circulation.

[23] Deveza L, Choi J, Yang F (2012). Therapeutic angiogenesis for treating cardiovascular diseases. Theranostics.

[24] Anand S (2013). A brief primer on microRNAs and their roles in angiogenesis. Vasc. Cell.

[25] Yin K-J, Hamblin M, Chen YE (2015). Angiogenesis-regulating microRNAs and Ischemic Stroke. Curr. Vasc. Pharmacol..

[26] Shalaby SM, El-Shal AS, Shoukry A, Khedr MH, Abdelraheim N (2016). Serum miRNA-499 and miRNA-210: A potential role in early diagnosis of acute coronary syndrome. IUBMB Life.

[27] Mosch, B., Reissenweber, B., Neuber, C. & Pietzsch, J. Eph receptors and ephrin ligands: Important players in angiogenesis and tumor angiogenesis. *J*. *Oncol*. **2010** (2010). 10.1155/2010/135285 PMC2836134 20224755

[28] Montalvo J (2013). Rock1 & 2 Perform Overlapping and Unique Roles in Angiogenesis and Angiosarcoma Tumor Progression. Curr Mol Med.

[29] Yoshiji H (1999). KDR/Flk-1 is a major regulator of vascular endothelial growth factor–induced tumor development and angiogenesis in murine hepatocellular carcinoma cells. Hepatology.

[30] Chen Y, Gorski DH (2008). Regulation of angiogenesis through a microRNA (miR-130a) that down-regulates antiangiogenic homeobox genes GAX and HOXA5. Blood.

[31] Gai C (2018). Salivary extracellular vesicle-associated miRNAs as potential biomarkers in oral squamous cell carcinoma. BMC Cancer.

[32] Einarson TR, Acs A, Ludwig C, Panton UH (2018). Prevalence of cardiovascular disease in type 2 diabetes: a systematic literature review of scientific evidence from across the world in 2007-2017. Cardiovasc. Diabetol..

[33] Kolluru GK, Bir SC, Kevil CG (2012). Endothelial dysfunction and diabetes: effects on angiogenesis, vascular remodeling, and wound healing. Int. J. Vasc. Med..

[34] Zeng H, Chen J-X (2019). Microvascular Rarefaction and Heart Failure With Preserved Ejection Fraction. Front. Cardiovasc. Med..

[35] Heusch G (2014). Cardiovascular remodelling in coronary artery disease and heart failure. Lancet.

[36] Gili M, Orsello A, Gallo S, Brizzi MF (2013). Diabetes-associated macrovascular complications: cell-based therapy a new tool?. Endocrine.

[37] Cohn JN, Ferrari R, Sharpe N (2000). Cardiac remodeling—concepts and clinical implications: a consensus paper from an international forum on cardiac remodeling. J. Am. Coll. Cardiol..

[38] Kourembanas S (2015). Exosomes: Vehicles of Intercellular Signaling, Biomarkers, and Vectors of Cell Therapy. Annu. Rev. Physiol..

[39] Batrakova EV, Kim MS (2015). Using exosomes, naturally-equipped nanocarriers, for drug delivery. J. Control. Release.

[40] Oh S-J (1997). VEGF and VEGF-C: Specific Induction of Angiogenesis and Lymphangiogenesis in the Differentiated Avian Chorioallantoic Membrane. Dev. Biol..

[41] Wu H (2018). Epsin deficiency promotes lymphangiogenesis through regulation of VEGFR3 degradation in diabetes. J. Clin. Invest..

[42] Kim, A., Shah, A. S. & Nakamura, T. Extracellular Vesicles: A Potential Novel Regulator of Obesity and Its Associated Complications. *Child*. *(Basel*, *Switzerland)* **5** (2018). 10.3390/children5110152 PMC6262587 30445758

[43] Togliatto G (2015). Obesity reduces the pro-angiogenic potential of adipose tissue stem cell-derived extracellular vesicles (EVs) by impairing miR-126 content: impact on clinical applications. Int. J. Obes..

[44] Chiva-Blanch G (2016). Microparticle Shedding by Erythrocytes, Monocytes and Vascular Smooth Muscular. Cells Is Reduced by Aspirin in Diabetic Patients. Rev. Esp. Cardiol. (Engl. Ed)..

[45] Sabatier F (2002). Type 1 And Type 2 Diabetic Patients Display Different Patterns of Cellular Microparticles. Diabetes.

[46] Chen J (2014). Proangiogenic compositions of microvesicles derived from human umbilical cord mesenchymal stem cells. PLoS One.

[47] Zhang B (2015). Human umbilical cord mesenchymal stem cell exosomes enhance angiogenesis through the Wnt4/beta-catenin pathway. Stem Cells Transl. Med..

[48] Chiva-Blanch G (2017). Monocyte-derived circulating microparticles (CD14(+), CD14(+)/CD11b(+) and CD14(+)/CD142(+)) are related to long-term prognosis for cardiovascular mortality in STEMI patients. Int. J. Cardiol..

[49] Devlin C, Greco S, Martelli F, Ivan M (2011). miR-210: More than a silent player in hypoxia. IUBMB Life.

[50] Eguchi A (2016). Circulating adipocyte-derived extracellular vesicles are novel markers of metabolic stress. J. Mol. Med. (Berl)..

[51] Murakami T (2007). Impact of weight reduction on production of platelet-derived microparticles and fibrinolytic parameters in obesity. Thromb. Res..

[52] Pardo F, Villalobos-Labra R, Sobrevia B, Toledo F, Sobrevia L (2018). Extracellular vesicles in obesity and diabetes mellitus. Mol. Aspects Med..

[53] Sridurongrit S, Larsson J, Schwartz R, Ruiz-Lozano P, Kaartinen V (2008). Signaling via the Tgf-beta type I receptor Alk5 in heart development. Dev. Biol..

[54] Pohlers D (2009). TGF-β and fibrosis in different organs - molecular pathway imprints. Biochim. Biophys. Acta - Mol. Basis Dis..

[55] Evrard SM (2012). The profibrotic cytokine transforming growth factor-β1 increases endothelial progenitor cell angiogenic properties. J. Thromb. Haemost..

[56] Guduric-Fuchs J (2012). Selective extracellular vesicle-mediated export of an overlapping set of microRNAs from multiple cell types. BMC Genomics.

[57] Guay C, Regazzi R (2013). Circulating microRNAs as novel biomarkers for diabetes mellitus. Nat. Rev. Endocrinol..

[58] Lee EK (2011). miR-130 Suppresses Adipogenesis by Inhibiting Peroxisome Proliferator-Activated Receptor Expression. Mol. Cell. Biol..

[59] Motawi TK, Shaker OG, Ismail MF, Sayed NH (2017). Peroxisome Proliferator-Activated Receptor Gamma in Obesity and Colorectal Cancer: The Role of Epigenetics. Sci. Rep..

[60] Lopatina T (2014). Platelet-derived growth factor regulates the secretion of extracellular vesicles by adipose mesenchymal stem cells and enhances their angiogenic potential. Cell Commun. Signal..

[61] Couffinhal T (1998). Mouse model of angiogenesis. Am. J. Pathol..

[62] Collino F (2017). Exosome and Microvesicle-Enriched Fractions Isolated from Mesenchymal Stem Cells by Gradient Separation Showed Different Molecular Signatures and Functions on Renal Tubular Epithelial Cells. Stem Cell Rev..

[63] Cavallari C (2019). Online Hemodiafiltration Inhibits Inflammation-Related Endothelial Dysfunction and Vascular Calcification of Uremic Patients Modulating miR-223 Expression in Plasma Extracellular Vesicles. J. Immunol..

[64] Grund B, Sabin C (2010). Analysis of biomarker data: logs, odds ratios, and receiver operating characteristic curves. Curr. Opin. HIV AIDS.

[65] Hanley JA, McNeil BJ (1982). The meaning and use of the area under a receiver operating characteristic (ROC) curve. Radiology.

[66] Zweig MH, Campbell G (1993). Receiver-operating characteristic (ROC) plots: a fundamental evaluation tool in clinical medicine. Clin. Chem..

[67] Altman, D. G. *Practical statistics for medical research*. (Chapman & Halls, 1990).

[68] Harper R, Reeves B (1999). Reporting of precision of estimates for diagnostic accuracy: a review. BMJ.

[69] Nam J-M (1995). Confidence Limits for the Ratio of Two Binomial Proportions Based on Likelihood Scores: Non-Iterative Method. Biometrical J..

