# Supplementary material for: miR-130a and Tgfβ Content in Extracellular Vesicles Derived from the Serum of Subjects at High Cardiovascular Risk Predicts their In-Vivo Angiogenic Potential
Source: Sci Rep. 2020 Jan 20;10:706. doi: 10.1038/s41598-019-55783-7 (PMC6971269; doi:10.1038/s41598-019-55783-7)
Supplement: Supplementary file 1 — Supplementary Material [file 41598_2019_55783_MOESM1_ESM.docx]

**MIR-130A AND TGFβ CONTENT IN EXTRACELLULAR VESICLES DERIVED FROM THE SERUM OF SUBJECTS AT HIGH CARDIOVASCULAR RISK PREDICTS THEIR *IN-VIVO* ANGIOGENIC POTENTIAL**

Claudia Cavallari, Federico Figliolini, Marta Tapparo, Massimo Cedrino, Alessandra Trevisan, Lorenza Positello, Pietro Rispoli, Anna Solini, Giuseppe Migliaretti, Giovanni Camussi, Maria Felice Brizzi

**SUPPLEMENTARY MATERIAL**

|  | ***subject*** | ***particle sEV/ml*** |
| --- | --- | --- |
| ***HEALTHY*** | 1 | 8.21E+08 |
|  | 2 | 6.24E+08 |
|  | 3 | 1.90E+09 |
|  | 4 | 1.82E+09 |
|  | 5 | 1.10E+09 |
|  | 6 | 2.05E+07 |
|  | 7 | 2.05E+08 |
|  | 8 | 2.75E+08 |
|  | 9 | 8.22E+08 |

|  | ***subject*** | ***particle sEV/ml*** |
| --- | --- | --- |
|  | 1 | 6.00E+08 |
|  | 2 | 4.75E+08 |
| ***DIABETIC*** | 4 | 1.00E+09 |
|  | 5 | 8.10E+08 |
|  | 7 | 1.40E+08 |
|  | 8 | 1.36E+08 |
|  | 17 | 1.55E+12 |
|  | 18 | 2.98E+11 |
|  | 20 | 2.95E+11 |

|  | ***subject*** | ***particle sEV/ml*** |
| --- | --- | --- |
|  | 12 | 7.75E+11 |
| ***OBESE*** | 15 | 9.10E+11 |
|  | 19 | 6.50E+11 |
|  | 22 | 4.97E+11 |
|  | 31 | 3.45E+11 |
|  | 32 | 4.01E+11 |
|  | 33 | 3.83E+11 |
|  | 34 | 1.97E+11 |
|  | 35 | 3.38E+11 |

|  | ***subject*** | ***particle sEV/ml*** |
| --- | --- | --- |
| ***OBESE AND DIABETIC*** | 3 | 6.39E+07 |
|  | 6 | 6.20E+08 |
|  | 9 | 6.40E+08 |
|  | 10 | 2.90E+08 |
|  | 13 | 5.57E+11 |
|  | 14 | 1.84E+12 |
|  | 16 | 1.80E+12 |
|  | 21 | 1.39E+11 |
|  | 36 | 2.00E+11 |

|  | ***subject*** | ***particle sEV/ml*** |
| --- | --- | --- |
|  | 11 | 1.65E+11 |
|  | 23 | 6.62E+11 |
| ***ISCHEMIC*** | 24 | 8.20E+11 |
|  | 25 | 6.80E+11 |
|  | 26 | 4.75E+11 |
|  | 27 | 6.39E+10 |
|  | 28 | 1.02E+12 |
|  | 29 | 8.06E+11 |
|  | 30 | 6.19E+11 |

**Table S1.** NTA relative amounts of sEV in 9 healthy subjects, 9 diabetic, 9 obese, 9 obese/diabetic and 9 ischemic patients.





**Fig. S1** Representative FACS analysis of sEV from each group of patients. The expression of CD42b, CD144 and VEGFR3 is reported. D=T2DM; O= Obese; OD= obese/T2DM; IC= Ischemic patients. (n= 9 patients/group)

|  | ***subject*** | ***% potency test*** | |
| --- | --- | --- | --- |
| ***HEALTHY*** | 1 | 53.9 | ±1.2 |
|  | 2 | 9.4 | ±1.0 |
|  | 3 | 106.7 | ±0.6 |
|  | 4 | 69.1 | ±0.8 |
|  | 5 | 31.6 | ±2.1 |
|  | 6 | 24.4 | ±3.0 |
|  | 7 | 54.1 | ±2.9 |
|  | 8 | 57.4 | ±1.3 |
|  | 9 | 81.0 | ±0.9 |

|  | ***subject*** | ***% potency test*** | |
| --- | --- | --- | --- |
|  | 1 | 34.1 | ±0.8 |
|  | 2 | 53.6 | ±0.3 |
| ***DIABETIC*** | 4 | 47.6 | ±0.6 |
|  | 5 | 12.2 | ±0.9 |
|  | 7 | 41.1 | ±2.1 |
|  | 8 | 19.1 | ±2.5 |
|  | 17 | 103.8 | ±2.3 |
|  | 18 | 99.4 | ±3.2 |
|  | 20 | 97.1 | ±3.5 |

|  | ***subject*** | ***% potency test*** | |
| --- | --- | --- | --- |
|  | 12 | 53.7 | ±4.0 |
| ***OBESE*** | 15 | 32.5 | ±0.6 |
|  | 19 | 81.1 | ±3.4 |
|  | 22 | 47.2 | ±2.1 |
|  | 31 | 48.2 | ±2.1 |
|  | 32 | 52.0 | ±1.5 |
|  | 33 | 44.4 | ±1.8 |
|  | 34 | 68.1 | ±1.4 |
|  | 35 | 70.7 | ±1.6 |

|  | ***subject*** | ***% potency test*** | |
| --- | --- | --- | --- |
| ***OBESE AND DIABETIC*** | 3 | 53.3 | ±2.1 |
|  | 6 | 53.6 | ±0.9 |
|  | 9 | 59.6 | ±1.8 |
|  | 10 | 68.6 | ±1.1 |
|  | 13 | 40.4 | ±1.4 |
|  | 14 | 79.6 | ±2.3 |
|  | 16 | 115.3 | ±1.9 |
|  | 21 | 53.8 | ±1.1 |
|  | 36 | 103.0 | ±4.5 |

|  | ***subject*** | ***% potency test*** | |
| --- | --- | --- | --- |
|  | 11 | 34.8 | ±0.7 |
|  | 23 | 63.2 | ±0.9 |
| ***ISCHEMIC*** | 24 | 28.9 | ±1.1 |
|  | 25 | 73.0 | ±1.1 |
|  | 26 | 39.8 | ±2.1 |
|  | 27 | 57.7 | ±2.8 |
|  | 28 | 16.1 | ±2.4 |
|  | 29 | 63.2 | ±1.8 |
|  | 30 | 79.9 | ±0.3 |

**Table S2.** **sEV potency test.** sEV samples from patients and healthy donors evaluated by in vitro angiogenesis assay and in vitro proliferation assay on ECs. The average represents the mean value of results of both assays ± SD (see Methods). sEV with an average value exceeding 50% have been considered effective sEV otherwise they are ineffective.

| ***subjects*** | ***TGFβ (pg/ml)*** | |
| --- | --- | --- |
| *1 H* | 1222.60 | ±22.3 |
| *2 H* | 999.20 | ±18.2 |
| *3 H* | 1181.60 | ±15.1 |
| *4 H* | 1299.60 | ±10.9 |
| *5 H* | 1099.20 | ±10.1 |
| *6 H* | 1156.60 | ±22.2 |
| *7 H* | 1311.60 | ±11.3 |
| *8 H* | 1107.20 | ±19.2 |
| *9 H* | 1111.60 | ±17.4 |
| *1 D* | 1000.20 | ±16.5 |
| *2 D* | 1100.00 | ±4.3 |
| *4 D* | 1000.20 | ±5.5 |
| *5 D* | 1001.32 | ±9.8 |
| *7 D* | 981.30 | ±8.7 |
| *8 D* | 1100.54 | ±16.7 |
| *17 D* | 1499.23 | ±21.1 |
| *18 D* | 1407.00 | ±19.2 |
| *20 D* | 1500.30 | ±16.6 |
| *15 O* | 955.32 | ±8.8 |
| *33 O* | 1024.54 | ±10.3 |
| *22 O* | 1100.00 | ±5.5 |
| *31 O* | 971.30 | ±6.7 |
| *32 O* | 1000.20 | ±23.3 |
| *12 O* | 1100.32 | ±20.1 |
| *34 O* | 1400.30 | ±12.6 |
| *35 O* | 1307.00 | ±19.3 |
| *19 O* | 1599.23 | ±8.9 |
| *13 OD* | 1200.30 | ±15.1 |
| *3 OD* | 998.00 | ±13.0 |
| *6 OD* | 999.00 | ±9.9 |
| *21 OD* | 1023.22 | ±6.7 |
| *9 OD* | 1100.22 | ±17.6 |
| *10 OD* | 963.20 | ±6.6 |
| *14 OD* | 1056.42 | ±6.8 |
| *36 OD* | 985.32 | ±9.7 |
| *16 OD* | 964.23 | ±8.6 |
| *28 IC* | 953.20 | ±23.4 |
| *24 IC* | 999.45 | ±19.9 |
| *11 IC* | 987.56 | ±12.8 |
| *26 IC* | 1023.89 | ±17.4 |
| *27 IC* | 1100.23 | ±9.10 |
| *23 IC* | 1078.56 | ±21.5 |
| *29 IC* | 1178.65 | ±18.3 |
| *25 IC* | 1089.77 | ±12.7 |
| *30 IC* | 1255.89 | ±19.8 |

**Table S3.** **sEV TGFβ pg/ml.** TGFβ pg/ml sEV content in healthy, diabetic, obese, diabetic/obese and ischemic patients. H= Healthy subjects; D = Diabetic patients; O= Obese patients; OD= Obese/Diabetic patients; IC = Ischemic patients.

| ***mature miRNA ID*** | ***Target microRNA Mature Sequence*** | ***Average Ct miR e-sEV*** | | ***Average Ct miR i-sEV*** | |
| --- | --- | --- | --- | --- | --- |
| **hsa-let-7a** | UGAGGUAGUAGGUUGUAUAGUU | 26.75 | ± 3.12 | 30.58 | ± 1.44 |
| **hsa-let-7b** | UGAGGUAGUAGGUUGUGUGGUU | 26.76 | ± 2.17 | 26.93 | ± 2.69 |
| **hsa-let-7c** | UGAGGUAGUAGGUUGUAUGGUU | 24.64 | ± 2.47 | 29.08 | ± 1.19 |
| **hsa-let-7d** | AGAGGUAGUAGGUUGCAUAGUU | 24.65 | ± 3.40 | 28.30 | ± 2.07 |
| **hsa-let-7e** | UGAGGUAGGAGGUUGUAUAGUU | 24.25 | ± 3.56 | 30.71 | ± 5.99 |
| **hsa-let-7f** | UGAGGUAGUAGAUUGUAUAGUU | 24.29 | ± 2.89 | 31.26 | ± 5.70 |
| **hsa-let-7g** | UGAGGUAGUAGUUUGUACAGUU | 26.10 | ± 3.46 | 30.53 | ± 1.62 |
| **hsa-let-7i** | UGAGGUAGUAGUUUGUGCUGUU | 27.73 | ± 2.33 | 30.53 | ± 4.43 |
| **hsa-miR-1** | UGGAAUGUAAAGAAGUAUGUAU | 28.89 | ± 1.36 | 18.98 | ± 7.47 |
| **hsa-miR-7** | UGGAAGACUAGUGAUUUUGUUGU | 29.19 | ± 1.44 | 33.09 | ± 0.82 |
| **hsa-miR-9** | UCUUUGGUUAUCUAGCUGUAUGA | 29.03 | ± 1.33 | 33.64 | ± 2.12 |
| **hsa-miR-10a** | UACCCUGUAGAUCCGAAUUUGUG | 28.41 | ± 2.67 | 30.33 | ± 2.75 |
| **hsa-miR-10b** | UACCCUGUAGAACCGAAUUUGUG | 29.71 | ± 1.69 | 32.00 | ± 6.18 |
| **hsa-miR-15a** | UAGCAGCACAUAAUGGUUUGUG | 27.35 | ± 5.70 | 24.68 | ± 2.27 |
| **hsa-miR-15b** | UAGCAGCACAUCAUGGUUUACA | 28.37 | ± 4.43 | 26.49 | ± 2.33 |
| **hsa-miR-16** | UAGCAGCACGUAAAUAUUGGCG | 21.73 | ± 4.16 | 20.10 | ± 0.00 |
| **hsa-miR-17** | CAAAGUGCUUACAGUGCAGGUAG | 26.28 | ± 2.44 | 25.29 | ± 1.49 |
| **hsa-miR-18a** | UAAGGUGCAUCUAGUGCAGAUAG | 25.01 | ± 2.56 | 28.30 | ± 4.07 |
| **hsa-miR-18b** | UAAGGUGCAUCUAGUGCAGUUAG | 26.84 | ± 3.66 | 30.77 | ± 2.00 |
| **hsa-miR-19a** | UGUGCAAAUCUAUGCAAAACUGA | 24.82 | ± 2.27 | 24.44 | ± 0.49 |
| **hsa-miR-19b** | UGUGCAAAUCCAUGCAAAACUGA | 24.52 | ± 1.95 | 31.84 | ± 4.16 |
| **hsa-miR-20a** | UAAAGUGCUUAUAGUGCAGGUAG | 26.34 | ± 3.32 | 27.63 | ± 2.44 |
| **hsa-miR-20b** | CAAAGUGCUCAUAGUGCAGGUAG | 27.79 | ± 2.40 | 26.73 | ± 2.56 |
| **hsa-miR-21** | UAGCUUAUCAGACUGAUGUUGA | 30.30 | ± 0.21 | 29.22 | ± 0.11 |
| **hsa-miR-22** | AAGCUGCCAGUUGAAGAACUGU | 27.86 | ± 4.79 | 26.25 | ± 5.78 |
| **hsa-miR-23a** | AUCACAUUGCCAGGGAUUUCC | 25.44 | ± 4.35 | 24.71 | ± 4.00 |
| **hsa-miR-23b** | AUCACAUUGCCAGGGAUUACC | 24.79 | ± 4.40 | 25.97 | ± 1.01 |
| **hsa-miR-24** | UGGCUCAGUUCAGCAGGAACAG | 26.19 | ± 3.42 | 26.90 | ± 3.71 |
| **hsa-miR-25** | CAUUGCACUUGUCUCGGUCUGA | 21.90 | ± 1.78 | 25.34 | ± 1.04 |
| **hsa-miR-26a** | UUCAAGUAAUCCAGGAUAGGCU | 24.61 | ± 2.49 | 26.09 | ± 4.04 |
| **hsa-miR-26b** | UUCAAGUAAUUCAGGAUAGGU | 24.51 | ± 3.49 | 31.90 | ± 4.88 |
| **hsa-miR-27a** | UUCACAGUGGCUAAGUUCCGC | 28.31 | ± 0.33 | 29.45 | ± 0.42 |
| **hsa-miR-27b** | UUCACAGUGGCUAAGUUCUGC | 21.38 | ± 2.49 | 26.18 | ± 6.33 |
| **hsa-miR-28-3p** | CACUAGAUUGUGAGCUCCUGGA | 27.81 | ± 0.68 | 29.63 | ± 5.68 |
| **hsa-miR-28-5p** | AAGGAGCUCACAGUCUAUUGAG | 25.27 | ± 4.31 | 31.47 | ± 0.16 |
| **hsa-miR-29a** | UAGCACCAUCUGAAAUCGGUUA | 31.61 | ± 0.27 | 30.52 | ± 0.10 |
| **hsa-miR-29b** | UAGCACCAUUUGAAAUCAGUGUU | 26.94 | ± 3.89 | 30.99 | ± 4.66 |
| **hsa-miR-29c** | UAGCACCAUUUGAAAUCGGUUA | 25.51 | ± 4.09 | 29.54 | ± 1.09 |
| **hsa-miR-30a** | UGUAAACAUCCUCGACUGGAAG | 29.84 | ± 4.10 | 26.75 | ± 4.10 |
| **hsa-miR-30b** | UGUAAACAUCCUACACUCAGCU | 21.89 | ± 1.20 | 27.48 | ± 6.73 |
| **hsa-miR-30c** | UGUAAACAUCCUACACUCUCAGC | 28.00 | ± 2.31 | 25.67 | ± 3.50 |
| **hsa-miR-30d** | UGUAAACAUCCCCGACUGGAAG | 25.14 | ± 1.44 | 26.96 | ± 1.00 |
| **hsa-miR-30e** | UGUAAACAUCCUUGACUGGAAG | 26.35 | ± 3.03 | 29.26 | ± 1.77 |
| **hsa-miR-31** | AGGCAAGAUGCUGGCAUAGCU | 30.08 | ± 3.33 | 35.16 | ± 6.33 |
| **hsa-miR-32** | UAUUGCACAUUACUAAGUUGCA | 30.12 | ± 3.78 | 33.91 | ± 5.68 |
| **hsa-miR-33a** | GUGCAUUGUAGUUGCAUUGCA | 29.56 | ± 1.85 | 34.26 | ± 5.70 |
| **hsa-miR-33b** | GUGCAUUGCUGUUGCAUUGC | 36.43 | ± 5.05 | 38.06 | ± 4.81 |
| **hsa-miR-34a** | UGGCAGUGUCUUAGCUGGUUGU | 27.44 | ± 0.37 | 32.04 | ± 4.28 |
| **hsa-miR-34b** | CAAUCACUAACUCCACUGCCAU | 29.55 | ± 1.78 | 31.30 | ± 4.83 |
| **hsa-miR-34c-3p** | AAUCACUAACCACACGGCCAGG | 31.57 | ± 7.32 | 29.11 | ± 2.15 |
| **hsa-miR-34c-5p** | AGGCAGUGUAGUUAGCUGAUUGC | 30.19 | ± 1.90 | 31.23 | ± 5.82 |
| **hsa-miR-92a** | UAUUGCACUUGUCCCGGCCUGU | 23.94 | ± 0.68 | 25.44 | ± 4.91 |
| **hsa-miR-92b** | UAUUGCACUCGUCCCGGCCUCC | 25.61 | ± 1.29 | 26.48 | ± 2.52 |
| **hsa-miR-93** | CAAAGUGCUGUUCGUGCAGGUAG | 24.10 | ± 1.34 | 27.39 | ± 2.52 |
| **hsa-miR-95** | UUCAACGGGUAUUUAUUGAGCA | 29.56 | ± 1.34 | 33.90 | ± 4.76 |
| **hsa-miR-96** | UUUGGCACUAGCACAUUUUUGCU | 36.43 | ± 1.67 | 34.60 | ± 3.83 |
| **hsa-miR-98** | UGAGGUAGUAAGUUGUAUUGUU | 27.44 | ± 1.99 | 35.00 | ± 4.04 |
| **hsa-miR-99a** | AACCCGUAGAUCCGAUCUUGUG | 24.75 | ± 1.66 | 30.99 | ± 1.12 |
| **hsa-miR-99b** | CACCCGUAGAACCGACCUUGCG | 26.95 | ± 2.01 | 30.33 | ± 6.11 |
| **hsa-miR-100** | AACCCGUAGAUCCGAACUUGUG | 25.72 | ± 2.36 | 29.55 | ± 0.92 |
| **hsa-miR-101** | UACAGUACUGUGAUAACUGAA | 25.75 | ± 3.36 | 30.78 | ± 1.28 |
| **hsa-miR-103** | AGCAGCAUUGUACAGGGCUAUGA | 24.72 | ± 1.68 | 26.00 | ± 3.21 |
| **hsa-miR-103-as** | UCAUAGCCCUGUACAAUGCUGCU | 24.92 | ± 1.57 | 32.84 | ± 0.92 |
| **hsa-miR-105** | UCAAAUGCUCAGACUCCUGUGGU | 24.79 | ± 2.68 | 34.66 | ± 8.13 |
| **hsa-miR-106a** | AAAAGUGCUUACAGUGCAGGUAG | 23.83 | ± 2.21 | 25.01 | ± 3.11 |
| **hsa-miR-106b** | UAAAGUGCUGACAGUGCAGAU | 28.59 | ± 6.63 | 27.21 | ± 3.45 |
| **hsa-miR-107** | AGCAGCAUUGUACAGGGCUAUCA | 28.55 | ± 1.96 | 26.67 | ± 1.78 |
| **hsa-miR-122** | UGGAGUGUGACAAUGGUGUUUG | 28.59 | ± 1.76 | 30.87 | ± 1.69 |
| **hsa-miR-124** | UAAGGCACGCGGUGAAUGCC | 28.62 | ± 1.00 | 29.60 | ± 0.60 |
| **hsa-miR-125a-3p** | ACAGGUGAGGUUCUUGGGAGCC | 22.79 | ± 0.95 | 25.37 | ± 0.41 |
| **hsa-miR-125a-5p** | UCCCUGAGACCCUUUAACCUGUGA | 26.92 | ± 1.81 | 30.59 | ± 2.49 |
| **hsa-miR-125b** | UCCCUGAGACCCUAACUUGUGA | 27.90 | ± 2.76 | 23.66 | ± 5.52 |
| **hsa-miR-126** | UCGUACCGUGAGUAAUAAUGCG | 27.40 | ± 0.40 | 28.09 | ± 0.17 |
| **hsa-miR-127-3p** | UCGGAUCCGUCUGAGCUUGGCU | 27.41 | ± 0.83 | 30.16 | ± 5.63 |
| **hsa-miR-127-5p** | CUGAAGCUCAGAGGGCUCUGAU | 30.24 | ± 1.57 | 31.76 | ± 4.53 |
| **hsa-miR-128** | UCACAGUGAACCGGUCUCUUU | 30.02 | ± 1.58 | 31.15 | ± 3.68 |
| **hsa-miR-129-3p** | AAGCCCUUACCCCAAAAAGCAU | 30.24 | ± 1.57 | 36.14 | ± 3.78 |
| **hsa-miR-129-5p** | CUUUUUGCGGUCUGGGCUUGC | 33.32 | ± 5.81 | 32.96 | ± 5.35 |
| **hsa-miR-130a** | CAGUGCAAUGUUAAAAGGGCAU | 28.85 | ± 0.13 | 30.31 | ± 0.22 |
| **hsa-miR-130b** | CAGUGCAAUGAUGAAAGGGCAU | 28.73 | ± 3.01 | 30.87 | ± 3.44 |
| **hsa-miR-132** | UAACAGUCUACAGCCAUGGUCG | 23.43 | ± 2.60 | 24.78 | ± 3.70 |
| **hsa-miR-133a** | UUUGGUCCCCUUCAACCAGCUG | 23.53 | ± 3.60 | 26.93 | ± 1.94 |
| **hsa-miR-133b** | UUUGGUCCCCUUCAACCAGCUA | 27.53 | ± 2.80 | 29.59 | ± 2.49 |
| **hsa-miR-134** | UGUGACUGGUUGACCAGAGGGG | 29.70 | ± 1.91 | 32.64 | ± 5.95 |
| **hsa-miR-135a** | UAUGGCUUUUUAUUCCUAUGUGA | 27.70 | ± 3.91 | 25.71 | ± 4.01 |
| **hsa-miR-135b** | UAUGGCUUUUCAUUCCUAUGUGA | 25.67 | ± 0.73 | 28.97 | ± 1.88 |
| **hsa-miR-136** | ACUCCAUUUGUUUUGAUGAUGGA | 25.97 | ± 3.85 | 36.52 | ± 4.10 |
| **hsa-miR-137** | UUAUUGCUUAAGAAUACGCGUAG | 25.27 | ± 0.89 | 31.23 | ± 4.04 |
| **hsa-miR-138** | AGCUGGUGUUGUGAAUCAGGCCG | 28.10 | ± 1.03 | 30.17 | ± 5.42 |
| **hsa-miR-139-3p** | GGAGACGCGGCCCUGUUGGAGU | 28.68 | ± 2.06 | 32.23 | ± 1.88 |
| **hsa-miR-139-5p** | UCUACAGUGCACGUGUCUCCAG | 28.54 | ± 2.17 | 25.05 | ± 0.75 |
| **hsa-miR-140-3p** | UACCACAGGGUAGAACCACGG | 27.87 | ± 0.95 | 26.93 | ± 3.40 |
| **hsa-miR-140-5p** | CAGUGGUUUUACCCUAUGGUAG | 29.02 | ± 0.86 | 30.52 | ± 3.22 |
| **hsa-miR-141** | UAACACUGUCUGGUAAAGAUGG | 29.12 | ± 2.96 | 33.57 | ± 5.83 |
| **hsa-miR-142-3p** | UGUAGUGUUUCCUACUUUAUGGA | 22.27 | ± 3.57 | 23.53 | ± 4.83 |
| **hsa-miR-142-5p** | CAUAAAGUAGAAAGCACUACU | 23.27 | ± 2.89 | 29.31 | ± 4.56 |
| **hsa-miR-143** | UGAGAUGAAGCACUGUAGCUC | 28.48 | ± 0.50 | 33.15 | ± 4.23 |
| **hsa-miR-144** | UACAGUAUAGAUGAUGUACU | 28.42 | ± 0.60 | 34.21 | ± 1.06 |
| **hsa-miR-145** | GUCCAGUUUUCCCAGGAAUCCCU | 27.34 | ± 4.87 | 33.08 | ± 4.93 |
| **hsa-miR-146a** | UGAGAACUGAAUUCCAUGGGUU | 28.40 | ± 2.74 | 29.12 | ± 2.54 |
| **hsa-miR-146b-3p** | UGCCCUGUGGACUCAGUUCUGG | 29.09 | ± 4.56 | 33.06 | ± 4.90 |
| **hsa-miR-146b-5p** | UGAGAACUGAAUUCCAUAGGCU | 24.30 | ± 4.23 | 29.84 | ± 1.22 |
| **hsa-miR-147** | GUGUGUGGAAAUGCUUCUGC | 27.60 | ± 1.06 | 32.14 | ± 6.38 |
| **hsa-miR-147b** | GUGUGCGGAAAUGCUUCUGCUA | 26.32 | ± 0.78 | 8.00 | ± 1.90 |
| **hsa-miR-148a** | UCAGUGCACUACAGAACUUUGU | 27.13 | ± 0.98 | 28.38 | ± 1.29 |
| **hsa-miR-148b** | UCAGUGCAUCACAGAACUUUGU | 25.84 | ± 0.56 | 28.73 | ± 3.19 |
| **hsa-miR-149** | UCUGGCUCCGUGUCUUCACUCCC | 25.84 | ± 0.87 | 30.52 | ± 3.22 |
| **hsa-miR-150** | UCUCCCAACCCUUGUACCAGUG | 24.09 | ± 5.59 | 27.36 | ± 1.85 |
| **hsa-miR-151-3p** | CUAGACUGAAGCUCCUUGAGG | 27.03 | ± 2.20 | 29.09 | ± 1.01 |
| **hsa-miR-151-5p** | UCGAGGAGCUCACAGUCUAGU | 25.20 | ± 1.51 | 27.60 | ± 1.72 |
| **hsa-miR-152** | UCAGUGCAUGACAGAACUUGG | 26.51 | ± 3.22 | 30.62 | ± 0.68 |
| **hsa-miR-153** | UUGCAUAGUCACAAAAGUGAUC | 26.81 | ± 3.22 | 39.29 | ± 6.11 |
| **hsa-miR-154** | UAGGUUAUCCGUGUUGCCUUCG | 30.35 | ± 0.68 | 34.10 | ± 3.07 |
| **hsa-miR-155** | UUAAUGCUAAUCGUGAUAGGGGU | 30.21 | ± 0.86 | 33.82 | ± 9.28 |
| **hsa-miR-181a** | AACAUUCAACGCUGUCGGUGAGU | 23.06 | ± 1.62 | 25.51 | ± 0.82 |
| **hsa-miR-181b** | AACAUUCAUUGCUGUCGGUGGGU | 23.10 | ± 2.44 | 30.93 | ± 1.69 |
| **hsa-miR-181c** | AACAUUCAACCUGUCGGUGAGU | 22.43 | ± 1.69 | 22.66 | ± 5.17 |
| **hsa-miR-181d** | AACAUUCAUUGUUGUCGGUGGGU | 25.57 | ± 0.89 | 27.31 | ± 1.73 |
| **hsa-miR-182** | UUUGGCAAUGGUAGAACUCACACU | 27.57 | ± 0.76 | 30.04 | ± 3.35 |
| **hsa-miR-183** | UAUGGCACUGGUAGAAUUCACU | 25.49 | ± 1.89 | 30.97 | ± 1.80 |
| **hsa-miR-184** | UGGACGGAGAACUGAUAAGGGU | 30.29 | ± 0.46 | 33.82 | ± 3.01 |
| **hsa-miR-185** | UGGAGAGAAAGGCAGUUCCUGA | 25.70 | ± 1.44 | 27.29 | ± 4.67 |
| **hsa-miR-186** | CAAAGAAUUCUCCUUUUGGGCU | 29.09 | ± 1.48 | 30.61 | ± 4.74 |
| **hsa-miR-187** | UCGUGUCUUGUGUUGCAGCCGG | 16.15 | ± 2.93 | 23.11 | ± 3.33 |
| **hsa-miR-188-3p** | CUCCCACAUGCAGGGUUUGCA | 31.70 | ± 4.24 | 30.60 | ± 3.97 |
| **hsa-miR-188-5p** | CAUCCCUUGCAUGGUGGAGGG | 27.12 | ± 0.84 | 30.18 | ± 5.42 |
| **hsa-miR-190** | UGAUAUGUUUGAUAUAUUAGGU | 23.10 | ± 2.84 | 30.60 | ± 4.57 |
| **hsa-miR-190b** | UGAUAUGUUUGAUAUUGGGUU | 22.43 | ± 1.89 | 36.37 | ± 3.57 |
| **hsa-miR-191** | CAACGGAAUCCCAAAAGCAGCUG | 29.50 | ± 0.36 | 28.01 | ± 1.15 |
| **hsa-miR-192** | CUGACCUAUGAAUUGACAGCC | 32.18 | ± 4.83 | 30.76 | ± 4.09 |
| **hsa-miR-193a-3p** | AACUGGCCUACAAAGUCCCAGU | 29.25 | ± 5.44 | 31.89 | ± 3.08 |
| **hsa-miR-193a-5p** | UGGGUCUUUGCGGGCGAGAUGA | 40.00 | ± 5.46 | 31.25 | ± 1.49 |
| **hsa-miR-193b** | AACUGGCCCUCAAAGUCCCGCU | 29.12 | ± 1.58 | 31.49 | ± 2.25 |
| **hsa-miR-194** | UGUAACAGCAACUCCAUGUGGA | 26.68 | ± 1.77 | 28.58 | ± 2.68 |
| **hsa-miR-195** | UAGCAGCACAGAAAUAUUGGC | 25.30 | ± 4.10 | 22.56 | ± 1.34 |
| **hsa-miR-196a** | UAGGUAGUUUCAUGUUGUUGGG | 35.39 | ± 3.99 | 31.10 | ± 3.45 |
| **hsa-miR-196b** | UAGGUAGUUUCCUGUUGUUGGG | 30.88 | ± 1.66 | 31.07 | ± 1.45 |
| **hsa-miR-197** | UUCACCACCUUCUCCACCCAGC | 25.93 | ± 2.45 | 28.79 | ± 5.00 |
| **hsa-miR-198** | GGUCCAGAGGGGAGAUAGGUUC | 40.00 | ± 5.54 | 33.60 | ± 4.09 |
| **hsa-miR-199a-3p** | ACAGUAGUCUGCACAUUGGUUA | 26.59 | ± 4.46 | 28.77 | ± 2.12 |
| **hsa-miR-199a-5p** | CCCAGUGUUCAGACUACCUGUUC | 28.61 | ± 1.66 | 29.51 | ± 1.66 |
| **hsa-miR-199b-3p** | ACAGUAGUCUGCACAUUGGUUA | 25.53 | ± 1.77 | 29.03 | ± 1.72 |
| **hsa-miR-199b-5p** | CCCAGUGUUUAGACUAUCUGUUC | 27.36 | ± 3.10 | 31.36 | ± 2.88 |
| **hsa-miR-200a** | UAACACUGUCUGGUAACGAUGU | 30.63 | ± 3.89 | 36.23 | ± 4.15 |
| **hsa-miR-200b** | UAAUACUGCCUGGUAAUGAUGA | 26.59 | ± 1.69 | 30.78 | ± 5.89 |
| **hsa-miR-200c** | UAAUACUGCCGGGUAAUGAUGGA | 30.40 | ± 1.85 | 32.70 | ± 2.25 |
| **hsa-miR-202** | AGAGGUAUAGGGCAUGGGAA | 29.87 | ± 4.98 | 30.18 | ± 2.84 |
| **hsa-miR-203** | GUGAAAUGUUUAGGACCACUAG | 32.35 | ± 3.25 | 35.59 | ± 4.97 |
| **hsa-miR-204** | UUCCCUUUGUCAUCCUAUGCCU | 26.64 | ± 2.25 | 34.78 | ± 4.74 |
| **hsa-miR-205** | UCCUUCAUUCCACCGGAGUCUG | 31.34 | ± 1.53 | 33.31 | ± 6.00 |
| **hsa-miR-206** | UGGAAUGUAAGGAAGUGUGUGG | 29.09 | ± 4.23 | 31.38 | ± 5.02 |
| **hsa-miR-208a** | AUAAGACGAGCAAAAAGCUUGU | 30.47 | ± 0.65 | 32.35 | ± 3.63 |
| **hsa-miR-208b** | AUAAGACGAACAAAAGGUUUGU | 35.59 | ± 0.69 | 26.64 | ± 0.65 |
| **hsa-miR-210** | CUGUGCGUGUGACAGCGGCUGA | 33.00 | ± 0.12 | 31.10 | ± 0.30 |
| **hsa-miR-211** | UUCCCUUUGUCAUCCUUCGCCU | 27.23 | ± 0.99 | 32.37 | ± 3.44 |
| **hsa-miR-212** | UAACAGUCUCCAGUCACGGCC | 24.31 | ± 1.19 | 24.95 | ± 1.38 |
| **hsa-miR-214** | ACAGCAGGCACAGACAGGCAGU | 30.43 | ± 1.01 | 31.00 | ± 0.43 |
| **hsa-miR-215** | AUGACCUAUGAAUUGACAGAC | 35.59 | ± 1.13 | 22.65 | ± 0.44 |
| **hsa-miR-216a** | UAAUCUCAGCUGGCAACUGUGA | 32.65 | ± 1.21 | 32.17 | ± 2.58 |
| **hsa-miR-216b** | AAAUCUCUGCAGGCAAAUGUGA | 29.36 | ± 1.27 | 30.63 | ± 5.37 |
| **hsa-miR-217** | UACUGCAUCAGGAACUGAUUGGA | 40.00 | ± 1.46 | 33.07 | ± 5.17 |
| **hsa-miR-218** | UUGUGCUUGAUCUAACCAUGU | 34.78 | ± 1.45 | 32.67 | ± 2.58 |
| **hsa-miR-219-1-3p** | AGAGUUGAGUCUGGACGUCCCG | 20.13 | ± 6.32 | 25.17 | ± 3.41 |
| **hsa-miR-219-2-3p** | AGAAUUGUGGCUGGACAUCUGU | 35.38 | ± 5.53 | 29.28 | ± 2.41 |
| **hsa-miR-219-5p** | UGAUUGUCCAAACGCAAUUCU | 28.99 | ± 5.64 | 26.07 | ± 3.43 |
| **hsa-miR-220a** | CCACACCGUAUCUGACACUUU | 27.15 | ± 5.02 | 33.08 | ± 4.47 |
| **hsa-miR-220b** | CCACCACCGUGUCUGACACUU | 30.25 | ± 3.42 | 33.80 | ± 5.38 |
| **hsa-miR-220c** | ACACAGGGCUGUUGUGAAGACU | 27.92 | ± 3.49 | 36.84 | ± 2.58 |
| **hsa-miR-221** | AGCUACAUUGUCUGCUGGGUUUC | 28.20 | ± 1.44 | 28.61 | ± 2.56 |
| **hsa-miR-222** | AGCUACAUCUGGCUACUGGGU | 29.04 | ± 1.56 | 31.82 | ± 6.67 |
| **hsa-miR-223** | UGUCAGUUUGUCAAAUACCCCA | 23.68 | ± 5.53 | 23.63 | ± 3.44 |
| **hsa-miR-224** | CAAGUCACUAGUGGUUCCGUU | 33.08 | ± 4.53 | 30.63 | ± 1.44 |
| **hsa-miR-296-3p** | GAGGGUUGGGUGGAGGCUCUCC | 30.22 | ± 0.23 | 32.66 | ± 4.20 |
| **hsa-miR-296-5p** | AGGGCCCCCCCUCAAUCCUGU | 30.95 | ± 0.56 | 32.56 | ± 3.42 |
| **hsa-miR-297** | AUGUAUGUGUGCAUGUGCAUG | 28.90 | ± 0.93 | 29.36 | ± 4.54 |
| **hsa-miR-298** | AGCAGAAGCAGGGAGGUUCUCCCA | 28.42 | ± 0.95 | 32.40 | ± 4.51 |
| **hsa-miR-299-3p** | UAUGUGGGAUGGUAAACCGCUU | 30.74 | ± 0.78 | 29.01 | ± 3.44 |
| **hsa-miR-299-5p** | UGGUUUACCGUCCCACAUACAU | 23.63 | ± 0.89 | 32.54 | ± 2.73 |
| **hsa-miR-300** | UAUACAAGGGCAGACUCUCUCU | 31.66 | ± 4.22 | 30.83 | ± 3.12 |
| **hsa-miR-301a** | CAGUGCAAUAGUAUUGUCAAAGC | 23.04 | ± 3.22 | 27.76 | ± 0.96 |
| **hsa-miR-301b** | CAGUGCAAUGAUAUUGUCAAAGC | 27.42 | ± 3.48 | 28.44 | ± 2.31 |
| **hsa-miR-302a** | UAAGUGCUUCCAUGUUUUGGUGA | 27.69 | ± 3.55 | 31.53 | ± 1.94 |
| **hsa-miR-302b** | UAAGUGCUUCCAUGUUUUAGUAG | 29.36 | ± 3.61 | 34.10 | ± 2.31 |
| **hsa-miR-302c** | UAAGUGCUUCCAUGUUUCAGUGG | 27.34 | ± 1.43 | 31.85 | ± 3.71 |
| **hsa-miR-302d** | UAAGUGCUUCCAUGUUUGAGUGU | 29.11 | ± 1.53 | 32.59 | ± 4.43 |
| **hsa-miR-302e** | UAAGUGCUUCCAUGCUU | 27.76 | ± 1.48 | 28.69 | ± 3.48 |
| **hsa-miR-302f** | UAAUUGCUUCCAUGUUU | 28.44 | ± 1.43 | 15.32 | ± 5.95 |
| **hsa-miR-320a** | AAAAGCUGGGUUGAGAGGGCGA | 24.86 | ± 2.12 | 24.42 | ± 4.71 |
| **hsa-miR-320b** | AAAAGCUGGGUUGAGAGGGCAA | 25.11 | ± 2.09 | 26.55 | ± 3.95 |
| **hsa-miR-320c** | AAAAGCUGGGUUGAGAGGGU | 31.94 | ± 7.73 | 25.18 | ± 3.79 |
| **hsa-miR-320d** | AAAAGCUGGGUUGAGAGGA | 27.64 | ± 0.96 | 22.39 | ± 4.99 |
| **hsa-miR-323-3p** | CACAUUACACGGUCGACCUCU | 29.67 | ± 1.02 | 28.59 | ± 2.31 |
| **hsa-miR-323-5p** | AGGUGGUCCGUGGCGCGUUCGC | 27.37 | ± 1.68 | 27.95 | ± 2.62 |
| **hsa-miR-324-3p** | ACUGCCCCAGGUGCUGCUGG | 40.00 | ± 2.34 | 7.68 | ± 8.39 |
| **hsa-miR-324-5p** | CGCAUCCCCUAGGGCAUUGGUGU | 28.12 | ± 4.23 | 30.75 | ± 5.55 |
| **hsa-miR-325** | CCUAGUAGGUGUCCAGUAAGUGU | 30.75 | ± 4.22 | 29.60 | ± 7.99 |
| **hsa-miR-326** | CCUCUGGGCCCUUCCUCCAG | 26.26 | ± 1.43 | 30.34 | ± 3.18 |
| **hsa-miR-328** | CUGGCCCUCUCUGCCCUUCCGU | 31.32 | ± 4.61 | 30.48 | ± 2.70 |
| **hsa-miR-329** | AACACACCUGGUUAACCUCUUU | 27.26 | ± 4.69 | 27.26 | ± 1.88 |
| **hsa-miR-330-3p** | GCAAAGCACACGGCCUGCAGAGA | 29.32 | ± 1.51 | 32.83 | ± 6.07 |
| **hsa-miR-330-5p** | UCUCUGGGCCUGUGUCUUAGGC | 30.01 | ± 1.51 | 12.05 | ± 3.18 |
| **hsa-miR-331-3p** | GCCCCUGGGCCUAUCCUAGAA | 27.80 | ± 1.51 | 29.93 | ± 6.38 |
| **hsa-miR-331-5p** | CUAGGUAUGGUCCCAGGGAUCC | 40.00 | ± 2.56 | 15.96 | ± 19.61 |
| **hsa-miR-335** | UCAAGAGCAAUAACGAAAAAUGU | 29.60 | ± 2.88 | 29.66 | ± 2.64 |
| **hsa-miR-337-3p** | CUCCUAUAUGAUGCCUUUCUUC | 27.37 | ± 2.81 | 30.14 | ± 2.74 |
| **hsa-miR-337-5p** | GAACGGCUUCAUACAGGAGUU | 31.52 | ± 2.56 | 32.45 | ± 2.32 |
| **hsa-miR-338-3p** | UCCAGCAUCAGUGAUUUUGUUG | 26.63 | ± 0.64 | 30.70 | ± 4.38 |
| **hsa-miR-338-5p** | AACAAUAUCCUGGUGCUGAGUG | 25.97 | ± 0.78 | 30.00 | ± 4.20 |
| **hsa-miR-339-3p** | UGAGCGCCUCGACGACAGAGCCG | 26.90 | ± 4.21 | 30.01 | ± 2.64 |
| **hsa-miR-339-5p** | UCCCUGUCCUCCAGGAGCUCACG | 29.34 | ± 2.78 | 31.26 | ± 0.57 |
| **hsa-miR-340** | UUAUAAAGCAAUGAGACUGAUU | 28.59 | ± 2.88 | 19.21 | ± 1.10 |
| **hsa-miR-342-3p** | UCUCACACAGAAAUCGCACCCGU | 28.84 | ± 2.91 | 31.52 | ± 4.20 |
| **hsa-miR-342-5p** | AGGGGUGCUAUCUGUGAUUGA | 28.44 | ± 2.92 | 27.42 | ± 1.08 |
| **hsa-miR-345** | GCUGACUCCUAGUCCAGGGCUC | 33.30 | ± 5.81 | 30.74 | ± 3.27 |
| **hsa-miR-346** | UGUCUGCCCGCAUGCCUGCCUCU | 28.05 | ± 3.09 | 30.56 | ± 4.43 |
| **hsa-miR-361-3p** | UCCCCCAGGUGUGAUUCUGAUUU | 31.52 | ± 3.12 | 20.04 | ± 5.01 |
| **hsa-miR-361-5p** | UUAUCAGAAUCUCCAGGGGUAC | 27.42 | ± 3.33 | 31.35 | ± 4.02 |
| **hsa-miR-362-3p** | AACACACCUAUUCAAGGAUUCA | 30.69 | ± 0.67 | 37.67 | ± 3.02 |
| **hsa-miR-362-5p** | AAUCCUUGGAACCUAGGUGUGAGU | 20.94 | ± 1.66 | 21.91 | ± 2.48 |
| **hsa-miR-363** | AAUUGCACGGUAUCCAUCUGUA | 23.37 | ± 1.78 | 30.52 | ± 4.10 |
| **hsa-miR-365** | UAAUGCCCCUAAAAAUCCUUAU | 30.83 | ± 1.77 | 23.37 | ± 5.47 |
| **hsa-miR-367** | AAUUGCACUUUAGCAAUGGUGA | 27.24 | ± 3.28 | 30.83 | ± 3.01 |
| **hsa-miR-369-3p** | AAUAAUACAUGGUUGAUCUUU | 33.31 | ± 3.30 | 22.50 | ± 8.67 |
| **hsa-miR-369-5p** | AGAUCGACCGUGUUAUAUUCGC | 30.13 | ± 3.28 | 32.15 | ± 8.36 |
| **hsa-miR-370** | GCCUGCUGGGGUGGAACCUGGU | 25.15 | ± 3.74 | 32.03 | ± 6.84 |
| **hsa-miR-371-3p** | AAGUGCCGCCAUCUUUUGAGUGU | 20.94 | ± 3.71 | 24.38 | ± 3.53 |
| **hsa-miR-371-5p** | ACUCAAACUGUGGGGGCACU | 22.91 | ± 3.12 | 23.29 | ± 1.65 |
| **hsa-miR-372** | AAAGUGCUGCGACAUUUGAGCGU | 29.01 | ± 1.02 | 34.40 | ± 4.02 |
| **hsa-miR-373** | GAAGUGCUUCGAUUUUGGGGUGU | 25.37 | ± 5.41 | 31.97 | ± 4.05 |
| **hsa-miR-374a** | UUAUAAUACAACCUGAUAAGUG | 30.52 | ± 3.41 | 33.31 | ± 1.08 |
| **hsa-miR-374b** | AUAUAAUACAACCUGCUAAGUG | 23.37 | ± 5.04 | 30.13 | ± 0.39 |
| **hsa-miR-375** | UUUGUUCGUUCGGCUCGCGUGA | 25.64 | ± 2.25 | 26.99 | ± 3.52 |
| **hsa-miR-376a** | AUCAUAGAGGAAAAUCCACGU | 31.65 | ± 2.22 | 34.31 | ± 2.48 |
| **hsa-miR-376b** | AUCAUAGAGGAAAAUCCAUGUU | 40.00 | ± 2.63 | 34.70 | ± 4.10 |
| **hsa-miR-376c** | AACAUAGAGGAAAUUCCACGU | 26.75 | ± 2.25 | 28.78 | ± 3.19 |
| **hsa-miR-377** | AUCACACAAAGGCAACUUUUGU | 27.68 | ± 3.43 | 28.79 | ± 2.99 |
| **hsa-miR-378** | ACUGGACUUGGAGUCAGAAGG | 26.36 | ± 2.39 | 29.47 | ± 2.56 |
| **hsa-miR-379** | UGGUAGACUAUGGAACGUAGG | 32.37 | ± 2.44 | 31.65 | ± 5.43 |
| **hsa-miR-380** | UAUGUAAUAUGGUCCACAUCUU | 27.16 | ± 2.39 | 33.68 | ± 4.51 |
| **hsa-miR-381** | UAUACAAGGGCAAGCUCUCUGU | 24.50 | ± 1.33 | 30.59 | ± 4.88 |
| **hsa-miR-382** | GAAGUUGUUCGUGGUGGAUUCG | 33.52 | ± 5.62 | 30.59 | ± 1.20 |
| **hsa-miR-383** | AGAUCAGAAGGUGAUUGUGGCU | 30.84 | ± 4.24 | 32.37 | ± 2.32 |
| **hsa-miR-384** | AUUCCUAGAAAUUGUUCAUA | 22.67 | ± 4.34 | 27.16 | ± 1.36 |
| **hsa-miR-409-3p** | GAAUGUUGCUCGGUGAACCCCU | 30.67 | ± 1.58 | 33.23 | ± 4.88 |
| **hsa-miR-409-5p** | AGGUUACCCGAGCAACUUUGCAU | 27.16 | ± 1.79 | 33.64 | ± 5.15 |
| **hsa-miR-410** | AAUAUAACACAGAUGGCCUGU | 5.59 | ± 1.69 | 22.67 | ± 3.11 |
| **hsa-miR-411** | UAGUAGACCGUAUAGCGUACG | 30.65 | ± 1.68 | 19.81 | ± 4.11 |
| **hsa-miR-412** | ACUUCACCUGGUCCACUAGCCGU | 27.00 | ± 1.18 | 32.52 | ± 5.52 |
| **hsa-miR-421** | AUCAACAGACAUUAAUUGGGCGC | 18.35 | ± 3.05 | 20.71 | ± 4.49 |
| **hsa-miR-422a** | ACUGGACUUAGGGUCAGAAGGC | 29.85 | ± 4.76 | 30.71 | ± 5.42 |
| **hsa-miR-423-3p** | AGCUCGGUCUGAGGCCCCUCAGU | 28.03 | ± 1.79 | 30.65 | ± 2.86 |
| **hsa-miR-423-5p** | UGAGGGGCAGAGAGCGAGACUUU | 27.82 | ± 0.77 | 29.92 | ± 0.72 |
| **hsa-miR-424** | CAGCAGCAAUUCAUGUUUUGAA | 26.19 | ± 0.59 | 29.61 | ± 0.52 |
| **hsa-miR-425** | AAUGACACGAUCACUCCCGUUGA | 20.68 | ± 3.24 | 28.11 | ± 4.76 |
| **hsa-miR-429** | UAAUACUGUCUGGUAAAACCGU | 30.42 | ± 3.21 | 32.50 | ± 2.81 |
| **hsa-miR-431** | UGUCUUGCAGGCCGUCAUGCA | 32.67 | ± 6.54 | 30.64 | ± 5.27 |
| **hsa-miR-432** | UCUUGGAGUAGGUCAUUGGGUGG | 28.30 | ± 2.05 | 32.26 | ± 3.11 |
| **hsa-miR-433** | AUCAUGAUGGGCUCCUCGGUGU | 27.26 | ± 3.26 | 29.47 | ± 3.64 |
| **hsa-miR-448** | UUGCAUAUGUAGGAUGUCCCAU | 29.47 | ± 3.28 | 25.58 | ± 1.68 |
| **hsa-miR-449a** | UGGCAGUGUAUUGUUAGCUGGU | 27.64 | ± 2.64 | 22.98 | ± 1.18 |
| **hsa-miR-449b** | AGGCAGUGUAUUGUUAGCUGGC | 26.83 | ± 1.92 | 28.97 | ± 3.05 |
| **hsa-miR-450a** | UUUUGCGAUGUGUUCCUAAUAU | 29.92 | ± 1.26 | 22.98 | ± 2.33 |
| **hsa-miR-450b-3p** | UUGGGAUCAUUUUGCAUCCAUA | 29.61 | ± 1.58 | 21.40 | ± 6.10 |
| **hsa-miR-450b-5p** | UUUUGCAAUAUGUUCCUGAAUA | 28.11 | ± 2.33 | 38.50 | ± 5.61 |
| **hsa-miR-451** | AAACCGUUACCAUUACUGAGUU | 18.65 | ± 5.05 | 22.01 | ± 0.50 |
| **hsa-miR-452** | AACUGUUUGCAGAGGAAACUGA | 30.23 | ± 0.78 | 33.22 | ± 3.30 |
| **hsa-miR-453** | AGGUUGUCCGUGGUGAGUUCGCA | 31.59 | ± 4.80 | 29.64 | ± 5.92 |
| **hsa-miR-454** | UAGUGCAAUAUUGCUUAUAGGGU | 31.01 | ± 4.77 | 30.37 | ± 3.84 |
| **hsa-miR-455-3p** | GCAGUCCAUGGGCAUAUACAC | 28.98 | ± 2.11 | 33.31 | ± 4.64 |
| **hsa-miR-455-5p** | UAUGUGCCUUUGGACUACAUCG | 33.22 | ± 2.45 | 34.12 | ± 5.27 |
| **hsa-miR-483-3p** | UCACUCCUCUCCUCCCGUCUU | 26.18 | ± 2.11 | 29.10 | ± 4.38 |
| **hsa-miR-483-5p** | AAGACGGGAGGAAAGAAGGGAG | 27.47 | ± 0.77 | 30.86 | ± 1.51 |
| **hsa-miR-484** | UCAGGCUCAGUCCCCUCCCGAU | 30.86 | ± 0.78 | 29.10 | ± 4.44 |
| **hsa-miR-485-3p** | GUCAUACACGGCUCUCCUCUCU | 27.58 | ± 2.22 | 30.21 | ± 1.46 |
| **hsa-miR-485-5p** | AGAGGCUGGCCGUGAUGAAUUC | 30.83 | ± 2.35 | 35.67 | ± 1.50 |
| **hsa-miR-486-3p** | CGGGGCAGCUCAGUACAGGAU | 29.31 | ± 2.66 | 31.79 | ± 5.16 |
| **hsa-miR-486-5p** | UCCUGUACUGAGCUGCCCCGAG | 24.26 | ± 1.90 | 25.16 | ± 2.63 |
| **hsa-miR-487a** | AAUCAUACAGGGACAUCCAGUU | 30.74 | ± 1.86 | 25.25 | ± 0.71 |
| **hsa-miR-487b** | AAUCGUACAGGGUCAUCCACUU | 27.99 | ± 1.59 | 33.73 | ± 2.36 |
| **hsa-miR-488** | UUGAAAGGCUAUUUCUUGGUC | 24.12 | ± 1.40 | 33.00 | ± 5.34 |
| **hsa-miR-489** | GUGACAUCACAUAUACGGCAGC | 28.35 | ± 3.12 | 30.22 | ± 2.14 |
| **hsa-miR-490-3p** | CAACCUGGAGGACUCCAUGCUG | 31.26 | ± 4.59 | 34.45 | ± 3.14 |
| **hsa-miR-490-5p** | CCAUGGAUCUCCAGGUGGGU | 26.77 | ± 2.32 | 28.77 | ± 3.88 |
| **hsa-miR-491-3p** | CUUAUGCAAGAUUCCCUUCUAC | 26.77 | ± 2.38 | 28.48 | ± 3.98 |
| **hsa-miR-491-5p** | AGUGGGGAACCCUUCCAUGAGG | 28.92 | ± 1.06 | 20.41 | ± 4.34 |
| **hsa-miR-492** | AGGACCUGCGGGACAAGAUUCUU | 28.62 | ± 1.09 | 24.68 | ± 4.79 |
| **hsa-miR-493** | UGAAGGUCUACUGUGUGCCAGG | 25.07 | ± 1.45 | 30.74 | ± 2.39 |
| **hsa-miR-494** | UGAAACAUACACGGGAAACCUC | 29.42 | ± 2.36 | 27.99 | ± 2.46 |
| **hsa-miR-495** | AAACAAACAUGGUGCACUUCUU | 30.76 | ± 2.77 | 31.09 | ± 4.66 |
| **hsa-miR-496** | UGAGUAUUACAUGGCCAAUCUC | 31.49 | ± 2.65 | 31.49 | ± 2.66 |
| **hsa-miR-497** | CAGCAGCACACUGUGGUUUGU | 27.63 | ± 2.45 | 31.93 | ± 0.68 |
| **hsa-miR-498** | UUUCAAGCCAGGGGGCGUUUUUC | 28.77 | ± 2.36 | 34.00 | ± 4.28 |
| **hsa-miR-499-3p** | AACAUCACAGCAAGUCUGUGCU | 29.02 | ± 6.74 | 21.37 | ± 2.38 |
| **hsa-miR-499-5p** | UUAAGACUUGCAGUGAUGUUU | 29.32 | ± 1.74 | 29.28 | ± 1.06 |
| **hsa-miR-500** | UAAUCCUUGCUACCUGGGUGAGA | 31.66 | ± 1.77 | 32.02 | ± 1.44 |
| **hsa-miR-501-3p** | AAUGCACCCGGGCAAGGAUUCU | 29.56 | ± 1.80 | 30.76 | ± 4.60 |
| **hsa-miR-501-5p** | AAUCCUUUGUCCCUGGGUGAGA | 27.37 | ± 4.65 | 31.49 | ± 0.17 |
| **hsa-miR-502-3p** | AAUGCACCUGGGCAAGGAUUCA | 29.58 | ± 3.65 | 27.99 | ± 0.25 |
| **hsa-miR-502-5p** | AUCCUUGCUAUCUGGGUGCUA | 26.95 | ± 1.77 | 28.35 | ± 2.11 |
| **hsa-miR-503** | UAGCAGCGGGAACAGUUCUGCAG | 31.92 | ± 1.89 | 28.17 | ± 4.06 |
| **hsa-miR-504** | AGACCCUGGUCUGCACUCUAUC | 27.37 | ± 1.46 | 25.41 | ± 4.88 |
| **hsa-miR-505** | CGUCAACACUUGCUGGUUUCCU | 26.75 | ± 1.85 | 31.92 | ± 2.12 |
| **hsa-miR-506** | UAAGGCACCCUUCUGAGUAGA | 26.96 | ± 1.77 | 27.37 | ± 4.60 |
| **hsa-miR-507** | UUUUGCACCUUUUGGAGUGAA | 37.41 | ± 1.80 | 34.63 | ± 0.17 |
| **hsa-miR-508-3p** | UGAUUGUAGCCUUUUGGAGUAGA | 28.98 | ± 4.65 | 25.97 | ± 0.25 |
| **hsa-miR-508-5p** | UACUCCAGAGGGCGUCACUCAUG | 31.49 | ± 1.36 | 31.31 | ± 4.15 |
| **hsa-miR-509-3-5p** | UACUGCAGACGUGGCAAUCAUG | 31.02 | ± 1.45 | 32.68 | ± 1.52 |
| **hsa-miR-509-3p** | UGAUUGGUACGUCUGUGGGUAG | 28.96 | ± 2.36 | 25.64 | ± 1.66 |
| **hsa-miR-509-5p** | UACUGCAGACAGUGGCAAUCA | 32.17 | ± 2.77 | 24.70 | ± 1.78 |
| **hsa-miR-510** | UACUCAGGAGAGUGGCAAUCAC | 34.29 | ± 2.65 | 33.05 | ± 1.89 |
| **hsa-miR-511** | GUGUCUUUUGCUCUGCAGUCA | 35.07 | ± 2.45 | 37.41 | ± 1.59 |
| **hsa-miR-512-3p** | AAGUGCUGUCAUAGCUGAGGUC | 29.10 | ± 2.36 | 28.98 | ± 2.59 |
| **hsa-miR-512-5p** | CACUCAGCCUUGAGGGCACUUUC | 27.88 | ± 0.93 | 31.90 | ± 3.95 |
| **hsa-miR-513a-3p** | UAAAUUUCACCUUUCUGAGAAGG | 33.22 | ± 2.39 | 35.18 | ± 1.45 |
| **hsa-miR-513a-5p** | UUCACAGGGAGGUGUCAU | 29.64 | ± 2.44 | 36.11 | ± 1.56 |
| **hsa-miR-513b** | UUCACAAGGAGGUGUCAUUUAU | 30.37 | ± 2.39 | 12.93 | ± 1.58 |
| **hsa-miR-513c** | UUCUCAAGGAGGUGUCGUUUAU | 33.31 | ± 1.33 | 33.53 | ± 4.88 |
| **hsa-miR-514** | AUUGACACUUCUGUGAGUAGA | 34.12 | ± 5.62 | 23.80 | ± 5.88 |
| **hsa-miR-515-3p** | GAGUGCCUUCUUUUGGAGCGUU | 29.10 | ± 4.24 | 34.77 | ± 4.08 |
| **hsa-miR-515-5p** | UUCUCCAAAAGAAAGCACUUUCUG | 30.86 | ± 4.34 | 34.29 | ± 4.22 |
| **hsa-miR-516a-3p** | UGCUUCCUUUCAGAGGGU | 29.49 | ± 1.58 | 35.07 | ± 3.22 |
| **hsa-miR-516a-5p** | UUCUCGAGGAAAGAAGCACUUUC | 27.77 | ± 1.79 | 23.29 | ± 2.22 |
| **hsa-miR-516b** | AUCUGGAGGUAAGAAGCACUUU | 28.99 | ± 1.69 | 28.91 | ± 4.20 |
| **hsa-miR-517a** | AUCGUGCAUCCCUUUAGAGUGU | 29.13 | ± 1.68 | 32.66 | ± 6.97 |
| **hsa-miR-517b** | UCGUGCAUCCCUUUAGAGUGUU | 30.02 | ± 1.18 | 31.03 | ± 1.67 |
| **hsa-miR-517c** | AUCGUGCAUCCUUUUAGAGUGU | 29.73 | ± 3.05 | 34.35 | ± 4.93 |
| **hsa-miR-518a-3p** | GAAAGCGCUUCCCUUUGCUGGA | 27.55 | ± 4.76 | 31.76 | ± 3.00 |
| **hsa-miR-518a-5p** | CUGCAAAGGGAAGCCCUUUC | 31.39 | ± 1.79 | 32.56 | ± 4.89 |
| **hsa-miR-518b** | CAAAGCGCUCCCCUUUAGAGGU | 31.48 | ± 2.48 | 31.05 | ± 1.69 |
| **hsa-miR-518c** | CAAAGCGCUUCUCUUUAGAGUGU | 30.48 | ± 6.48 | 35.41 | ± 1.68 |
| **hsa-miR-518d-3p** | CAAAGCGCUUCCCUUUGGAGC | 27.48 | ± 4.20 | 35.10 | ± 3.38 |
| **hsa-miR-518d-5p** | CUCUAGAGGGAAGCACUUUCUG | 28.48 | ± 6.97 | 32.49 | ± 3.68 |
| **hsa-miR-518e** | AAAGCGCUUCCCUUCAGAGUG | 31.52 | ± 1.67 | 34.77 | ± 3.63 |
| **hsa-miR-518f** | GAAAGCGCUUCUCUUUAGAGG | 37.41 | ± 1.69 | 31.89 | ± 4.15 |
| **hsa-miR-519a** | AAAGUGCAUCCUUUUAGAGUGU | 28.98 | ± 1.46 | 37.98 | ± 4.05 |
| **hsa-miR-519b-3p** | AAAGUGCAUCCUUUUAGAGGUU | 31.90 | ± 1.56 | 38.97 | ± 4.18 |
| **hsa-miR-519b-5p** | CUCUAGAGGGAAGCGCUUUCUG | 30.92 | ± 0.62 | 32.66 | ± 4.74 |
| **hsa-miR-519c-3p** | AAAGUGCAUCUUUUUAGAGGAU | 32.49 | ± 0.78 | 36.21 | ± 2.74 |
| **hsa-miR-519c-5p** | CUCUAGAGGGAAGCGCUUUCUG | 28.41 | ± 0.66 | 33.19 | ± 1.55 |
| **hsa-miR-519d** | CAAAGUGCCUCCCUUUAGAGUG | 30.18 | ± 2.00 | 34.97 | ± 1.67 |
| **hsa-miR-519e** | AAGUGCCUCCUUUUAGAGUGUU | 33.34 | ± 2.08 | 32.32 | ± 2.15 |
| **hsa-miR-520a-3p** | AAAGUGCUUCCCUUUGGACUGU | 29.02 | ± 1.34 | 33.13 | ± 1.55 |
| **hsa-miR-520a-5p** | CUCCAGAGGGAAGUACUUUCU | 35.67 | ± 1.38 | 33.13 | ± 1.55 |
| **hsa-miR-520b** | AAAGUGCUUCCUUUUAGAGGG | 31.79 | ± 1.45 | 34.78 | ± 4.87 |
| **hsa-miR-520c-3p** | AAAGUGCUUCCUUUUAGAGGGU | 25.16 | ± 1.48 | 30.19 | ± 4.87 |
| **hsa-miR-520c-5p** | CUCUAGAGGGAAGCACUUUCUG | 29.81 | ± 0.66 | 31.30 | ± 7.05 |
| **hsa-miR-520d-3p** | AAAGUGCUUCUCUUUGGUGGGU | 28.55 | ± 0.94 | 28.63 | ± 1.16 |
| **hsa-miR-520d-5p** | CUACAAAGGGAAGCCCUUUC | 32.66 | ± 0.94 | 33.61 | ± 4.47 |
| **hsa-miR-520e** | AAAGUGCUUCCUUUUUGAGGG | 30.66 | ± 0.38 | 33.34 | ± 5.84 |
| **hsa-miR-520f** | AAGUGCUUCCUUUUAGAGGGUU | 30.76 | ± 0.47 | 36.32 | ± 1.55 |
| **hsa-miR-520g** | ACAAAGUGCUUCCCUUUAGAGUGU | 29.99 | ± 2.51 | 26.56 | ± 4.87 |
| **hsa-miR-520h** | ACAAAGUGCUUCCCUUUAGAGU | 29.87 | ± 3.25 | 32.87 | ± 3.47 |
| **hsa-miR-521** | AACGCACUUCCCUUUAGAGUGU | 31.04 | ± 0.66 | 33.94 | ± 1.16 |
| **hsa-miR-522** | AAAAUGGUUCCCUUUAGAGUGU | 26.56 | ± 3.56 | 34.79 | ± 4.47 |
| **hsa-miR-523** | GAACGCGCUUCCCUAUAGAGGGU | 28.28 | ± 1.28 | 29.62 | ± 0.88 |
| **hsa-miR-524-3p** | GAAGGCGCUUCCCUUUGGAGU | 30.61 | ± 0.75 | 35.06 | ± 4.58 |
| **hsa-miR-524-5p** | CUACAAAGGGAAGCACUUUCUC | 28.61 | ± 2.47 | 34.85 | ± 0.62 |
| **hsa-miR-525-3p** | GAAGGCGCUUCCCUUUAGAGCG | 29.38 | ± 1.46 | 33.90 | ± 0.78 |
| **hsa-miR-525-5p** | CUCCAGAGGGAUGCACUUUCU | 29.44 | ± 0.66 | 33.71 | ± 6.82 |
| **hsa-miR-526a** | CUCUAGAGGGAAGCACUUUCUG | 40.00 | ± 0.99 | 32.22 | ± 4.56 |
| **hsa-miR-526b** | CUCUUGAGGGAAGCACUUUCUGU | 28.00 | ± 0.80 | 31.84 | ± 1.70 |
| **hsa-miR-527** | CUGCAAAGGGAAGCCCUUUC | 34.26 | ± 4.12 | 33.22 | ± 7.80 |
| **hsa-miR-532-3p** | CCUCCCACACCCAAGGCUUGCA | 27.65 | ± 1.70 | 29.75 | ± 0.87 |
| **hsa-miR-532-5p** | CAUGCCUUGAGUGUAGGACCGU | 27.84 | ± 1.75 | 32.99 | ± 5.26 |
| **hsa-miR-539** | GGAGAAAUUAUCCUUGGUGUGU | 30.90 | ± 5.88 | 29.50 | ± 2.86 |
| **hsa-miR-541** | UGGUGGGCACAGAAUCUGGACU | 29.69 | ± 4.88 | 22.57 | ± 21.89 |
| **hsa-miR-542-3p** | UGUGACAGAUUGAUAACUGAAA | 29.50 | ± 3.88 | 25.84 | ± 28.59 |
| **hsa-miR-542-5p** | UCGGGGAUCAUCAUGUCACGAGA | 30.09 | ± 0.87 | 30.32 | ± 2.40 |
| **hsa-miR-543** | AAACAUUCGCGGUGCACUUCUU | 30.27 | ± 0.75 | 34.52 | ± 3.56 |
| **hsa-miR-544** | AUUCUGCAUUUUUAGCAAGUUC | 29.55 | ± 0.81 | 34.36 | ± 1.28 |
| **hsa-miR-545** | UCAGCAAACAUUUAUUGUGUGC | 40.00 | ± 0.75 | 29.11 | ± 0.75 |
| **hsa-miR-548a-3p** | CAAAACUGGCAAUUACUUUUGC | 21.89 | ± 5.61 | 11.88 | ± 2.47 |
| **hsa-miR-548a-5p** | AAAAGUAAUUGCGAGUUUUACC | 28.59 | ± 4.42 | 32.92 | ± 1.46 |
| **hsa-miR-548b-3p** | CAAGAACCUCAGUUGCUUUUGU | 35.52 | ± 5.81 | 35.52 | ± 28.73 |
| **hsa-miR-548b-5p** | AAAAGUAAUUGUGGUUUUGGCC | 23.55 | ± 2.01 | 14.11 | ± 6.80 |
| **hsa-miR-548c-3p** | CAAAAAUCUCAAUUACUUUUGC | 32.92 | ± 2.08 | 35.71 | ± 21.89 |
| **hsa-miR-548c-5p** | AAAAGUAAUUGCGGUUUUUGCC | 28.73 | ± 2.64 | 29.33 | ± 2.37 |
| **hsa-miR-548d-3p** | CAAAAACCACAGUUUCUUUUGC | 31.40 | ± 2.73 | 33.44 | ± 27.65 |
| **hsa-miR-548d-5p** | AAAAGUAAUUGUGGUUUUUGCC | 29.41 | ± 1.35 | 31.52 | ± 4.64 |
| **hsa-miR-548e** | AAAAACUGAGACUACUUUUGCA | 28.25 | ± 2.23 | 33.96 | ± 2.29 |

| ***mature miRNA ID*** | ***Target microRNA Mature Sequence*** | ***Average Ct miR e-sEV*** | | ***Average Ct miR i-sEV*** | |
| --- | --- | --- | --- | --- | --- |
| **hsa-miR-323b-3p** | CCCAAUACACGGUCGACCUCUU | 28.41 | ± 6.18 | 36.70 | ± 1.62 |
| **hsa-miR-500b** | AAUCCUUGCUACCUGGGU | 29.71 | ± 2.27 | 31.57 | ± 4.43 |
| **hsa-miR-514b-5p** | UUCUCAAGAGGGAGGCAAUCAU | 27.35 | ± 2.33 | 23.47 | ± 7.47 |
| **hsa-miR-514b-3p** | AUUGACACCUCUGUGAGUGGA | 29.18 | ± 0.00 | 36.14 | ± 0.82 |
| **hsa-miR-544b** | ACCUGAGGUUGUGCAUUUCUAA | 25.01 | ± 4.07 | 22.66 | ± 2.12 |
| **hsa-miR-548f** | AAAAACUGUAAUUACUUUU | 26.84 | ± 2.00 | 31.26 | ± 2.75 |
| **hsa-miR-548g** | AAAACUGUAAUUACUUUUGUAC | 24.82 | ± 0.49 | 34.73 | ± 6.18 |
| **hsa-miR-548h** | AAAAGUAAUCGCGGUUUUUGUC | 30.82 | ± 4.16 | 30.35 | ± 2.27 |
| **hsa-miR-548i** | AAAAGUAAUUGCGGAUUUUGCC | 30.40 | ± 1.19 | 31.51 | ± 1.72 |
| **hsa-miR-548j** | AAAAGUAAUUGCGGUCUUUGGU | 32.89 | ± 6.52 | 24.37 | ± 2.56 |
| **hsa-miR-548k** | AAAAGUACUUGCGGAUUUUGCU | 30.46 | ± 1.70 | 20.04 | ± 3.66 |
| **hsa-miR-548l** | AAAAGUAUUUGCGGGUUUUGUC | 23.42 | ± 1.16 | 27.34 | ± 1.95 |
| **hsa-miR-548m** | CAAAGGUAUUUGUGGUUUUUG | 25.44 | ± 4.79 | 36.99 | ± 1.95 |
| **hsa-miR-548n** | CAAAAGUAAUUGUGGAUUUUGU | 24.79 | ± 4.35 | 36.34 | ± 3.32 |
| **hsa-miR-548o** | CCAAAACUGCAGUUACUUUUGC | 26.42 | ± 4.40 | 32.64 | ± 0.31 |
| **hsa-miR-548p** | UAGCAAAAACUGCAGUUACUUU | 30.90 | ± 3.42 | 34.04 | ± 4.79 |
| **hsa-miR-548s** | AUGGCCAAAACUGCAGUUAUUUU | 21.38 | ± 1.78 | 25.21 | ± 4.35 |
| **hsa-miR-548t** | CAAAAGUGAUCGUGGUUUUUG | 27.81 | ± 2.27 | 29.82 | ± 0.07 |
| **hsa-miR-548u** | CAAAGACUGCAAUUACUUUUGCG | 25.27 | ± 1.95 | 36.06 | ± 5.78 |
| **hsa-miR-548v** | AGCUACAGUUACUUUUGCACCA | 25.51 | ± 1.01 | 27.26 | ± 0.45 |
| **hsa-miR-548w** | AAAAGUAACUGCGGUUUUUGCCU | 28.84 | ± 1.04 | 32.54 | ± 2.26 |
| **hsa-miR-548x** | UAAAAACUGCAAUUACUUUCA | 27.63 | ± 4.35 | 26.65 | ± 3.42 |
| **hsa-miR-549** | UGACAACUAUGGAUGAGCUCU | 26.73 | ± 4.40 | 33.65 | ± 1.78 |
| **hsa-miR-550** | AGUGCCUGAGGGAGUAAGAGCCC | 29.31 | ± 9.69 | 22.91 | ± 1.19 |
| **hsa-miR-551a** | GCGACCCACUCUUGGUUUCCA | 24.71 | ± 0.11 | 31.64 | ± 0.51 |
| **hsa-miR-551b** | GCGACCCAUACUUGGUUUCAG | 29.44 | ± 5.78 | 22.93 | ± 4.88 |
| **hsa-miR-552** | AACAGGUGACUGGUUAGACAA | 29.74 | ± 1.13 | 33.04 | ± 0.42 |
| **hsa-miR-553** | AAAACGGUGAGAUUUUGUUUU | 31.90 | ± 1.49 | 30.79 | ± 6.33 |
| **hsa-miR-554** | GCUAGUCCUGACUCAGCCAGU | 28.75 | ± 3.13 | 32.56 | ± 0.32 |
| **hsa-miR-555** | AGGGUAAGCUGAACCUCUGAU | 29.63 | ± 4.66 | 32.86 | ± 0.68 |
| **hsa-miR-556-3p** | AUAUUACCAUUAGCUCAUCUUU | 31.47 | ± 1.09 | 29.54 | ± 4.31 |
| **hsa-miR-556-5p** | GAUGAGCUCAUUGUAAUAUGAG | 40.00 | ± 4.10 | 36.49 | ± 0.27 |
| **hsa-miR-557** | GUUUGCACGGGUGGGCCUUGUCU | 31.37 | ± 0.82 | 31.55 | ± 2.21 |
| **hsa-miR-558** | UGAGCUGCUGUACCAAAAU | 33.91 | ± 1.20 | 25.50 | ± 3.03 |
| **hsa-miR-559** | UAAAGUAAAUAUGCACCAAAA | 34.26 | ± 2.31 | 26.41 | ± 3.33 |
| **hsa-miR-561** | CAAAGUUUAAGAUCCUUGAAGU | 38.06 | ± 1.44 | 23.13 | ± 3.78 |
| **hsa-miR-562** | AAAGUAGCUGUACCAUUUGC | 32.04 | ± 3.03 | 29.49 | ± 4.24 |
| **hsa-miR-563** | AGGUUGACAUACGUUUCCC | 25.88 | ± 5.58 | 30.30 | ± 1.50 |
| **hsa-miR-564** | AGGCACGGUGUCAGCAGGC | 31.56 | ± 6.31 | 27.48 | ± 0.32 |
| **hsa-miR-566** | GGGCGCCUGUGAUCCCAAC | 26.48 | ± 3.83 | 29.29 | ± 1.90 |
| **hsa-miR-567** | AGUAUGUUCUUCCAGGACAGAAC | 31.90 | ± 6.33 | 26.66 | ± 0.68 |
| **hsa-miR-568** | AUGUAUAAAUGUAUACACAC | 29.45 | ± 5.68 | 39.16 | ± 1.29 |
| **hsa-miR-569** | AGUUAAUGAAUCCUGGAAAGU | 26.18 | ± 5.70 | 27.50 | ± 1.34 |
| **hsa-miR-570** | CGAAAACAGCAAUUACCUUUGC | 29.63 | ± 2.49 | 32.43 | ± 1.34 |
| **hsa-miR-571** | UGAGUUGGCCAUCUGAGUGAG | 25.14 | ± 0.68 | 20.57 | ± 1.25 |
| **hsa-miR-572** | GUCCGCUCGGCGGUGGCCCA | 31.53 | ± 7.35 | 27.49 | ± 1.56 |
| **hsa-miR-573** | CUGAAGUGAUGUGUAACUGAUCAG | 25.61 | ± 1.12 | 39.62 | ± 2.01 |
| **hsa-miR-574-3p** | CACGCUCAUGCACACACCCACA | 24.10 | ± 6.11 | 26.95 | ± 2.09 |
| **hsa-miR-574-5p** | UGAGUGUGUGUGUGUGAGUGUGU | 29.56 | ± 0.92 | 25.67 | ± 3.68 |
| **hsa-miR-575** | GAGCCAGUUGGACAGGAGC | 36.43 | ± 1.28 | 31.98 | ± 0.89 |
| **hsa-miR-576-3p** | AAGAUGUGGAAAAAUUGGAAUC | 34.60 | ± 3.21 | 31.65 | ± 1.57 |
| **hsa-miR-576-5p** | AUUCUAAUUUCUCCACGUCUUU | 35.00 | ± 0.92 | 21.48 | ± 2.68 |
| **hsa-miR-577** | UAGAUAAAAUAUUGGUACCUG | 30.99 | ± 6.13 | 28.43 | ± 2.21 |
| **hsa-miR-578** | CUUCUUGUGCUCUAGGAUUGU | 30.33 | ± 2.36 | 25.81 | ± 6.63 |
| **hsa-miR-579** | UUCAUUUGGUAUAAACCGCGAUU | 26.75 | ± 3.36 | 32.48 | ± 1.96 |
| **hsa-miR-580** | UUGAGAAUGAUGAAUCAUUAGG | 27.48 | ± 1.68 | 25.34 | ± 1.76 |
| **hsa-miR-581** | UCUUGUGUUCUCUAGAUCAGU | 25.67 | ± 1.57 | 39.00 | ± 1.00 |
| **hsa-miR-582-3p** | UAACUGGUUGAACAACUGAACC | 26.96 | ± 2.68 | 26.93 | ± 0.95 |
| **hsa-miR-582-5p** | UUACAGUUGUUCAACCAGUUACU | 30.99 | ± 0.83 | 36.13 | ± 1.81 |
| **hsa-miR-583** | CAAAGAGGAAGGUCCCAUUAC | 30.33 | ± 1.57 | 34.13 | ± 0.60 |
| **hsa-miR-584** | UUAUGGUUUGCCUGGGACUGAG | 29.55 | ± 1.58 | 29.23 | ± 0.41 |
| **hsa-miR-585** | UGGGCGUAUCUGUAUGCUA | 30.78 | ± 1.57 | 28.07 | ± 2.49 |
| **hsa-miR-586** | UAUGCAUUGUAUUUUUAGGUCC | 26.00 | ± 1.78 | 27.29 | ± 5.52 |
| **hsa-miR-587** | UUUCCAUAGGUGAUGAGUCAC | 32.84 | ± 1.69 | 21.35 | ± 0.17 |
| **hsa-miR-588** | UUGGCCACAAUGGGUUAGAAC | 29.63 | ± 0.60 | 28.49 | ± 5.63 |
| **hsa-miR-589** | UGAGAACCACGUCUGCUCUGAG | 31.47 | ± 0.41 | 30.58 | ± 1.00 |
| **hsa-miR-590-3p** | UAAUUUUAUGUAUAAGCUAGU | 30.52 | ± 2.49 | 27.15 | ± 0.95 |
| **hsa-miR-590-5p** | GAGCUUAUUCAUAAAAGUGCAG | 30.99 | ± 5.52 | 25.93 | ± 1.81 |
| **hsa-miR-591** | AGACCAUGGGUUCUCAUUGU | 29.54 | ± 0.17 | 30.45 | ± 2.36 |
| **hsa-miR-592** | UUGUGUCAAUAUGCGAUGAUGU | 24.10 | ± 5.63 | 25.58 | ± 3.36 |
| **hsa-miR-593** | UGUCUCUGCUGGGGUUUCU | 29.56 | ± 4.53 | 35.01 | ± 1.68 |
| **hsa-miR-595** | GAAGUGUGCCGUGGUGUGUCU | 36.43 | ± 3.68 | 29.77 | ± 6.17 |
| **hsa-miR-596** | AAGCCUGCCCGGCUCCUCGGG | 27.44 | ± 3.91 | 24.01 | ± 0.00 |
| **hsa-miR-597** | UGUGUCACUCGAUGACCACUGU | 24.75 | ± 0.73 | 33.61 | ± 0.13 |
| **hsa-miR-598** | UACGUCAUCGUUGUCAUCGUCA | 32.23 | ± 3.85 | 30.21 | ± 0.43 |
| **hsa-miR-599** | GUUGUGUCAGUUUAUCAAAC | 25.05 | ± 0.89 | 33.21 | ± 1.91 |
| **hsa-miR-600** | ACUUACAGACAAGAGCCUUGCUC | 26.93 | ± 0.40 | 37.81 | ± 3.91 |
| **hsa-miR-601** | UGGUCUAGGAUUGUUGGAGGAG | 22.50 | ± 0.83 | 31.12 | ± 0.73 |
| **hsa-miR-602** | GACACGGGCGACAGCUGCGGCCC | 31.76 | ± 1.57 | 35.12 | ± 0.00 |
| **hsa-miR-603** | CACACACUGCAAUUACUUUUGC | 31.15 | ± 1.57 | 23.98 | ± 25.57 |
| **hsa-miR-604** | AGGCUGCGGAAUUCAGGAC | 36.14 | ± 1.58 | 33.04 | ± 4.01 |
| **hsa-miR-605** | UAAAUCCCAUGGUGCCUUCUCCU | 26.15 | ± 1.57 | 29.13 | ± 2.34 |
| **hsa-miR-606** | AAACUACUGAAAAUCAAAGAU | 23.53 | ± 5.81 | 29.78 | ± 3.85 |
| **hsa-miR-607** | GUUCAAAUCCAGAUCUAUAAC | 29.31 | ± 2.54 | 32.82 | ± 0.89 |
| **hsa-miR-608** | AGGGGUGGUGUUGGGACAGCUCCGU | 33.15 | ± 4.90 | 32.19 | ± 1.03 |
| **hsa-miR-609** | AGGGUGUUUCUCUCAUCUCU | 33.06 | ± 1.22 | 33.62 | ± 2.06 |
| **hsa-miR-610** | UGAGCUAAAUGUGUGCUGGGA | 29.84 | ± 4.10 | 31.30 | ± 0.44 |
| **hsa-miR-611** | GCGAGGACCCCUCGGGGUCUGAC | 36.90 | ± 4.04 | 28.33 | ± 0.60 |
| **hsa-miR-612** | GCUGGGCAGGGCUUCUGAGCUCCUU | 25.71 | ± 5.42 | 24.89 | ± 1.55 |
| **hsa-miR-613** | AGGAAUGUUCCUUCUUUGCC | 28.97 | ± 2.49 | 25.76 | ± 1.82 |
| **hsa-miR-614** | GAACGCCUGUUCUUGCCAGGUGG | 36.52 | ± 5.95 | 30.34 | ± 1.66 |
| **hsa-miR-615-3p** | UCCGAGCCUGGGUCUCCCUCUU | 31.23 | ± 4.01 | 25.67 | ± 4.56 |
| **hsa-miR-615-5p** | GGGGGUCCCCGGUGCUCGGAUC | 26.44 | ± 0.85 | 29.77 | ± 1.91 |
| **hsa-miR-616** | AGUCAUUGGAGGGUUUGAGCAG | 28.71 | ± 3.00 | 31.37 | ± 1.20 |
| **hsa-miR-617** | AGACUUCCCAUUUGAAGGUGGC | 30.62 | ± 0.68 | 33.74 | ± 1.06 |
| **hsa-miR-618** | AAACUCUACUUGUCCUUCUGAGU | 31.02 | ± 6.11 | 34.39 | ± 0.78 |
| **hsa-miR-619** | GACCUGGACAUGUUUGUGCCCAGU | 28.42 | ± 3.07 | 30.01 | ± 0.63 |
| **hsa-miR-620** | AUGGAGAUAGAUAUAGAAAU | 27.34 | ± 9.28 | 28.06 | ± 1.29 |
| **hsa-miR-621** | GGCUAGCAACAGCGCUUACCU | 30.08 | ± 1.72 | 32.54 | ± 1.11 |
| **hsa-miR-622** | ACAGUCUGCUGAGGUUGGAGC | 31.29 | ± 1.72 | 29.80 | ± 0.19 |
| **hsa-miR-623** | AUCCCUUGCAGGGGCUGUUGGGU | 25.84 | ± 3.22 | 33.54 | ± 3.22 |
| **hsa-miR-624** | CACAAGGUAUUGGUAUUACCU | 25.84 | ± 3.22 | 34.93 | ± 1.85 |
| **hsa-miR-625** | AGGGGGAAAGUUCUAUAGUCC | 24.09 | ± 0.68 | 24.53 | ± 8.92 |
| **hsa-miR-626** | AGCUGUCUGAAAAUGUCUU | 28.36 | ± 0.86 | 33.14 | ± 3.22 |
| **hsa-miR-627** | GUGAGUCUCUAAGAAAAGAGGA | 26.81 | ± 0.89 | 32.79 | ± 3.22 |
| **hsa-miR-628-3p** | UCUAGUAAGAGUGGCAGUCGA | 28.60 | ± 0.76 | 29.43 | ± 0.16 |
| **hsa-miR-628-5p** | AUGCUGACAUAUUUACUAGAGG | 25.51 | ± 1.89 | 30.88 | ± 1.62 |
| **hsa-miR-629** | UGGGUUUACGUUGGGAGAACU | 32.38 | ± 0.46 | 20.88 | ± 3.16 |
| **hsa-miR-630** | AGUAUUCUGUACCAGGGAAGGU | 35.16 | ± 6.84 | 33.94 | ± 1.73 |
| **hsa-miR-631** | AGACCUGGCCCAGACCUCAGC | 30.67 | ± 5.20 | 29.41 | ± 1.89 |
| **hsa-miR-632** | GUGUCUGCUUCCUGUGGGA | 29.48 | ± 2.17 | 31.54 | ± 0.92 |
| **hsa-miR-633** | CUAAUAGUAUCUACCACAAUAAA | 31.89 | ± 1.89 | 32.01 | ± 0.76 |
| **hsa-miR-634** | AACCAGCACCCCAACUUUGGAC | 31.25 | ± 0.46 | 32.48 | ± 0.42 |
| **hsa-miR-635** | ACUUGGGCACUGAAACAAUGUCC | 31.49 | ± 1.44 | 25.77 | ± 1.44 |
| **hsa-miR-636** | UGUGCUUGCUCGUCCCGCCCGCA | 21.37 | ± 0.85 | 24.65 | ± 0.68 |
| **hsa-miR-637** | ACUGGGGGCUUUCGGGCUCUGCGU | 34.10 | ± 4.57 | 27.62 | ± 0.50 |
| **hsa-miR-638** | AGGGAUCGCGGGCGGGUGGCGGCCU | 33.82 | ± 3.57 | 28.15 | ± 1.10 |
| **hsa-miR-639** | AUCGCUGCGGUUGCGAGCGCUGU | 25.51 | ± 1.15 | 34.77 | ± 0.84 |
| **hsa-miR-640** | AUGAUCCAGGAACCUGCCUCU | 30.22 | ± 4.09 | 30.64 | ± 2.84 |
| **hsa-miR-641** | AAAGACAUAGGAUAGAGUCACCUC | 30.58 | ± 3.08 | 32.33 | ± 1.89 |
| **hsa-miR-642** | GUCCCUCUCCAAAUGUGUCUUG | 22.43 | ± 3.45 | 28.90 | ± 0.36 |
| **hsa-miR-643** | ACUUGUAUGCUAGCUCAGGUAG | 29.50 | ± 1.45 | 34.68 | ± 4.83 |
| **hsa-miR-644** | AGUGUGGCUUUCUUAGAGC | 32.18 | ± 5.00 | 28.17 | ± 5.44 |
| **hsa-miR-645** | UCUAGGCUGGUACUGCUGA | 29.25 | ± 4.09 | 31.18 | ± 5.46 |
| **hsa-miR-646** | AAGCAGCUGCCUCUGAGGC | 31.10 | ± 4.59 | 25.55 | ± 1.93 |
| **hsa-miR-647** | GUGGCUGCACUCACUUCCUUC | 26.59 | ± 1.66 | 32.34 | ± 3.99 |
| **hsa-miR-648** | AAGUGUGCAGGGCACUGGU | 40.00 | ± 1.72 | 32.02 | ± 0.50 |
| **hsa-miR-649** | AAACCUGUGUUGUUCAAGAGUC | 30.21 | ± 4.51 | 29.02 | ± 0.73 |
| **hsa-miR-650** | AGGAGGCAGCGCUCUCAGGAC | 32.62 | ± 1.66 | 29.68 | ± 1.34 |
| **hsa-miR-651** | UUUAGGAUAAGCUUGACUUUUG | 31.36 | ± 1.72 | 25.90 | ± 3.45 |
| **hsa-miR-652** | AAUGGCGCCACUAGGGUUGUG | 36.23 | ± 2.88 | 29.48 | ± 1.94 |
| **hsa-miR-653** | GUGUUGAAACAAUCUCUACUG | 30.78 | ± 2.25 | 33.78 | ± 2.12 |
| **hsa-miR-654-3p** | UAUGUCUGCUGACCAUCACCUU | 31.38 | ± 2.84 | 23.14 | ± 1.66 |
| **hsa-miR-654-5p** | UGGUGGGCCGCAGAACAUGUGC | 32.35 | ± 4.97 | 29.72 | ± 2.64 |
| **hsa-miR-655** | AUAAUACAUGGUUAACCUCUUU | 26.64 | ± 1.85 | 28.82 | ± 2.25 |
| **hsa-miR-656** | AAUAUUAUACAGUCAACCUCU | 27.36 | ± 4.98 | 27.50 | ± 2.84 |
| **hsa-miR-657** | GGCAGGUUCUCACCCUCUCUAGG | 30.63 | ± 3.25 | 29.14 | ± 4.97 |
| **hsa-miR-658** | GGCGGAGGGAAGUAGGUCCGUUGGU | 30.14 | ± 2.25 | 30.70 | ± 2.18 |
| **hsa-miR-659** | CUUGGUUCAGGGAGGGUCCCCA | 26.40 | ± 1.93 | 27.43 | ± 1.69 |
| **hsa-miR-660** | UACCCAUUGCAUAUCGGAGUUG | 28.58 | ± 0.69 | 30.27 | ± 1.87 |
| **hsa-miR-661** | UGCCUGGGUCUCUGGCCUGCGCGU | 22.56 | ± 0.12 | 24.71 | ± 1.59 |
| **hsa-miR-662** | UCCCACGUUGUGGCCCAGCAG | 26.58 | ± 2.43 | 31.23 | ± 3.89 |
| **hsa-miR-663** | AGGCGGGGCGCCGCGGGACCGC | 30.82 | ± 7.96 | 28.06 | ± 0.48 |
| **hsa-miR-663b** | GGUGGCCCGGCCGUGCCUGAGG | 27.70 | ± 2.37 | 31.70 | ± 0.32 |
| **hsa-miR-664** | UAUUCAUUUAUCCCCAGCCUACA | 26.33 | ± 3.43 | 29.53 | ± 0.65 |
| **hsa-miR-665** | ACCAGGAGGCUGAGGCCCCU | 40.00 | ± 0.36 | 30.53 | ± 2.00 |
| **hsa-miR-668** | UGUCACUCGGCUCGGCCCACUAC | 31.00 | ± 1.13 | 27.59 | ± 1.77 |
| **hsa-miR-671-3p** | UCCGGUUCUCAGGGCUCCACC | 21.01 | ± 1.99 | 24.39 | ± 1.73 |
| **hsa-miR-671-5p** | AGGAAGCCCUGGAGGGGCUGGAG | 28.02 | ± 1.89 | 33.71 | ± 0.47 |
| **hsa-miR-675** | UGGUGCGGAGAGGGCCCACAGUG | 34.21 | ± 4.19 | 29.97 | ± 0.55 |
| **hsa-miR-708** | AAGGAGCUUACAAUCUAGCUGGG | 31.43 | ± 0.59 | 33.01 | ± 5.30 |
| **hsa-miR-720** | UCUCGCUGGGGCCUCCA | 24.46 | ± 1.55 | 25.67 | ± 2.34 |
| **hsa-miR-744** | UGCGGGGCUAGGGCUAACAGCA | 28.75 | ± 3.06 | 29.61 | ± 0.76 |
| **hsa-miR-758** | UUUGUGACCUGGUCCACUAACC | 30.47 | ± 3.08 | 28.28 | ± 6.67 |
| **hsa-miR-760** | CGGCUCUGGGUCUGUGGGGA | 27.51 | ± 0.48 | 28.99 | ± 2.18 |
| **hsa-miR-765** | UGGAGGAGAAGGAAGGUGAUG | 31.33 | ± 1.00 | 24.72 | ± 3.42 |
| **hsa-miR-766** | ACUCCAGCCCCACAGCCUCAGC | 25.41 | ± 1.56 | 26.80 | ± 5.36 |
| **hsa-miR-767-3p** | UCUGCUCAUACCCCAUGGUUUCU | 29.28 | ± 1.77 | 28.47 | ± 1.56 |
| **hsa-miR-767-5p** | UGCACCAUGGUUGUCUGAGCAUG | 40.00 | ± 1.56 | 33.61 | ± 5.53 |
| **hsa-miR-769-3p** | CUGGGAUCUCCGGGGUCUUGGUU | 29.33 | ± 1.81 | 34.12 | ± 4.53 |
| **hsa-miR-769-5p** | UGAGACCUCUGGGUUCUGAGCU | 26.21 | ± 1.09 | 34.06 | ± 0.23 |
| **hsa-miR-770-5p** | UCCAGUACCACGUGUCAGGGCCA | 24.01 | ± 2.80 | 29.20 | ± 0.56 |
| **hsa-miR-802** | CAGUAACAAAGAUUCAUCCUUGU | 23.63 | ± 1.44 | 23.64 | ± 0.95 |
| **hsa-miR-873** | GCAGGAACUUGUGAGUCUCCU | 30.63 | ± 1.56 | 24.53 | ± 0.78 |
| **hsa-miR-874** | CUGCCCUGGCCCGAGGGACCGA | 26.65 | ± 1.40 | 26.73 | ± 1.34 |
| **hsa-miR-875-3p** | CCUGGAAACACUGAGGUUGUG | 30.94 | ± 4.22 | 26.86 | ± 4.51 |
| **hsa-miR-875-5p** | UAUACCUCAGUUUUAUCAGGUG | 29.01 | ± 3.22 | 24.77 | ± 3.44 |
| **hsa-miR-876-3p** | UGGUGGUUUACAAAGUAAUUCA | 32.54 | ± 3.48 | 36.31 | ± 2.73 |
| **hsa-miR-876-5p** | UGGAUUUCUUUGUGAAUCACCA | 30.83 | ± 3.55 | 35.02 | ± 3.12 |
| **hsa-miR-877** | GUAGAGGAGAUGGCGCAGGG | 32.14 | ± 6.47 | 27.90 | ± 3.51 |
| **hsa-miR-885-3p** | AGGCAGCGGGGUGUAGUGGAUA | 27.34 | ± 4.51 | 32.30 | ± 8.31 |
| **hsa-miR-885-5p** | UCCAUUACACUACCCUGCCUCU | 30.22 | ± 3.44 | 31.33 | ± 0.26 |
| **hsa-miR-886-3p** | CGCGGGUGCUUACUGACCCUU | 30.95 | ± 2.73 | 33.96 | ± 4.22 |
| **hsa-miR-886-5p** | CGGGUCGGAGUUAGCUCAAGCGG | 28.90 | ± 3.12 | 21.94 | ± 3.38 |
| **hsa-miR-887** | GUGAACGGGCGCCAUCCCGAGG | 31.83 | ± 3.46 | 33.69 | ± 1.43 |
| **hsa-miR-888** | UACUCAAAAAGCUGUCAGUCA | 27.69 | ± 3.48 | 30.25 | ± 1.53 |
| **hsa-miR-889** | UUAAUAUCGGACAACCAUUGU | 29.36 | ± 3.55 | 27.41 | ± 1.48 |
| **hsa-miR-890** | UACUUGGAAAGGCAUCAGUUG | 31.11 | ± 3.61 | 25.33 | ± 1.43 |
| **hsa-miR-891a** | UGCAACGAACCUGAGCCACUGA | 25.80 | ± 1.49 | 29.39 | ± 1.17 |
| **hsa-miR-891b** | UGCAACUUACCUGAGUCAUUGA | 31.44 | ± 5.55 | 27.55 | ± 0.96 |
| **hsa-miR-892a** | CACUGUGUCCUUUCUGCGUAG | 40.00 | ± 7.99 | 27.15 | ± 1.02 |
| **hsa-miR-892b** | CACUGGCUCCUUUCUGGGUAGA | 30.25 | ± 1.07 | 33.32 | ± 1.68 |
| **hsa-miR-920** | GGGGAGCUGUGGAAGCAGUA | 28.69 | ± 0.96 | 39.97 | ± 2.34 |
| **hsa-miR-921** | CUAGUGAGGGACAGAACCAGGAUUC | 25.73 | ± 2.31 | 25.69 | ± 4.23 |
| **hsa-miR-922** | GCAGCAGAGAAUAGGACUACGUC | 33.71 | ± 6.10 | 26.04 | ± 4.43 |
| **hsa-miR-924** | AGAGUCUUGUGAUGUCUUGC | 28.12 | ± 3.48 | 24.45 | ± 2.31 |
| **hsa-miR-933** | UGUGCGCAGGGAGACCUCUCCC | 36.95 | ± 4.31 | 24.45 | ± 0.00 |
| **hsa-miR-934** | UGUCUACUACUGGAGACACUGG | 27.64 | ± 2.62 | 32.09 | ± 3.18 |
| **hsa-miR-935** | CCAGUUACCGCUUCCGCUACCGC | 40.00 | ± 0.00 | 28.20 | ± 8.82 |
| **hsa-miR-936** | ACAGUAGAGGGAGGAAUCGCAG | 40.00 | ± 3.18 | 30.68 | ± 1.88 |
| **hsa-miR-937** | AUCCGCGCUCUGACUCUCUGCC | 30.48 | ± 2.70 | 23.57 | ± 6.07 |
| **hsa-miR-938** | UGCCCUUAAAGGUGAACCCAGU | 27.26 | ± 1.88 | 33.56 | ± 3.18 |
| **hsa-miR-939** | UGGGGAGCUGAGGCUCUGGGGGUG | 29.44 | ± 0.18 | 30.61 | ± 1.86 |
| **hsa-miR-940** | AAGGCAGGGCCCCCGCUCCCC | 20.24 | ± 1.94 | 22.25 | ± 1.45 |
| **hsa-miR-941** | CACCCGGCUGUGUGCACAUGUGC | 40.00 | ± 0.23 | 22.45 | ± 0.32 |
| **hsa-miR-942** | UCUUCUCUGUUUUGGCCAUGUG | 26.53 | ± 1.30 | 28.90 | ± 1.43 |
| **hsa-miR-943** | CUGACUGUUGCCGUCCUCCAG | 24.84 | ± 1.49 | 27.34 | ± 8.90 |
| **hsa-miR-944** | AAAUUAUUGUACAUCGGAUGAG | 26.63 | ± 6.38 | 25.84 | ± 2.56 |
| **hsa-miR-1178** | UUGCUCACUGUUCUUCCCUAG | 25.97 | ± 19.61 | 29.59 | ± 0.64 |
| **hsa-miR-1179** | AAGCAUUCUUUCAUUGGUUGG | 26.90 | ± 2.64 | 25.60 | ± 0.78 |
| **hsa-miR-1180** | UUUCCGGCUCGCGUGGGUGUGU | 27.27 | ± 2.74 | 28.60 | ± 9.63 |
| **hsa-miR-1181** | CCGUCGCCGCCACCCGAGCCG | 30.04 | ± 8.66 | 25.50 | ± 1.42 |
| **hsa-miR-1182** | GAGGGUCUUGGGAGGGAUGUGAC | 27.37 | ± 2.64 | 29.27 | ± 5.50 |
| **hsa-miR-1183** | CACUGUAGGUGAUGGUGAGAGUGGGCA | 29.45 | ± 9.31 | 26.30 | ± 0.96 |
| **hsa-miR-1184** | CCUGCAGCGACUUGAUGGCUUCC | 23.35 | ± 4.97 | 29.74 | ± 5.21 |
| **hsa-miR-1185** | AGAGGAUACCCUUUGUAUGUU | 31.52 | ± 3.27 | 25.53 | ± 3.09 |
| **hsa-miR-1197** | UAGGACACAUGGUCUACUUCU | 27.42 | ± 4.43 | 27.96 | ± 3.12 |
| **hsa-miR-1200** | CUCCUGAGCCAUUCUGAGCCUC | 31.45 | ± 7.41 | 31.19 | ± 2.56 |
| **hsa-miR-1201** | AGCCUGAUUAAACACAUGCUCUGA | 25.15 | ± 3.02 | 29.70 | ± 3.54 |
| **hsa-miR-1202** | GUGCCAGCUGCAGUGGGGGAG | 23.88 | ± 1.62 | 23.81 | ± 5.61 |
| **hsa-miR-1203** | CCCGGAGCCAGGAUGCAGCUC | 27.19 | ± 18.12 | 35.17 | ± 0.32 |
| **hsa-miR-1204** | UCGUGGCCUGGUCUCCAUUAU | 22.50 | ± 4.10 | 33.17 | ± 5.01 |
| **hsa-miR-1205** | UCUGCAGGGUUUGCUUUGAG | 32.15 | ± 5.47 | 35.68 | ± 4.02 |
| **hsa-miR-1206** | UGUUCAUGUAGAUGUUUAAGC | 32.03 | ± 3.01 | 38.05 | ± 3.02 |
| **hsa-miR-1207-3p** | UCAGCUGGCCCUCAUUUC | 24.38 | ± 8.67 | 31.95 | ± 2.48 |
| **hsa-miR-1207-5p** | UGGCAGGGAGGCUGGGAGGGG | 24.51 | ± 0.44 | 28.39 | ± 2.19 |
| **hsa-miR-1208** | UCACUGUUCAGACAGGCGGA | 26.06 | ± 2.10 | 25.93 | ± 1.47 |
| **hsa-miR-1224-3p** | CCCCACCUCCUCUCUCCUCAG | 28.80 | ± 3.53 | 26.73 | ± 2.51 |
| **hsa-miR-1224-5p** | GUGAGGACUCGGGAGGUGG | 28.23 | ± 3.72 | 29.42 | ± 0.71 |
| **hsa-miR-1225-3p** | UGAGCCCCUGUGCCGCCCCCAG | 22.31 | ± 0.21 | 24.08 | ± 1.43 |
| **hsa-miR-1225-5p** | GUGGGUACGGCCCAGUGGGGGG | 26.39 | ± 2.75 | 29.81 | ± 0.85 |
| **hsa-miR-1226** | UCACCAGCCCUGUGUUCCCUAG | 26.99 | ± 0.39 | 28.13 | ± 4.02 |
| **hsa-miR-1227** | CGUGCCACCCUUUUCCCCAG | 31.38 | ± 1.22 | 29.24 | ± 4.05 |
| **hsa-miR-1228** | UCACACCUGCCUCGCCCCCC | 26.07 | ± 12.28 | 24.50 | ± 1.21 |
| **hsa-miR-1229** | CUCUCACCACUGCCCUCCCACAG | 27.23 | ± 4.92 | 28.32 | ± 1.85 |
| **hsa-miR-1231** | GUGUCUGGGCGGACAGCUGC | 31.65 | ± 3.71 | 22.51 | ± 1.56 |
| **hsa-miR-1233** | UGAGCCCUGUCCUCCCGCAG | 25.78 | ± 3.19 | 26.22 | ± 2.88 |
| **hsa-miR-1234** | UCGGCCUGACCACCCACCCCAC | 34.57 | ± 9.41 | 26.09 | ± 1.61 |
| **hsa-miR-1236** | CCUCUUCCCCUUGUCUCUCCAG | 28.86 | ± 4.32 | 29.13 | ± 2.64 |
| **hsa-miR-1237** | UCCUUCUGCUCCGUCCCCCAG | 28.46 | ± 3.17 | 28.69 | ± 1.82 |
| **hsa-miR-1238** | CUUCCUCGUCUGUCUGCCCC | 24.66 | ± 2.55 | 25.69 | ± 1.71 |
| **hsa-miR-1243** | AACUGGAUCAAUUAUAGGAGUG | 24.50 | ± 2.25 | 34.05 | ± 2.25 |
| **hsa-miR-1244** | AAGUAGUUGGUUUGUAUGAGAUGGUU | 33.52 | ± 3.43 | 32.65 | ± 3.43 |
| **hsa-miR-1245** | AAGUGAUCUAAAGGCCUACAU | 30.84 | ± 2.39 | 33.43 | ± 2.39 |
| **hsa-miR-1246** | AAUGGAUUUUUGGAGCAGG | 40.00 | ± 2.44 | 23.63 | ± 3.51 |
| **hsa-miR-1247** | ACCCGUCCCGUUCGUCCCCGGA | 22.21 | ± 5.00 | 26.35 | ± 1.62 |
| **hsa-miR-1248** | ACCUUCUUGUAUAAGCACUGUGCUAAA | 34.40 | ± 2.44 | 24.31 | ± 5.62 |
| **hsa-miR-1249** | ACGCCCUUCCCCCCCUUCUUCA | 27.30 | ± 1.74 | 20.02 | ± 4.24 |
| **hsa-miR-1250** | ACGGUGCUGGAUGUGGCCUUU | 29.81 | ± 3.16 | 30.32 | ± 0.38 |
| **hsa-miR-1251** | ACUCUAGCUGCCAAAGGCGCU | 28.23 | ± 1.79 | 32.37 | ± 1.79 |
| **hsa-miR-1252** | AGAAGGAAAUUGAAUUCAUUUA | 27.16 | ± 1.69 | 30.37 | ± 1.69 |
| **hsa-miR-1253** | AGAGAAGAAGAUCAGCCUGCA | 32.43 | ± 6.59 | 29.04 | ± 0.93 |
| **hsa-miR-1254** | AGCCUGGAAGCUGGAGCCUGCAGU | 31.35 | ± 1.19 | 33.97 | ± 0.01 |
| **hsa-miR-1255a** | AGGAUGAGCAAAGAAAGUAGAUU | 32.52 | ± 1.79 | 29.53 | ± 4.76 |
| **hsa-miR-1255b** | CGGAUGAGCAAAGAAAGUGGUU | 20.71 | ± 0.77 | 32.72 | ± 1.79 |
| **hsa-miR-1256** | AGGCAUUGACUUCUCACUAGCU | 40.00 | ± 0.59 | 29.92 | ± 0.52 |
| **hsa-miR-1257** | AGUGAAUGAUGGGUUCUGACC | 22.91 | ± 3.70 | 22.93 | ± 8.83 |
| **hsa-miR-1258** | AGUUAGGAUUAGGUCGUGGAA | 28.79 | ± 2.79 | 26.40 | ± 0.99 |
| **hsa-miR-1259** | AUAUAUGAUGACUUAGCUUUU | 32.50 | ± 3.21 | 34.16 | ± 5.27 |
| **hsa-miR-1260** | AUCCCACCUCUGCCACCA | 17.46 | ± 3.68 | 23.21 | ± 0.50 |
| **hsa-miR-1261** | AUGGAUAAGGCUUUGGCUU | 24.78 | ± 4.78 | 29.43 | ± 6.71 |
| **hsa-miR-1262** | AUGGGUGAAUUUGUAGAAGGAU | 28.30 | ± 2.81 | 36.84 | ± 3.21 |
| **hsa-miR-1263** | AUGGUACCCUGGCAUACUGAGU | 25.51 | ± 4.01 | 30.11 | ± 0.93 |
| **hsa-miR-1264** | CAAGUCUUAUUUGAGCACCUGUU | 27.86 | ± 0.32 | 30.09 | ± 1.82 |
| **hsa-miR-1265** | CAGGAUGUGGUCAAGUGUUGUU | 27.64 | ± 1.18 | 28.48 | ± 2.64 |
| **hsa-miR-1266** | CCUCAGGGCUGUAGAACAGGGCU | 36.81 | ± 5.53 | 34.63 | ± 5.57 |
| **hsa-miR-1267** | CCUGUUGAAGUGUAAUCCCCA | 28.47 | ± 2.33 | 33.81 | ± 2.33 |
| **hsa-miR-1268** | CGGGCGUGGUGGUGGGGG | 29.58 | ± 9.10 | 28.85 | ± 0.69 |
| **hsa-miR-1269** | CUGGACUGAGCCGUGCUACUGG | 29.17 | ± 3.53 | 31.91 | ± 5.92 |
| **hsa-miR-1270** | CUGGAGAUAUGGAAGAGCUGUGU | 29.64 | ± 5.92 | 31.89 | ± 1.32 |
| **hsa-miR-1271** | CUUGGCACCUAGCAAGCACUCA | 30.37 | ± 3.84 | 23.78 | ± 4.80 |
| **hsa-miR-1272** | GAUGAUGAUGGCAGCAAAUUCUGAAA | 33.31 | ± 4.64 | 34.42 | ± 4.77 |
| **hsa-miR-1273** | GGGCGACAAAGCAAGACUCUUUCUU | 34.12 | ± 5.27 | 23.29 | ± 2.11 |
| **hsa-miR-1274a** | GUCCCUGUUCAGGCGCCA | 30.41 | ± 0.79 | 24.29 | ± 0.13 |
| **hsa-miR-1274b** | UCCCUGUUCGGGCGCCA | 29.64 | ± 1.46 | 15.87 | ± 1.90 |
| **hsa-miR-1275** | GUGGGGGAGAGGCUGUC | 32.39 | ± 10.77 | 28.59 | ± 2.86 |
| **hsa-miR-1276** | UAAAGAGCCCUGUGGAGACA | 28.64 | ± 0.71 | 29.76 | ± 1.17 |
| **hsa-miR-1277** | UACGUAGAUAUAUAUGUAUUUU | 31.79 | ± 2.36 | 31.15 | ± 0.78 |
| **hsa-miR-1278** | UAGUACUGUGCAUAUCAUCUAU | 25.16 | ± 5.34 | 32.92 | ± 2.22 |
| **hsa-miR-1279** | UCAUAUUGCUUCUUUCU | 25.25 | ± 2.14 | 24.26 | ± 2.35 |
| **hsa-miR-1280** | UCCCACCGCUGCCACCC | 24.26 | ± 3.98 | 22.44 | ± 0.56 |
| **hsa-miR-1281** | UCGCCUCCUCCUCUCCC | 29.74 | ± 3.88 | 24.27 | ± 1.25 |
| **hsa-miR-1282** | UCGUUUGCCUUUUUCUGCUU | 28.35 | ± 4.34 | 22.46 | ± 1.59 |
| **hsa-miR-1283** | UCUACAAAGGAAAGCGCUUUCU | 31.26 | ± 4.79 | 24.71 | ± 1.40 |
| **hsa-miR-1284** | UCUAUACAGACCCUGGCUUUUC | 26.77 | ± 2.39 | 30.95 | ± 3.12 |
| **hsa-miR-1285** | UCUGGGCAACAAAGUGAGACCU | 25.68 | ± 4.96 | 26.74 | ± 2.06 |
| **hsa-miR-1286** | UGCAGGACCAAGAUGAGCCCU | 27.43 | ± 3.34 | 28.91 | ± 3.03 |
| **hsa-miR-1287** | UGCUGGAUCAGUGGUUCGAGUC | 28.62 | ± 2.66 | 24.42 | ± 3.70 |
| **hsa-miR-1288** | UGGACUGCCCUGAUCUGGAGA | 25.07 | ± 0.68 | 29.98 | ± 2.36 |
| **hsa-miR-1289** | UGGAGUCCAGGAAUCUGCAUUUU | 29.42 | ± 4.28 | 24.36 | ± 2.77 |
| **hsa-miR-1290** | UGGAUUUUUGGAUCAGGGA | 29.43 | ± 2.38 | 28.39 | ± 2.65 |
| **hsa-miR-1291** | UGGCCCUGACUGAAGACCAGCAGU | 32.29 | ± 1.06 | 25.83 | ± 2.45 |
| **hsa-miR-1292** | UGGGAACGGGUUCCGGCAGACGCUG | 29.67 | ± 1.44 | 26.74 | ± 2.36 |
| **hsa-miR-1293** | UGGGUGGUCUGGAGAUUUGUGC | 26.07 | ± 4.04 | 21.03 | ± 6.74 |
| **hsa-miR-1294** | UGUGAGGUUGGCAUUGUUGUCU | 29.56 | ± 2.11 | 31.94 | ± 1.74 |
| **hsa-miR-1295** | UUAGGCCGCAGAUCUGGGUGA | 27.37 | ± 4.06 | 32.59 | ± 1.77 |
| **hsa-miR-1296** | UUAGGGCCCUGGCUCCAUCUCC | 29.58 | ± 4.88 | 28.06 | ± 1.80 |
| **hsa-miR-1297** | UUCAAGUAAUUCAGGUG | 26.95 | ± 2.12 | 28.91 | ± 4.65 |
| **hsa-miR-1298** | UUCAUUCGGCUGUCCAGAUGUA | 31.92 | ± 1.77 | 24.83 | ± 1.68 |
| **hsa-miR-1299** | UUCUGGAAUUCUGUGUGAGGGA | 40.00 | ± 1.89 | 28.82 | ± 0.80 |
| **hsa-miR-1300** | UUGAGAAGGAGGCUGCUG | 29.42 | ± 1.46 | 30.18 | ± 1.77 |
| **hsa-miR-1301** | UUGCAGCUGCCUGGGAGUGACUUC | 30.76 | ± 1.85 | 31.68 | ± 1.89 |
| **hsa-miR-1302** | UUGGGACAUACUUAUGCUAAA | 31.49 | ± 1.77 | 27.73 | ± 1.46 |
| **hsa-miR-1303** | UUUAGAGACGGGGUCUUGCUCU | 40.00 | ± 1.80 | 28.32 | ± 1.85 |
| **hsa-miR-1304** | UUUGAGGCUACAGUGAGAUGUG | 34.00 | ± 4.65 | 31.91 | ± 1.77 |
| **hsa-miR-1305** | UUUUCAACUCUAAUGGGAGAGA | 21.37 | ± 1.36 | 34.90 | ± 1.80 |
| **hsa-miR-1306** | ACGUUGGCUCUGGUGGUG | 30.40 | ± 1.34 | 27.98 | ± 4.65 |
| **hsa-miR-1307** | ACUCGGCGUGGCGUCGGUCGUG | 27.99 | ± 2.12 | 24.36 | ± 0.01 |
| **hsa-miR-1308** | GCAUGGGUGGUUCAGUGG | 23.29 | ± 1.65 | 25.92 | ± 1.11 |
| **hsa-miR-1321** | CAGGGAGGUGAAUGUGAU | 28.96 | ± 1.52 | 32.84 | ± 0.17 |
| **hsa-miR-1322** | GAUGAUGCUGCUGAUGCUG | 23.51 | ± 1.66 | 31.70 | ± 2.54 |
| **hsa-miR-1323** | UCAAAACUGAGGGGCAUUUUCU | 29.10 | ± 1.78 | 26.07 | ± 1.96 |
| **hsa-miR-1324** | CCAGACAGAAUUCUAUGCACUUUC | 28.43 | ± 1.21 | 30.19 | ± 1.52 |
| **hsa-miR-1468** | CUCCGUUUGCCUGUUUCGCUG | 31.93 | ± 1.59 | 24.83 | ± 1.59 |
| **hsa-miR-1469** | CUCGGCGCGGGGCGCGGGCUCC | 25.18 | ± 2.64 | 27.03 | ± 0.45 |
| **hsa-miR-1470** | GCCCUCCGCCCGUGCACCCCG | 29.64 | ± 2.65 | 25.24 | ± 4.26 |
| **hsa-miR-1471** | GCCCGCGUGUGGAGCCAGGUGU | 28.88 | ± 2.16 | 29.67 | ± 2.32 |
| **hsa-miR-1537** | AAAACCGUCUAGUUACAGUUGU | 35.18 | ± 4.08 | 29.71 | ± 1.45 |
| **hsa-miR-1538** | CGGCCCGGGCUGCUGCUGUUCCU | 30.05 | ± 4.22 | 31.31 | ± 1.81 |
| **hsa-miR-1539** | UCCUGCGCGUCCCAGAUGCCC | 28.84 | ± 3.22 | 29.84 | ± 1.56 |
| **hsa-miR-1825** | UCCAGUGCCCUCCUCUCC | 22.07 | ± 0.56 | 23.72 | ± 1.58 |
| **hsa-miR-1826** | AUUGAUCAUCGACACUUCGAACGCAAU | 27.02 | ± 0.36 | 28.10 | ± 2.86 |
| **hsa-miR-1827** | UGAGGCAGUAGAUUGAAU | 29.10 | ± 4.93 | 21.80 | ± 3.22 |
| **hsa-miR-1908** | CGGCGGGGACGGCGAUUGGUC | 30.86 | ± 3.00 | 28.46 | ± 1.06 |
| **hsa-miR-1909** | CGCAGGGGCCGGGUGCUCACCG | 29.49 | ± 4.89 | 28.41 | ± 0.05 |
| **hsa-miR-1910** | CCAGUCCUGUGCCUGCCGCCU | 27.36 | ± 0.55 | 25.74 | ± 8.50 |
| **hsa-miR-1911** | UGAGUACCGCCAUGUCUGUUGGG | 28.57 | ± 1.69 | 25.60 | ± 4.89 |
| **hsa-miR-1912** | UACCCAGAGCAUGCAGUGUGAA | 23.29 | ± 1.68 | 30.69 | ± 1.69 |
| **hsa-miR-1913** | UCUGCCCCCUCCGCUGCUGCCA | 21.29 | ± 0.33 | 22.70 | ± 1.11 |
| **hsa-miR-1914** | CCCUGUGCCCGGCCCACUUCUG | 23.93 | ± 1.32 | 25.40 | ± 1.51 |
| **hsa-miR-1915** | CCCCAGGGCGACGCGGCGGG | 29.25 | ± 9.42 | 23.76 | ± 1.92 |
| **hsa-miR-1972** | UCAGGCCAGGCACAGUGGCUCA | 27.48 | ± 2.48 | 22.90 | ± 1.78 |
| **hsa-miR-1973** | ACCGUGCAAAGGUAGCAUA | 26.84 | ± 6.48 | 25.18 | ± 2.35 |
| **hsa-miR-1974** | UGGUUGUAGUCCGUGCGAGAAUA | 25.95 | ± 3.89 | 23.66 | ± 1.87 |
| **hsa-miR-1975** | CCCCCACAACCGCGCUUGACUAGCU | 26.72 | ± 0.70 | 23.41 | ± 3.28 |
| **hsa-miR-1976** | CCUCCUGCCCUCCUUGCUGU | 28.48 | ± 1.46 | 32.78 | ± 3.63 |
| **hsa-miR-1977** | GAUUAGGGUGCUUAGCUGUUAA | 23.79 | ± 1.90 | 24.21 | ± 3.28 |
| **hsa-miR-1978** | GGUUUGGUCCUAGCCUUUCUA | 23.83 | ± 0.55 | 28.61 | ± 0.91 |
| **hsa-miR-1979** | CUCCCACUGCUUCACUUGACUA | 24.46 | ± 4.15 | 21.85 | ± 9.99 |
| **hsa-miR-2052** | UGUUUUGAUAACAGUAAUGU | 23.29 | ± 2.00 | 22.44 | ± 2.74 |
| **hsa-miR-2053** | GUGUUAAUUAAACCUCUAUUUAC | 28.91 | ± 2.08 | 37.55 | ± 1.55 |
| **hsa-miR-2054** | CUGUAAUAUAAAUUUAAUUUAUU | 32.66 | ± 1.34 | 35.64 | ± 1.67 |
| **hsa-miR-2110** | UUGGGGAAACGGCCGCUGAGUG | 31.03 | ± 1.38 | 32.61 | ± 2.15 |
| **hsa-miR-2113** | AUUUGUGCUUGGCUCUGUCAC | 40.00 | ± 1.45 | 29.20 | ± 1.55 |
| **hsa-miR-449c** | UAGGCAGUGUAUUGCUAGCGGCUGU | 22.15 | ± 1.48 | 24.78 | ± 1.55 |
| **hsa-miR-762** | GGGGCUGGGGCCGGGGCCGAGC | 40.00 | ± 0.66 | 33.15 | ± 4.87 |
| **hsa-miR-670** | GUCCCUGAGUGUAUGUGGUG | 30.32 | ± 4.87 | 39.48 | ± 4.87 |
| **hsa-miR-761** | GCAGCAGGGUGAAACUGACACA | 27.86 | ± 1.96 | 31.01 | ± 0.63 |
| **hsa-miR-764** | GCAGGUGCUCACUUGUCCUCCU | 27.25 | ± 1.46 | 30.06 | ± 1.37 |
| **hsa-miR-759** | GCAGAGUGCAAACAAUUUUGAC | 34.97 | ± 4.47 | 22.90 | ± 1.25 |
| **hsa-miR-2114** | UAGUCCCUUCCUUGAAGCGGUC | 29.46 | ± 2.17 | 32.67 | ± 0.48 |
| **hsa-miR-2115** | AGCUUCCAUGACUCCUGAUGGA | 33.61 | ± 0.75 | 25.56 | ± 1.04 |
| **hsa-miR-2116** | GGUUCUUAGCAUAGGAGGUCU | 33.34 | ± 2.47 | 27.20 | ± 2.42 |
| **hsa-miR-2117** | UGUUCUCUUUGCCAAGGACAG | 36.32 | ± 1.46 | 33.68 | ± 0.21 |
| **hsa-miR-548q** | GCUGGUGCAAAAGUAAUGGCGG | 26.76 | ± 2.06 | 28.76 | ± 0.68 |
| **hsa-miR-2276** | UCUGCAAGUGUCAGAGGCGAGG | 23.99 | ± 0.99 | 26.48 | ± 5.93 |
| **hsa-miR-2277** | UGACAGCGCCCUGCCUGGCUC | 27.67 | ± 2.12 | 29.73 | ± 1.25 |
| **hsa-miR-2278** | GAGAGCAGUGUGUGUUGCCUGG | 26.53 | ± 2.24 | 30.82 | ± 0.85 |
| **hsa-miR-711** | GGGACCCAGGGAGAGACGUAAG | 25.60 | ± 1.04 | 34.07 | ± 2.42 |
| **hsa-miR-718** | CUUCCGCCCCGCCGGGCGUCG | 26.70 | ± 1.75 | 28.40 | ± 7.48 |
| **hsa-miR-1184** | CCUGCAGCGACUUGAUGGCUUCC | 30.01 | ± 4.72 | 29.45 | ± 1.52 |
| **hsa-miR-1193** | GGGAUGGUAGACCGGUGACGUGC | 27.43 | ± 5.93 | 28.13 | ± 2.42 |
| **hsa-miR-1233** | UGAGCCCUGUCCUCCCGCAG | 29.63 | ± 4.13 | 29.36 | ± 0.21 |
| **hsa-miR-1244** | AAGUAGUUGGUUUGUAUGAGAUGGUU | 28.55 | ± 0.66 | 32.43 | ± 0.85 |
| **hsa-miR-1260b** | AUCCCACCACUGCCACCAU | 21.76 | ± 2.34 | 27.22 | ± 0.08 |
| **hsa-miR-1270** | CUGGAGAUAUGGAAGAGCUGUGU | 27.11 | ± 0.66 | 31.90 | ± 1.92 |
| **hsa-miR-1273c** | GGCGACAAAACGAGACCCUGUC | 25.45 | ± 3.79 | 27.72 | ± 0.19 |
| **hsa-miR-1273d** | GAACCCAUGAGGUUGAGGCUGCAGU | 29.54 | ± 4.13 | 28.57 | ± 2.62 |
| **hsa-miR-1302** | UUGGGACAUACUUAUGCUAAA | 29.75 | ± 7.80 | 25.47 | ± 1.46 |
| **hsa-miR-1972** | UCAGGCCAGGCACAGUGGCUCA | 32.99 | ± 0.87 | 26.56 | ± 2.05 |
| **hsa-miR-2355** | AUCCCCAGAUACAAUGGACAA | 29.50 | ± 5.26 | 22.47 | ± 2.89 |
| **hsa-miR-2861** | GGGGCCUGGCGGUGGGCGG | 24.40 | ± 1.13 | 26.07 | ± 2.03 |
| **hsa-miR-2909** | GUUAGGGCCAACAUCUCUUGG | 29.99 | ± 0.75 | 35.19 | ± 3.88 |
| **hsa-miR-3065-5p** | UCAACAAAAUCACUGAUGCUGGA | 29.87 | ± 0.81 | 25.70 | ± 0.87 |
| **hsa-miR-3065-3p** | UCAGCACCAGGAUAUUGUUGGAG | 31.04 | ± 0.75 | 34.82 | ± 0.75 |
| **hsa-miR-3074** | GAUAUCAGCUCAGUAGGCACCG | 27.84 | ± 3.23 | 26.49 | ± 1.18 |
| **hsa-miR-3115** | AUAUGGGUUUACUAGUUGGU | 32.92 | ± 1.46 | 28.38 | ± 5.61 |
| **hsa-miR-3116** | UGCCUGGAACAUAGUAGGGACU | 35.52 | ± 2.73 | 30.43 | ± 4.42 |
| **hsa-miR-3117** | AUAGGACUCAUAUAGUGCCAG | 30.06 | ± 6.80 | 33.45 | ± 5.81 |
| **hsa-miR-3118** | UGUGACUGCAUUAUGAAAAUUCU | 29.69 | ± 2.89 | 28.48 | ± 2.01 |
| **hsa-miR-3119** | UGGCUUUUAACUUUGAUGGC | 29.50 | ± 5.81 | 29.15 | ± 0.01 |
| **hsa-miR-3120** | CACAGCAAGUGUAGACAGGCA | 35.25 | ± 6.71 | 31.55 | ± 2.47 |
| **hsa-miR-3121** | UAAAUAGAGUAGGCAAAGGACA | 31.63 | ± 6.80 | 33.02 | ± 1.91 |
| **hsa-miR-3122** | GUUGGGACAAGAGGACGGUCUU | 33.22 | ± 2.89 | 32.42 | ± 2.73 |
| **hsa-miR-3123** | CAGAGAAUUGUUUAAUC | 29.75 | ± 2.37 | 16.90 | ± 1.35 |
| **hsa-miR-3124** | UUCGCGGGCGAAGGCAAAGUC | 29.90 | ± 4.79 | 28.26 | ± 1.22 |
| **hsa-miR-3125** | UAGAGGAAGCUGUGGAGAGA | 28.00 | ± 0.75 | 34.55 | ± 2.01 |
| **hsa-miR-3126-5p** | UGAGGGACAGAUGCCAGAAGCA | 34.26 | ± 2.47 | 31.44 | ± 2.08 |
| **hsa-miR-3126-3p** | CAUCUGGCAUCCGUCACACAGA | 33.27 | ± 2.40 | 33.16 | ± 0.37 |
| **hsa-miR-3127** | AUCAGGGCUUGUGGAAUGGGAAG | 31.84 | ± 2.73 | 28.03 | ± 0.77 |

| ***mature miRNA ID*** | ***Target microRNA Mature Sequence*** | ***Average Ct miR e-sEV*** | | ***Average Ct miR i-sEV*** | |
| --- | --- | --- | --- | --- | --- |
| **hsa-miR-3128** | UCUGGCAAGUAAAAAACUCUCAU | 24.49 | ± 2.12 | 27.38 | ± 1.95 |
| **hsa-miR-3129** | GCAGUAGUGUAGAGAUUGGUUU | 29.57 | ± 2.75 | 32.81 | ± 0.79 |
| **hsa-miR-3130-5p** | UACCCAGUCUCCGGUGCAGCC | 25.11 | ± 1.53 | 27.43 | ± 0.54 |
| **hsa-miR-3130-3p** | GCUGCACCGGAGACUGGGUAA | 25.31 | ± 2.97 | 29.69 | ± 0.74 |
| **hsa-miR-3131** | UCGAGGACUGGUGGAAGGGCCUU | 31.18 | ± 1.27 | 22.29 | ± 0.00 |
| **hsa-miR-3132** | UGGGUAGAGAAGGAGCUCAGAGGA | 24.93 | ± 1.72 | 31.92 | ± 1.70 |
| **hsa-miR-3133** | UAAAGAACUCUUAAAACCCAAU | 25.01 | ± 2.56 | 33.84 | ± 1.16 |
| **hsa-miR-3134** | UGAUGGAUAAAAGACUACAUAUU | 26.84 | ± 1.95 | 27.80 | ± 4.79 |
| **hsa-miR-3135** | UGCCUAGGCUGAGACUGCAGUG | 28.38 | ± 0.10 | 31.09 | ± 8.35 |
| **hsa-miR-3136** | CUGACUGAAUAGGUAGGGUCAUU | 32.35 | ± 6.93 | 31.38 | ± 1.95 |
| **hsa-miR-3137** | UCUGUAGCCUGGGAGCAAUGGGGU | 27.96 | ± 3.26 | 29.21 | ± 0.55 |
| **hsa-miR-3138** | UGUGGACAGUGAGGUAGAGGGAGU | 22.22 | ± 3.34 | 23.88 | ± 1.01 |
| **hsa-miR-3139** | UAGGAGCUCAACAGAUGCCUGUU | 40.00 | ± 4.79 | 24.88 | ± 0.11 |
| **hsa-miR-3140** | AGCUUUUGGGAAUUCAGGUAGU | 26.93 | ± 4.35 | 34.93 | ± 1.01 |
| **hsa-miR-3141** | GAGGGCGGGUGGAGGAGGA | 24.92 | ± 1.55 | 27.67 | ± 1.27 |
| **hsa-miR-3142** | AAGGCCUUUCUGAACCUUCAGA | 24.78 | ± 2.52 | 31.08 | ± 4.40 |
| **hsa-miR-3143** | AUAACAUUGUAAAGCGCUUCUUUCG | 25.84 | ± 3.07 | 27.60 | ± 0.63 |
| **hsa-miR-3144-5p** | AGGGGACCAAAGAGAUAUAUAG | 24.78 | ± 2.40 | 27.31 | ± 1.76 |
| **hsa-miR-3144-3p** | AUAUACCUGUUCGGUCUCUUUA | 28.84 | ± 2.40 | 35.60 | ± 2.26 |
| **hsa-miR-3145** | AGAUAUUUUGAGUGUUUGGAAUUG | 28.27 | ± 3.26 | 28.19 | ± 3.42 |
| **hsa-miR-3146** | CAUGCUAGGAUAGAAAGAAUGG | 24.71 | ± 3.34 | 38.96 | ± 1.78 |
| **hsa-miR-3147** | GGUUGGGCAGUGAGGAGGGUGUGA | 27.43 | ± 2.07 | 29.24 | ± 0.39 |
| **hsa-miR-3148** | UGGAAAAAACUGGUGUGUGCUU | 29.68 | ± 4.73 | 28.43 | ± 3.21 |
| **hsa-miR-3149** | UUUGUAUGGAUAUGUGUGUGUAU | 29.74 | ± 1.13 | 33.00 | ± 0.42 |
| **hsa-miR-3150** | CUGGGGAGAUCCUCGAGGUUGG | 29.28 | ± 0.67 | 30.92 | ± 6.33 |
| **hsa-miR-3151** | GGUGGGGCAAUGGGAUCAGGU | 27.48 | ± 1.13 | 26.69 | ± 0.32 |
| **hsa-miR-3152** | UGUGUUAGAAUAGGGGCAAUAA | 26.34 | ± 1.14 | 27.38 | ± 6.68 |
| **hsa-miR-3153** | GGGGAAAGCGAGUAGGGACAUUU | 29.90 | ± 0.29 | 34.39 | ± 2.21 |
| **hsa-miR-3154** | CAGAAGGGGAGUUGGGAGCAGA | 24.61 | ± 1.47 | 26.14 | ± 8.64 |
| **hsa-miR-3155** | CCAGGCUCUGCAGUGGGAACU | 27.74 | ± 3.24 | 29.73 | ± 2.70 |
| **hsa-miR-3156** | AAAGAUCUGGAAGUGGGAGACA | 24.73 | ± 1.25 | 26.16 | ± 0.49 |
| **hsa-miR-3157** | UUCAGCCAGGCUAGUGCAGUCU | 28.76 | ± 0.11 | 31.28 | ± 0.32 |
| **hsa-miR-3158** | AAGGGCUUCCUCUCUGCAGGAC | 27.78 | ± 0.97 | 28.99 | ± 0.33 |
| **hsa-miR-3159** | UAGGAUUACAAGUGUCGGCCAC | 23.09 | ± 3.32 | 24.63 | ± 0.53 |
| **hsa-miR-3160** | AGAGCUGAGACUAGAAAGCCCA | 27.21 | ± 3.02 | 28.51 | ± 1.43 |
| **hsa-miR-3161** | CUGAUAAGAACAGAGGCCCAGAU | 28.30 | ± 1.14 | 31.48 | ± 1.26 |
| **hsa-miR-3162** | UUAGGGAGUAGAAGGGUGGGGAG | 25.58 | ± 3.02 | 26.99 | ± 1.43 |
| **hsa-miR-3163** | UAUAAAAUGAGGGCAGUAAGAC | 31.56 | ± 1.13 | 22.82 | ± 1.29 |
| **hsa-miR-3164** | UGUGACUUUAAGGGAAAUGGCG | 27.46 | ± 1.77 | 29.41 | ± 1.34 |
| **hsa-miR-3165** | AGGUGGAUGCAAUGUGACCUCA | 27.66 | ± 2.79 | 28.14 | ± 1.60 |
| **hsa-miR-3166** | CGCAGACAAUGCCUACUGGCCUA | 29.36 | ± 1.59 | 30.03 | ± 0.87 |
| **hsa-miR-3167** | AGGAUUUCAGAAAUACUGGUGU | 29.84 | ± 2.01 | 31.21 | ± 1.56 |
| **hsa-miR-3168** | GAGUUCUACAGUCAGAC | 31.56 | ± 2.09 | 31.36 | ± 2.01 |
| **hsa-miR-3169** | UAGGACUGUGCUUGGCACAUAG | 30.58 | ± 6.02 | 35.71 | ± 2.09 |
| **hsa-miR-3170** | CUGGGGUUCUGAGACAGACAGU | 26.96 | ± 1.26 | 26.37 | ± 3.68 |
| **hsa-miR-3171** | AGAUGUAUGGAAUCUGUAUAUAUC | 31.56 | ± 1.57 | 33.51 | ± 0.89 |
| **hsa-miR-3172** | UGGGGUUUUGCAGUCCUUA | 23.37 | ± 1.24 | 25.11 | ± 2.44 |
| **hsa-miR-3173** | AAAGGAGGAAAUAGGCAGGCCA | 22.55 | ± 2.92 | 25.07 | ± 1.16 |
| **hsa-miR-3174** | UAGUGAGUUAGAGAUGCAGAGCC | 26.95 | ± 4.74 | 28.72 | ± 4.20 |
| **hsa-miR-3175** | CGGGGAGAGAACGCAGUGACGU | 29.15 | ± 3.45 | 33.18 | ± 0.06 |
| **hsa-miR-3176** | ACUGGCCUGGGACUACCGG | 25.30 | ± 3.37 | 26.67 | ± 5.53 |
| **hsa-miR-3177** | UGCACGGCACUGGGGACACGU | 27.77 | ± 0.59 | 30.57 | ± 6.47 |
| **hsa-miR-3178** | GGGGCGCGGCCGGAUCG | 27.31 | ± 1.97 | 28.25 | ± 2.43 |
| **hsa-miR-3179** | AGAAGGGGUGAAAUUUAAACGU | 28.94 | ± 1.57 | 35.19 | ± 1.96 |
| **hsa-miR-3180-5p** | CUUCCAGACGCUCCGCCCCACGUCG | 18.48 | ± 3.71 | 22.34 | ± 5.08 |
| **hsa-miR-3180-3p** | UGGGGCGGAGCUUCCGGAGGCC | 24.58 | ± 0.68 | 27.24 | ± 1.87 |
| **hsa-miR-3181** | AUCGGGCCCUCGGCGCCGG | 30.11 | ± 8.64 | 26.18 | ± 4.12 |
| **hsa-miR-3182** | GCUUCUGUAGUGUAGUC | 27.91 | ± 6.64 | 26.13 | ± 2.36 |
| **hsa-miR-3183** | GCCUCUCUCGGAGUCGCUCGGA | 27.48 | ± 2.10 | 29.26 | ± 1.74 |
| **hsa-miR-3184** | UGAGGGGCCUCAGACCGAGCUUUU | 31.56 | ± 0.60 | 29.47 | ± 0.41 |
| **hsa-miR-3185** | AGAAGAAGGCGGUCGGUCUGCGG | 28.25 | ± 0.90 | 28.68 | ± 1.70 |
| **hsa-miR-3186-5p** | CAGGCGUCUGUCUACGUGGCUU | 34.72 | ± 7.47 | 31.97 | ± 1.00 |
| **hsa-miR-3186-3p** | UCACGCGGAGAGAUGGCUUUG | 30.05 | ± 1.71 | 30.79 | ± 0.95 |
| **hsa-miR-3187** | UUGGCCAUGGGGCUGCGCGG | 27.23 | ± 0.69 | 28.63 | ± 2.37 |
| **hsa-miR-3188** | AGAGGCUUUGUGCGGAUACGGGG | 27.50 | ± 3.75 | 30.31 | ± 2.67 |
| **hsa-miR-3189** | CCCUUGGGUCUGAUGGGGUAG | 30.06 | ± 1.71 | 33.09 | ± 0.44 |
| **hsa-miR-3190-5p** | UGUGGAAGGUAGACGGCCAGAGA | 29.10 | ± 0.89 | 31.84 | ± 1.78 |
| **hsa-miR-3190-3p** | UGGAAGGUAGACGGCCAGAGAG | 28.00 | ± 1.03 | 30.88 | ± 1.41 |
| **hsa-miR-3191** | UGGGGACGUAGCUGGCCAGACAG | 28.29 | ± 2.06 | 30.14 | ± 0.49 |
| **hsa-miR-3192** | UCUGGGAGGUUGUAGCAGUGGAA | 27.02 | ± 2.62 | 28.71 | ± 4.34 |
| **hsa-miR-3193** | UCCUGCGUAGGAUCUGAGGAGU | 29.14 | ± 0.09 | 30.92 | ± 0.90 |
| **hsa-miR-3194** | GGCCAGCCACCAGGAGGGCUG | 38.17 | ± 1.61 | 37.63 | ± 1.65 |
| **hsa-miR-3195** | CGCGCCGGGCCCGGGUU | 25.85 | ± 2.55 | 26.70 | ± 1.53 |
| **hsa-miR-3196** | CGGGGCGGCAGGGGCCUC | 26.63 | ± 1.19 | 27.11 | ± 4.55 |
| **hsa-miR-3197** | GGAGGCGCAGGCUCGGAAAGGCG | 26.86 | ± 0.42 | 30.58 | ± 5.25 |
| **hsa-miR-3198** | GUGGAGUCCUGGGGAAUGGAGA | 30.29 | ± 1.22 | 32.69 | ± 3.91 |
| **hsa-miR-3199** | AGGGACUGCCUUAGGAGAAAGUU | 31.56 | ± 0.60 | 23.83 | ± 0.73 |
| **hsa-miR-3200** | CACCUUGCGCUACUCAGGUCUG | 28.00 | ± 3.12 | 25.98 | ± 1.91 |
| **hsa-miR-3201** | GGGAUAUGAAGAAAAAU | 26.15 | ± 0.44 | 15.98 | ± 2.06 |
| **hsa-miR-3202** | UGGAAGGGAGAAGAGCUUUAAU | 23.53 | ± 0.60 | 31.32 | ± 0.44 |
| **hsa-miR-320e** | AAAGCUGGGUUGAGAAGG | 28.03 | ± 0.64 | 28.64 | ± 1.29 |
| **hsa-miR-323b-5p** | AGGUUGUCCGUGGUGAGUUCGCA | 25.01 | ± 2.44 | 27.03 | ± 0.21 |
| **hsa-miR-378b** | ACUGGACUUGGAGGCAGAA | 28.05 | ± 1.13 | 31.73 | ± 0.62 |
| **hsa-miR-378c** | ACUGGACUUGGAGUCAGAAGAGUGG | 29.51 | ± 1.39 | 27.08 | ± 1.22 |
| **hsa-miR-4251** | CCUGAGAAAAGGGCCAA | 26.08 | ± 0.04 | 27.10 | ± 4.31 |
| **hsa-miR-4252** | GGCCACUGAGUCAGCACCA | 22.59 | ± 1.69 | 25.34 | ± 1.95 |
| **hsa-miR-4253** | AGGGCAUGUCCAGGGGGU | 28.59 | ± 0.55 | 29.58 | ± 1.54 |
| **hsa-miR-4254** | GCCUGGAGCUACUCCACCAUCUC | 26.22 | ± 0.90 | 29.47 | ± 1.67 |
| **hsa-miR-4255** | CAGUGUUCAGAGAUGGA | 28.42 | ± 3.00 | 27.59 | ± 0.85 |
| **hsa-miR-4256** | AUCUGACCUGAUGAAGGU | 27.34 | ± 0.68 | 34.70 | ± 3.00 |
| **hsa-miR-4257** | CCAGAGGUGGGGACUGAG | 28.02 | ± 0.42 | 32.46 | ± 2.12 |
| **hsa-miR-4258** | CCCCGCCACCGCCUUGG | 27.94 | ± 0.09 | 29.84 | ± 1.23 |
| **hsa-miR-4259** | CAGUUGGGUCUAGGGGUCAGGA | 26.73 | ± 2.15 | 28.16 | ± 0.98 |
| **hsa-miR-4260** | CUUGGGGCAUGGAGUCCCA | 40.00 | ± 0.00 | 34.66 | ± 0.00 |
| **hsa-miR-4261** | AGGAAACAGGGACCCA | 27.40 | ± 1.24 | 28.60 | ± 1.11 |
| **hsa-miR-4262** | GACAUUCAGACUACCUG | 25.84 | ± 1.72 | 33.66 | ± 0.19 |
| **hsa-miR-4263** | AUUCUAAGUGCCUUGGCC | 28.05 | ± 0.66 | 29.70 | ± 1.03 |
| **hsa-miR-4264** | ACUCAGUCAUGGUCAUU | 28.36 | ± 0.63 | 30.65 | ± 1.22 |
| **hsa-miR-4265** | CUGUGGGCUCAGCUCUGGG | 24.84 | ± 0.13 | 31.04 | ± 0.52 |
| **hsa-miR-4266** | CUAGGAGGCCUUGGCC | 27.74 | ± 0.67 | 23.43 | ± 2.48 |
| **hsa-miR-4267** | UCCAGCUCGGUGGCAC | 22.34 | ± 4.05 | 24.06 | ± 4.78 |
| **hsa-miR-4268** | GGCUCCUCCUCUCAGGAUGUG | 25.51 | ± 1.62 | 36.01 | ± 3.20 |
| **hsa-miR-4269** | GCAGGCACAGACAGCCCUGGC | 25.79 | ± 3.77 | 27.85 | ± 0.83 |
| **hsa-miR-4270** | UCAGGGAGUCAGGGGAGGGC | 31.26 | ± 7.57 | 26.35 | ± 0.75 |
| **hsa-miR-4271** | GGGGGAAGAAAAGGUGGGG | 26.81 | ± 2.75 | 28.27 | ± 3.77 |
| **hsa-miR-4272** | CAUUCAACUAGUGAUUGU | 29.48 | ± 0.76 | 29.76 | ± 1.73 |
| **hsa-miR-4273** | GUGUUCUCUGAUGGACAG | 40.00 | ± 0.42 | 27.09 | ± 1.89 |
| **hsa-miR-4274** | CAGCAGUCCCUCCCCCUG | 23.41 | ± 3.57 | 25.04 | ± 0.71 |
| **hsa-miR-4275** | CCAAUUACCACUUCUUU | 31.89 | ± 0.50 | 23.48 | ± 0.42 |
| **hsa-miR-4276** | CUCAGUGACUCAUGUGC | 26.45 | ± 4.78 | 28.46 | ± 4.19 |
| **hsa-miR-4277** | GCAGUUCUGAGCACAGUACAC | 31.39 | ± 0.50 | 32.86 | ± 0.50 |
| **hsa-miR-4278** | CUAGGGGGUUUGCCCUUG | 26.69 | ± 0.90 | 28.13 | ± 4.51 |
| **hsa-miR-4279** | CUCUCCUCCCGGCUUC | 26.77 | ± 2.23 | 29.35 | ± 2.76 |
| **hsa-miR-4280** | GAGUGUAGUUCUGAGCAGAGC | 28.60 | ± 2.97 | 31.95 | ± 7.31 |
| **hsa-miR-4281** | GGGUCCCGGGGAGGGGGG | 26.09 | ± 2.08 | 27.39 | ± 0.42 |
| **hsa-miR-4282** | UAAAAUUUGCAUCCAGGA | 32.18 | ± 2.84 | 35.52 | ± 5.46 |
| **hsa-miR-4283** | UGGGGCUCAGCGAGUUU | 29.25 | ± 1.89 | 35.32 | ± 1.93 |
| **hsa-miR-4283** | UGGGGCUCAGCGAGUUU | 31.10 | ± 0.36 | 33.46 | ± 3.99 |
| **hsa-miR-4284** | GGGCUCACAUCACCCCAU | 25.66 | ± 3.42 | 27.18 | ± 1.43 |
| **hsa-miR-4285** | GCGGCGAGUCCGACUCAU | 30.66 | ± 1.93 | 34.65 | ± 1.72 |
| **hsa-miR-4286** | ACCCCACUCCUGGUACC | 32.69 | ± 1.65 | 23.72 | ± 5.30 |
| **hsa-miR-4287** | UCUCCCUUGAGGGCACUUU | 26.27 | ± 0.50 | 30.69 | ± 3.45 |
| **hsa-miR-4288** | UUGUCUGCUGAGUUUCC | 30.21 | ± 0.73 | 22.90 | ± 1.45 |
| **hsa-miR-4289** | GCAUUGUGCAGGGCUAUCA | 31.22 | ± 1.68 | 30.47 | ± 0.92 |
| **hsa-miR-4290** | UGCCCUCCUUUCUUCCCUC | 34.60 | ± 2.64 | 29.69 | ± 0.94 |
| **hsa-miR-4291** | UUCAGCAGGAACAGCU | 29.25 | ± 1.66 | 27.45 | ± 1.89 |
| **hsa-miR-4292** | CCCCUGGGCCGGCCUUGG | 23.26 | ± 2.00 | 25.05 | ± 0.78 |
| **hsa-miR-4293** | CAGCCUGACAGGAACAG | 29.48 | ± 0.73 | 29.03 | ± 0.78 |
| **hsa-miR-4294** | GGGAGUCUACAGCAGGG | 31.43 | ± 7.92 | 29.11 | ± 1.05 |
| **hsa-miR-4295** | CAGUGCAAUGUUUUCCUU | 29.25 | ± 1.34 | 24.77 | ± 1.93 |
| **hsa-miR-4296** | AUGUGGGCUCAGGCUCA | 34.51 | ± 9.50 | 28.66 | ± 1.48 |
| **hsa-miR-4297** | UGCCUUCCUGUCUGUG | 29.63 | ± 9.47 | 27.64 | ± 0.21 |
| **hsa-miR-4298** | CUGGGACAGGAGGAGGAGGCAG | 31.24 | ± 2.39 | 25.34 | ± 1.07 |
| **hsa-miR-4299** | GCUGGUGACAUGAGAGGC | 40.00 | ± 0.00 | 31.65 | ± 0.38 |
| **hsa-miR-4300** | UGGGAGCUGGACUACUUC | 28.58 | ± 4.98 | 33.03 | ± 0.12 |
| **hsa-miR-4301** | UCCCACUACUUCACUUGUGA | 22.56 | ± 3.25 | 35.30 | ± 2.43 |
| **hsa-miR-4302** | CCAGUGUGGCUCAGCGAG | 26.92 | ± 3.74 | 29.99 | ± 2.34 |
| **hsa-miR-4303** | UUCUGAGCUGAGGACAG | 23.14 | ± 1.93 | 27.61 | ± 3.43 |
| **hsa-miR-4304** | CCGGCAUGUCCAGGGCA | 35.21 | ± 3.30 | 28.87 | ± 2.34 |
| **hsa-miR-4305** | CCUAGACACCUCCAGUUC | 30.42 | ± 1.72 | 33.63 | ± 1.13 |
| **hsa-miR-4306** | UGGAGAGAAAGGCAGUA | 21.01 | ± 2.88 | 32.67 | ± 1.99 |
| **hsa-miR-4307** | AAUGUUUUUUCCUGUUUCC | 28.02 | ± 2.25 | 33.86 | ± 1.89 |
| **hsa-miR-4308** | UCCCUGGAGUUUCUUCUU | 34.21 | ± 2.84 | 36.02 | ± 4.19 |
| **hsa-miR-4309** | CUGGAGUCUAGGAUUCCA | 25.51 | ± 4.97 | 28.00 | ± 1.07 |
| **hsa-miR-4310** | GCAGCAUUCAUGUCCC | 28.64 | ± 1.69 | 28.65 | ± 1.55 |
| **hsa-miR-4311** | GAAAGAGAGCUGAGUGUG | 25.00 | ± 0.60 | 28.52 | ± 3.06 |
| **hsa-miR-4312** | GGCCUUGUUCCUGUCCCCA | 24.33 | ± 4.44 | 25.36 | ± 0.81 |
| **hsa-miR-4313** | AGCCCCCUGGCCCCAAACCC | 23.23 | ± 0.57 | 26.95 | ± 0.44 |
| **hsa-miR-4314** | CUCUGGGAAAUGGGACAG | 28.29 | ± 2.47 | 31.44 | ± 3.94 |
| **hsa-miR-4315** | CCGCUUUCUGAGCUGGAC | 28.12 | ± 0.69 | 32.22 | ± 4.94 |
| **hsa-miR-4315** | CCGCUUUCUGAGCUGGAC | 32.29 | ± 4.90 | 27.04 | ± 1.56 |
| **hsa-miR-4316** | GGUGAGGCUAGCUGGUG | 28.02 | ± 3.80 | 29.12 | ± 3.34 |
| **hsa-miR-4317** | ACAUUGCCAGGGAGUUU | 25.31 | ± 3.80 | 28.07 | ± 1.77 |
| **hsa-miR-4318** | CACUGUGGGUACAUGCU | 27.88 | ± 0.38 | 29.56 | ± 2.80 |
| **hsa-miR-4319** | UCCCUGAGCAAAGCCAC | 28.53 | ± 3.35 | 30.68 | ± 1.84 |
| **hsa-miR-4320** | GGGAUUCUGUAGCUUCCU | 28.08 | ± 2.17 | 26.84 | ± 1.77 |
| **hsa-miR-4321** | UUAGCGGUGGACCGCCCUGCG | 29.73 | ± 0.42 | 31.39 | ± 2.19 |
| **hsa-miR-4322** | CUGUGGGCUCAGCGCGUGGGG | 21.34 | ± 2.30 | 25.84 | ± 2.43 |
| **hsa-miR-4323** | CAGCCCCACAGCCUCAGA | 36.03 | ± 6.87 | 29.76 | ± 3.16 |
| **hsa-miR-4324** | CCCUGAGACCCUAACCUUAA | 28.75 | ± 1.64 | 29.57 | ± 4.82 |
| **hsa-miR-4325** | UUGCACUUGUCUCAGUGA | 24.01 | ± 2.17 | 30.62 | ± 2.73 |
| **hsa-miR-4326** | UGUUCCUCUGUCUCCCAGAC | 24.36 | ± 0.42 | 28.18 | ± 3.12 |
| **hsa-miR-4327** | GGCUUGCAUGGGGGACUGG | 31.76 | ± 4.27 | 25.44 | ± 4.90 |
| **hsa-miR-4328** | CCAGUUUUCCCAGGAUU | 29.01 | ± 1.40 | 35.45 | ± 0.78 |
| **hsa-miR-4329** | CCUGAGACCCUAGUUCCAC | 32.54 | ± 4.22 | 35.04 | ± 1.34 |
| **hsa-miR-4330** | CCUCAGAUCAGAGCCUUGC | 28.33 | ± 2.33 | 31.38 | ± 2.63 |
| **hsa-miR-466** | AUACACAUACACGCAACACACAU | 28.25 | ± 1.59 | 30.75 | ± 5.04 |
| **hsa-let-7a*** | CUAUACAAUCUACUGUCUUUC | 27.34 | ± 0.23 | 22.77 | ± 1.43 |
| **hsa-let-7a-2*** | CUGUACAGCCUCCUAGCUUUCC | 25.60 | ± 1.88 | 29.10 | ± 1.53 |
| **hsa-let-7b*** | CUAUACAACCUACUGCCUUCCC | 24.43 | ± 2.49 | 27.31 | ± 1.15 |
| **hsa-let-7c*** | UAGAGUUACACCCUGGGAGUUA | 25.71 | ± 0.26 | 20.40 | ± 0.96 |
| **hsa-let-7d*** | CUAUACGACCUGCUGCCUUUCU | 24.78 | ± 1.36 | 27.62 | ± 0.38 |
| **hsa-let-7e*** | CUAUACGGCCUCCUAGCUUUCC | 24.29 | ± 3.69 | 25.33 | ± 4.56 |
| **hsa-let-7f-1*** | CUAUACAAUCUAUUGCCUUCCC | 25.96 | ± 1.94 | 28.72 | ± 7.86 |
| **hsa-let-7f-2*** | CUAUACAGUCUACUGUCUUUCC | 31.47 | ± 1.17 | 33.29 | ± 1.68 |
| **hsa-let-7g*** | CUGUACAGGCCACUGCCUUGC | 29.30 | ± 1.21 | 31.43 | ± 3.04 |
| **hsa-let-7i*** | CUGCGCAAGCUACUGCCUUGCU | 25.80 | ± 1.17 | 34.82 | ± 5.74 |
| **hsa-miR-7-1*** | CAACAAAUCACAGUCUGCCAUA | 24.90 | ± 2.45 | 26.80 | ± 5.05 |
| **hsa-miR-7-2*** | CAACAAAUCCCAGUCUACCUAA | 24.33 | ± 5.88 | 25.89 | ± 2.31 |
| **hsa-miR-9*** | AUAAAGCUAGAUAACCGAAAGU | 26.73 | ± 1.02 | 29.77 | ± 1.48 |
| **hsa-miR-10a*** | CAAAUUCGUAUCUAGGGGAAUA | 23.14 | ± 1.68 | 30.04 | ± 2.70 |
| **hsa-miR-10b*** | ACAGAUUCGAUUCUAGGGGAAU | 23.47 | ± 1.47 | 28.08 | ± 1.88 |
| **hsa-miR-15a*** | CAGGCCAUAUUGUGCUGCCUCA | 27.87 | ± 0.72 | 26.13 | ± 4.62 |
| **hsa-miR-15b*** | CGAAUCAUUAUUUGCUGCUCUA | 26.93 | ± 0.82 | 23.40 | ± 4.26 |
| **hsa-miR-16-1*** | CCAGUAUUAACUGUGCUGCUGA | 24.73 | ± 1.38 | 27.82 | ± 3.67 |
| **hsa-miR-16-2*** | CCAAUAUUACUGUGCUGCUUUA | 26.15 | ± 4.25 | 26.84 | ± 1.20 |
| **hsa-miR-17*** | ACUGCAGUGAAGGCACUUGUAG | 28.10 | ± 1.17 | 25.07 | ± 2.33 |
| **hsa-miR-18a*** | ACUGCCCUAAGUGCUCCUUCUGG | 32.61 | ± 1.45 | 30.19 | ± 5.33 |
| **hsa-miR-18b*** | UGCCCUAAAUGCCCCUUCUGGC | 28.58 | ± 3.14 | 31.61 | ± 4.10 |
| **hsa-miR-19a*** | AGUUUUGCAUAGUUGCACUACA | 30.25 | ± 4.55 | 32.20 | ± 2.49 |
| **hsa-miR-19b-1*** | AGUUUUGCAGGUUUGCAUCCAGC | 29.78 | ± 3.96 | 30.56 | ± 1.45 |
| **hsa-miR-19b-2*** | AGUUUUGCAGGUUUGCAUUUCA | 26.53 | ± 3.99 | 31.62 | ± 0.32 |
| **hsa-miR-20a*** | ACUGCAUUAUGAGCACUUAAAG | 30.45 | ± 1.42 | 33.70 | ± 1.43 |
| **hsa-miR-20b*** | ACUGUAGUAUGGGCACUUCCAG | 22.21 | ± 1.19 | 25.12 | ± 4.59 |
| **hsa-miR-21*** | CAACACCAGUCGAUGGGCUGU | 27.90 | ± 0.59 | 29.29 | ± 1.30 |
| **hsa-miR-22*** | AGUUCUUCAGUGGCAAGCUUUA | 23.49 | ± 2.56 | 26.83 | ± 0.36 |
| **hsa-miR-23a*** | GGGGUUCCUGGGGAUGGGAUUU | 29.07 | ± 0.64 | 31.06 | ± 0.64 |
| **hsa-miR-23b*** | UGGGUUCCUGGCAUGCUGAUUU | 28.58 | ± 0.78 | 32.27 | ± 0.78 |
| **hsa-miR-24-1*** | UGCCUACUGAGCUGAUAUCAGU | 40.00 | ± 3.02 | 32.34 | ± 2.50 |
| **hsa-miR-24-2*** | UGCCUACUGAGCUGAAACACAG | 28.76 | ± 1.21 | 27.78 | ± 0.32 |
| **hsa-miR-25*** | AGGCGGAGACUUGGGCAAUUG | 24.79 | ± 4.10 | 30.64 | ± 5.01 |
| **hsa-miR-26a-1*** | CCUAUUCUUGGUUACUUGCACG | 27.52 | ± 5.47 | 31.94 | ± 4.02 |
| **hsa-miR-26a-2*** | CCUAUUCUUGAUUACUUGUUUC | 27.37 | ± 3.01 | 25.01 | ± 3.02 |
| **hsa-miR-26b*** | CCUGUUCUCCAUUACUUGGCUC | 27.56 | ± 3.17 | 26.02 | ± 0.54 |
| **hsa-miR-27a*** | AGGGCUUAGCUGCUUGUGAGCA | 40.00 | ± 0.00 | 3.87 | ± 0.00 |
| **hsa-miR-27b*** | AGAGCUUAGCUGAUUGGUGAAC | 25.33 | ± 0.49 | 30.47 | ± 1.29 |
| **hsa-miR-29a*** | ACUGAUUUCUUUUGGUGUUCAG | 27.03 | ± 2.19 | 29.81 | ± 3.02 |
| **hsa-miR-29b-1*** | GCUGGUUUCAUAUGGUGGUUUAGA | 28.32 | ± 1.47 | 28.51 | ± 2.48 |
| **hsa-miR-29b-2*** | CUGGUUUCACAUGGUGGCUUAG | 26.93 | ± 2.51 | 20.41 | ± 2.19 |
| **hsa-miR-29c*** | UGACCGAUUUCUCCUGGUGUUC | 27.37 | ± 2.37 | 32.40 | ± 1.47 |
| **hsa-miR-30a*** | CUUUCAGUCGGAUGUUUGCAGC | 29.16 | ± 9.39 | 26.32 | ± 1.01 |
| **hsa-miR-30b*** | CUGGGAGGUGGAUGUUUACUUC | 29.61 | ± 2.42 | 32.10 | ± 2.51 |
| **hsa-miR-30c-1*** | CUGGGAGAGGGUUGUUUACUCC | 27.60 | ± 7.03 | 31.98 | ± 0.71 |
| **hsa-miR-30c-2*** | CUGGGAGAAGGCUGUUUACUCU | 26.39 | ± 5.03 | 33.54 | ± 1.43 |
| **hsa-miR-30d*** | CUUUCAGUCAGAUGUUUGCUGC | 25.38 | ± 3.28 | 32.77 | ± 2.61 |
| **hsa-miR-30e*** | CUUUCAGUCGGAUGUUUACAGC | 26.75 | ± 4.11 | 28.95 | ± 0.47 |
| **hsa-miR-31*** | UGCUAUGCCAACAUAUUGCCAU | 27.92 | ± 1.61 | 32.25 | ± 1.21 |
| **hsa-miR-32*** | CAAUUUAGUGUGUGUGAUAUUU | 25.78 | ± 2.64 | 31.97 | ± 1.85 |
| **hsa-miR-33a*** | CAAUGUUUCCACAGUGCAUCAC | 26.43 | ± 3.09 | 29.94 | ± 3.18 |
| **hsa-miR-33b*** | CAGUGCCUCGGCAGUGCAGCCC | 23.51 | ± 3.06 | 24.67 | ± 0.69 |
| **hsa-miR-34a*** | CAAUCAGCAAGUAUACUGCCCU | 27.25 | ± 5.10 | 29.52 | ± 6.07 |
| **hsa-miR-34b*** | UAGGCAGUGUCAUUAGCUGAUUG | 34.57 | ± 1.43 | 24.52 | ± 2.64 |
| **hsa-miR-92a-1*** | AGGUUGGGAUCGGUUGCAAUGCU | 25.40 | ± 6.36 | 28.20 | ± 1.82 |
| **hsa-miR-92a-2*** | GGGUGGGGAUUUGUUGCAUUAC | 30.31 | ± 0.20 | 31.27 | ± 1.71 |
| **hsa-miR-92b*** | AGGGACGGGACGCGGUGCAGUG | 24.45 | ± 5.61 | 28.11 | ± 3.44 |
| **hsa-miR-93*** | ACUGCUGAGCUAGCACUUCCCG | 26.24 | ± 5.75 | 30.89 | ± 1.65 |
| **hsa-miR-96*** | AAUCAUGUGCAGUGCCAAUAUG | 30.26 | ± 2.51 | 33.54 | ± 1.71 |
| **hsa-miR-99a*** | CAAGCUCGCUUCUAUGGGUCUG | 22.31 | ± 3.51 | 31.43 | ± 2.25 |
| **hsa-miR-99b*** | CAAGCUCGUGUCUGUGGGUCCG | 21.02 | ± 4.40 | 23.93 | ± 0.88 |
| **hsa-miR-100*** | CAAGCUUGUAUCUAUAGGUAUG | 24.50 | ± 0.38 | 21.81 | ± 2.39 |
| **hsa-miR-101*** | CAGUUAUCACAGUGCUGAUGCU | 23.92 | ± 1.69 | 20.93 | ± 3.50 |
| **hsa-miR-103-2*** | AGCUUCUUUACAGUGCUGCCUUG | 33.32 | ± 9.44 | 30.15 | ± 1.61 |
| **hsa-miR-105*** | ACGGAUGUUUGAGCAUGUGCUA | 27.98 | ± 2.82 | 31.44 | ± 1.79 |
| **hsa-miR-106a*** | CUGCAAUGUAAGCACUUCUUAC | 29.81 | ± 1.79 | 28.44 | ± 1.69 |
| **hsa-miR-106b*** | CCGCACUGUGGGUACUUGCUGC | 34.59 | ± 7.64 | 30.39 | ± 0.93 |
| **hsa-miR-122*** | AACGCCAUUAUCACACUAAAUA | 27.16 | ± 1.69 | 34.22 | ± 2.60 |
| **hsa-miR-124*** | CGUGUUCACAGCGGACCUUGAU | 29.79 | ± 1.90 | 29.61 | ± 1.79 |
| **hsa-miR-125b-1*** | ACGGGUUAGGCUCUUGGGAGCU | 28.55 | ± 1.42 | 32.55 | ± 5.95 |
| **hsa-miR-125b-2*** | UCACAAGUCAGGCUCUUGGGAC | 28.30 | ± 0.38 | 31.90 | ± 1.03 |
| **hsa-miR-126*** | CAUUAUUACUUUUGGUACGCG | 23.77 | ± 2.27 | 25.46 | ± 0.63 |
| **hsa-miR-129*** | AAGCCCUUACCCCAAAAAGUAU | 27.88 | ± 3.62 | 27.13 | ± 0.50 |
| **hsa-miR-130a*** | UUCACAUUGUGCUACUGUCUGC | 26.83 | ± 3.03 | 29.42 | ± 6.71 |
| **hsa-miR-130b*** | ACUCUUUCCCUGUUGCACUAC | 25.06 | ± 1.17 | 27.01 | ± 3.02 |
| **hsa-miR-132*** | ACCGUGGCUUUCGAUUGUUACU | 25.26 | ± 0.38 | 23.85 | ± 3.21 |
| **hsa-miR-135a*** | UAUAGGGAUUGGAGCCGUGGCG | 26.58 | ± 1.34 | 27.82 | ± 0.84 |
| **hsa-miR-135b*** | AUGUAGGGCUAAAAGCCAUGGG | 29.00 | ± 0.44 | 33.64 | ± 5.74 |
| **hsa-miR-136*** | CAUCAUCGUCUCAAAUGAGUCU | 25.98 | ± 0.38 | 32.39 | ± 1.82 |
| **hsa-miR-138-1*** | GCUACUUCACAACACCAGGGCC | 25.76 | ± 1.75 | 28.02 | ± 1.60 |
| **hsa-miR-138-2*** | GCUAUUUCACGACACCAGGGUU | 26.46 | ± 1.83 | 30.38 | ± 2.81 |
| **hsa-miR-141*** | CAUCUUCCAGUACAGUGUUGGA | 27.06 | ± 2.13 | 28.15 | ± 4.01 |
| **hsa-miR-143*** | GGUGCAGUGCUGCAUCUCUGGU | 32.27 | ± 6.89 | 29.63 | ± 0.32 |
| **hsa-miR-144*** | GGAUAUCAUCAUAUACUGUAAG | 29.17 | ± 1.18 | 32.11 | ± 1.18 |
| **hsa-miR-145*** | GGAUUCCUGGAAAUACUGUUCU | 25.49 | ± 3.46 | 25.14 | ± 5.53 |
| **hsa-miR-146a*** | CCUCUGAAAUUCAGUUCUUCAG | 27.63 | ± 1.18 | 33.01 | ± 2.33 |
| **hsa-miR-148a*** | AAAGUUCUGAGACACUCCGACU | 28.22 | ± 1.99 | 29.13 | ± 6.16 |
| **hsa-miR-148b*** | AAGUUCUGUUAUACACUCAGGC | 27.35 | ± 2.01 | 31.90 | ± 2.11 |
| **hsa-miR-149*** | AGGGAGGGACGGGGGCUGUGC | 24.50 | ± 4.24 | 25.63 | ± 2.37 |
| **hsa-miR-150*** | CUGGUACAGGCCUGGGGGACAG | 26.02 | ± 2.24 | 27.58 | ± 2.48 |
| **hsa-miR-154*** | AAUCAUACACGGUUGACCUAUU | 27.47 | ± 3.84 | 33.42 | ± 2.86 |
| **hsa-miR-155*** | CUCCUACAUAUUAGCAUUAACA | 29.64 | ± 5.27 | 25.64 | ± 1.17 |
| **hsa-miR-181a*** | ACCAUCGACCGUUGAUUGUACC | 32.39 | ± 0.79 | 36.04 | ± 0.78 |
| **hsa-miR-181a-2*** | ACCACUGACCGUUGACUGUACC | 27.51 | ± 3.07 | 31.31 | ± 2.22 |
| **hsa-miR-181c*** | AACCAUCGACCGUUGAGUGGAC | 27.80 | ± 1.94 | 30.12 | ± 0.96 |
| **hsa-miR-182*** | UGGUUCUAGACUUGCCAACUA | 28.40 | ± 3.62 | 28.36 | ± 1.00 |
| **hsa-miR-183*** | GUGAAUUACCGAAGGGCCAUAA | 29.69 | ± 1.24 | 30.92 | ± 7.36 |
| **hsa-miR-185*** | AGGGGCUGGCUUUCCUCUGGUC | 24.86 | ± 6.18 | 26.98 | ± 1.11 |
| **hsa-miR-186*** | GCCCAAAGGUGAAUUUUUUGGG | 34.19 | ± 1.85 | 35.18 | ± 2.06 |
| **hsa-miR-187*** | GGCUACAACACAGGACCCGGGC | 24.96 | ± 3.46 | 22.19 | ± 1.83 |
| **hsa-miR-191*** | GCUGCGCUUGGAUUUCGUCCCC | 24.32 | ± 4.61 | 27.05 | ± 3.67 |
| **hsa-miR-192*** | CUGCCAAUUCCAUAGGUCACAG | 33.00 | ± 6.09 | 29.49 | ± 4.68 |
| **hsa-miR-193b*** | CGGGGUUUUGAGGGCGAGAUGA | 28.86 | ± 2.97 | 31.19 | ± 5.31 |
| **hsa-miR-194*** | CCAGUGGGGCUGCUGUUAUCUG | 29.69 | ± 3.00 | 33.00 | ± 2.06 |
| **hsa-miR-195*** | CCAAUAUUGGCUGUGCUGCUCC | 23.31 | ± 3.24 | 27.22 | ± 1.89 |
| **hsa-miR-196a*** | CGGCAACAAGAAACUGCCUGAG | 28.61 | ± 0.32 | 29.54 | ± 1.91 |
| **hsa-miR-196b*** | UCGACAGCACGACACUGCCUUC | 21.95 | ± 3.97 | 26.46 | ± 3.52 |
| **hsa-miR-200a*** | CAUCUUACCGGACAGUGCUGGA | 28.75 | ± 1.64 | 30.39 | ± 4.80 |
| **hsa-miR-200b*** | CAUCUUACUGGGCAGCAUUGGA | 27.57 | ± 2.56 | 25.99 | ± 2.04 |
| **hsa-miR-200c*** | CGUCUUACCCAGCAGUGUUUGG | 27.69 | ± 1.39 | 30.17 | ± 2.30 |
| **hsa-miR-202*** | UUCCUAUGCAUAUACUUCUUUG | 29.42 | ± 2.36 | 31.51 | ± 2.65 |
| **hsa-miR-205*** | GAUUUCAGUGGAGUGAAGUUC | 31.05 | ± 0.21 | 32.37 | ± 2.45 |
| **hsa-miR-214*** | UGCCUGUCUACACUUGCUGUGC | 25.74 | ± 9.31 | 27.57 | ± 5.07 |
| **hsa-miR-218-1*** | AUGGUUCCGUCAAGCACCAUGG | 30.24 | ± 1.77 | 28.96 | ± 2.58 |
| **hsa-miR-218-2*** | CAUGGUUCUGUCAAGCACCGCG | 18.71 | ± 6.96 | 22.64 | ± 2.52 |
| **hsa-miR-221*** | ACCUGGCAUACAAUGUAGAUUU | 28.37 | ± 1.77 | 31.55 | ± 1.77 |
| **hsa-miR-222*** | CUCAGUAGCCAGUGUAGAUCCU | 30.20 | ± 0.13 | 28.45 | ± 1.89 |
| **hsa-miR-223*** | CGUGUAUUUGACAAGCUGAGUU | 29.58 | ± 1.44 | 29.16 | ± 1.46 |
| **hsa-miR-224*** | AAAAUGGUGCCCUAGUGACUACA | 29.83 | ± 1.62 | 31.95 | ± 1.85 |
| **hsa-miR-302a*** | ACUUAAACGUGGAUGUACUUGCU | 28.97 | ± 2.65 | 32.08 | ± 1.77 |
| **hsa-miR-302b*** | ACUUUAACAUGGAAGUGCUUUC | 28.42 | ± 2.45 | 32.78 | ± 1.80 |
| **hsa-miR-302c*** | UUUAACAUGGGGGUACCUGCUG | 27.96 | ± 1.11 | 30.83 | ± 4.65 |
| **hsa-miR-302d*** | ACUUUAACAUGGAGGCACUUGC | 31.62 | ± 4.41 | 28.24 | ± 0.01 |
| **hsa-miR-335*** | UUUUUCAUUAUUGCUCCUGACC | 20.18 | ± 0.01 | 23.69 | ± 1.57 |
| **hsa-miR-340*** | UCCGUCUCAGUUACUUUAUAGC | 25.67 | ± 1.11 | 27.97 | ± 0.17 |
| **hsa-miR-363*** | CGGGUGGAUCACGAUGCAAUUU | 29.58 | ± 0.17 | 25.47 | ± 2.54 |
| **hsa-miR-365*** | AGGGACUUUCAGGGGCAGCUGU | 25.60 | ± 2.10 | 28.48 | ± 3.28 |
| **hsa-miR-367*** | ACUGUUGCUAAUAUGCAACUCU | 21.37 | ± 1.59 | 34.49 | ± 0.45 |
| **hsa-miR-373*** | ACUCAAAAUGGGGGCGCUUUCC | 23.88 | ± 1.59 | 27.06 | ± 1.22 |
| **hsa-miR-374a*** | CUUAUCAGAUUGUAUUGUAAUU | 28.88 | ± 2.32 | 35.71 | ± 2.32 |
| **hsa-miR-374b*** | CUUAGCAGGUUGUAUUAUCAUU | 24.62 | ± 1.45 | 30.45 | ± 1.45 |
| **hsa-miR-376a*** | GUAGAUUCUCCUUCUAUGAGUA | 23.51 | ± 1.81 | 31.13 | ± 1.81 |
| **hsa-miR-377*** | AGAGGUUGCCCUUGGUGAAUUC | 29.10 | ± 1.56 | 25.01 | ± 1.56 |
| **hsa-miR-378*** | CUCCUGACUCCAGGUCCUGUGU | 27.61 | ± 1.90 | 33.36 | ± 1.58 |
| **hsa-miR-379*** | UAUGUAACAUGGUCCACUAACU | 28.84 | ± 2.16 | 31.36 | ± 2.86 |
| **hsa-miR-380*** | UGGUUGACCAUAGAACAUGCGC | 21.91 | ± 11.31 | 27.69 | ± 3.22 |
| **hsa-miR-411*** | UAUGUAACACGGUCCACUAACC | 22.07 | ± 0.56 | 32.06 | ± 1.06 |
| **hsa-miR-424*** | CAAAACGUGAGGCGCUGCUAU | 25.42 | ± 2.09 | 29.29 | ± 2.91 |
| **hsa-miR-425*** | AUCGGGAAUGUCGUGUCCGCCC | 26.57 | ± 2.34 | 27.23 | ± 1.78 |
| **hsa-miR-431*** | CAGGUCGUCUUGCAGGGCUUCU | 24.06 | ± 6.40 | 25.78 | ± 1.54 |
| **hsa-miR-432*** | CUGGAUGGCUCCUCCAUGUCU | 30.86 | ± 1.69 | 31.24 | ± 1.56 |
| **hsa-miR-449b*** | CAGCCACAACUACCCUGCCACU | 22.51 | ± 2.61 | 24.11 | ± 0.84 |
| **hsa-miR-452*** | CUCAUCUGCAAAGAAGUAAGUG | 27.89 | ± 0.55 | 32.47 | ± 1.06 |
| **hsa-miR-454*** | ACCCUAUCAAUAUUGUCUCUGC | 26.55 | ± 2.25 | 31.15 | ± 0.70 |
| **hsa-miR-488*** | CCCAGAUAAUGGCACUCUCAA | 26.40 | ± 2.78 | 28.84 | ± 1.70 |
| **hsa-miR-493*** | UUGUACAUGGUAGGCUUUCAUU | 23.90 | ± 1.69 | 28.36 | ± 1.11 |
| **hsa-miR-497*** | CAAACCACACUGUGGUGUUAGA | 32.00 | ± 1.44 | 33.81 | ± 0.23 |
| **hsa-miR-500*** | AUGCACCUGGGCAAGGAUUCUG | 26.92 | ± 2.68 | 29.13 | ± 2.35 |
| **hsa-miR-505*** | GGGAGCCAGGAAGUAUUGAUGU | 40.00 | ± 1.32 | 26.56 | ± 1.58 |
| **hsa-miR-516b*** | UGCUUCCUUUCAGAGGGU | 27.32 | ± 0.47 | 29.23 | ± 0.70 |
| **hsa-miR-517*** | CCUCUAGAUGGAAGCACUGUCU | 26.33 | ± 2.57 | 29.84 | ± 1.46 |
| **hsa-miR-518c*** | UCUCUGGAGGGAAGCACUUUCUG | 26.31 | ± 3.89 | 29.83 | ± 1.90 |
| **hsa-miR-518e*** | CUCUAGAGGGAAGCGCUUUCUG | 27.44 | ± 0.20 | 29.12 | ± 0.82 |
| **hsa-miR-518f*** | CUCUAGAGGGAAGCACUUUCUC | 27.14 | ± 1.54 | 31.92 | ± 2.40 |
| **hsa-miR-519a*** | CUCUAGAGGGAAGCGCUUUCUG | 32.39 | ± 0.55 | 32.48 | ± 1.44 |
| **hsa-miR-519e*** | UUCUCCAAAAGGGAGCACUUUC | 26.55 | ± 1.01 | 30.97 | ± 0.10 |
| **hsa-miR-522*** | CUCUAGAGGGAAGCGCUUUCUG | 33.93 | ± 6.59 | 29.57 | ± 3.51 |
| **hsa-miR-523*** | CUCUAGAGGGAAGCGCUUUCUG | 33.37 | ± 4.37 | 31.20 | ± 1.67 |
| **hsa-miR-526b*** | GAAAGUGCUUCCUUUUAGAGGC | 27.90 | ± 1.29 | 29.56 | ± 4.22 |
| **hsa-miR-541*** | AAAGGAUUCUGCUGUCGGUCCCACU | 25.13 | ± 2.22 | 28.00 | ± 1.50 |
| **hsa-miR-545*** | UCAGUAAAUGUUUAUUAGAUGA | 28.91 | ± 1.55 | 36.47 | ± 1.55 |
| **hsa-miR-550*** | UGUCUUACUCCCUCAGGCACAU | 27.05 | ± 1.67 | 32.65 | ± 1.55 |
| **hsa-miR-551b*** | GAAAUCAAGCGUGGGUGAGACC | 26.00 | ± 0.42 | 27.88 | ± 4.27 |
| **hsa-miR-589*** | UCAGAACAAAUGCCGGUUCCCAGA | 26.36 | ± 3.32 | 28.23 | ± 1.01 |
| **hsa-miR-593*** | AGGCACCAGCCAGGCAUUGCUCAGC | 25.73 | ± 1.09 | 27.62 | ± 3.74 |
| **hsa-miR-616*** | ACUCAAAACCCUUCAGUGACUU | 24.01 | ± 1.55 | 20.47 | ± 1.46 |
| **hsa-miR-624*** | UAGUACCAGUACCUUGUGUUCA | 27.72 | ± 2.26 | 28.42 | ± 6.20 |
| **hsa-miR-625*** | GACUAUAGAACUUUCCCCCUCA | 23.19 | ± 2.69 | 24.72 | ± 2.17 |
| **hsa-miR-629*** | GUUCUCCCAACGUAAGCCCAGC | 28.66 | ± 1.91 | 30.64 | ± 1.75 |
| **hsa-miR-664*** | ACUGGCUAGGGAAAAUGAUUGGAU | 28.01 | ± 1.37 | 23.11 | ± 2.06 |
| **hsa-miR-675*** | CUGUAUGCCCUCACCGCUCA | 24.99 | ± 2.30 | 27.25 | ± 2.92 |
| **hsa-miR-708*** | CAACUAGACUGUGAGCUUCUAG | 29.03 | ± 1.47 | 31.87 | ± 0.99 |
| **hsa-miR-744*** | CUGUUGCCACUAACCUCAACCU | 26.68 | ± 2.52 | 29.44 | ± 2.22 |
| **hsa-miR-877*** | UCCUCUUCUCCCUCCUCCCAG | 26.89 | ± 0.82 | 28.46 | ± 7.01 |
| **hsa-miR-888*** | GACUGACACCUCUUUGGGUGAA | 28.80 | ± 1.73 | 31.61 | ± 0.75 |
| **hsa-miR-1226*** | GUGAGGGCAUGCAGGCCUGGAUGGGG | 26.78 | ± 1.43 | 28.08 | ± 2.26 |
| **hsa-miR-1228*** | GUGGGCGGGGGCAGGUGUGUG | 27.00 | ± 1.96 | 28.79 | ± 7.49 |
| **hsa-miR-1909*** | UGAGUGCCGGUGCCUGCCCUG | 26.15 | ± 2.54 | 27.87 | ± 0.40 |
| **hsa-miR-1911*** | CACCAGGCAUUGUGGUCUCC | 27.52 | ± 2.07 | 29.07 | ± 6.84 |
| **hsa-miR-1914*** | GGAGGGGUCCCGCACUGGGAGG | 27.77 | ± 1.75 | 33.34 | ± 0.98 |
| **hsa-miR-1915*** | ACCUUGCCUUGCUGCCCGGGCC | 16.41 | ± 3.00 | 18.84 | ± 3.51 |
| **hsa-miR-2114*** | CGAGCCUCAAGCAAGGGACUU | 28.01 | ± 0.66 | 32.79 | ± 4.13 |
| **hsa-miR-2115*** | CAUCAGAAUUCAUGGAGGCUAG | 29.62 | ± 2.45 | 32.17 | ± 0.66 |
| **hsa-miR-2116*** | CCUCCCAUGCCAAGAACUCCC | 27.87 | ± 3.01 | 29.62 | ± 0.22 |
| **hsa-miR-449c*** | UUGCUAGUUGCACUCCUCUCUGU | 25.54 | ± 2.44 | 22.90 | ± 1.46 |

**Table S4. Healthy e-sEV and i-sEV miRNOme analysis of 1140 miRNAs.** RT-PCR of 1140 miRNA using miRNome miRNA Profilers QuantiMir. Results are listed as Ct average±SD from the different samples (n= 3) of both EV population: effective sEV (n= 3) and ineffective sEV (n=3) collected from serum of healthy subjects.

|  | ***Average Ct*** | | | | | | | | | | | | | | | |
| --- | --- | --- | --- | --- | --- | --- | --- | --- | --- | --- | --- | --- | --- | --- | --- | --- |
|  | **miR-126** | | **miR-21** | | **miR-296-3p** | | **miR-210** | | **miR-130a** | | **miR-27a** | | **miR-29a** | | **miR-191** | |
| ***1 H*** | 28.93 | ±0.98 | 29.92 | ±0.22 | 32.68 | ±0.57 | 33.65 | ±0.88 | 28.01 | ±0.85 | 29.73 | ±0.42 | 31.47 | ±0.57 | 29.59 | ±0.44 |
| ***2 H*** | 29.48 | ±0.75 | 32.72 | ±0.75 | 29.48 | ±0.44 | 30.34 | ±0.78 | 30.95 | ±0.91 | 29.48 | ±0.14 | 32.72 | ±0.55 | 29.48 | ±0.56 |
| ***3 H*** | 26.37 | ±0.54 | 28.69 | ±0.88 | 31.66 | ±0.52 | 33.79 | ±0.55 | 28.97 | ±0.76 | 28.85 | ±0.55 | 30.16 | ±0.55 | 27.93 | ±0.14 |
| ***4 H*** | 27.30 | ±0.22 | 29.34 | ±0.89 | 32.29 | ±0.65 | 33.85 | ±0.32 | 28.81 | ±0.82 | 29.24 | ±0.51 | 30.63 | ±0.96 | 28.63 | ±0.65 |
| ***5 H*** | 28.03 | ±0.44 | 29.02 | ±0.95 | 31.68 | ±0.88 | 32.84 | ±0.91 | 29.25 | ±0.93 | 29.83 | ±0.86 | 31.37 | ±0.75 | 29.49 | ±0.75 |
| ***6 H*** | 26.67 | ±0.54 | 28.09 | ±0.99 | 31.06 | ±0.47 | 30.19 | ±0.84 | 30.57 | ±0.86 | 28.35 | ±0.87 | 30.86 | ±0.65 | 27.53 | ±0.69 |
| ***7 H*** | 28.00 | ±0.88 | 29.74 | ±0.45 | 32.89 | ±0.58 | 30.34 | ±0.64 | 28.95 | ±0.55 | 29.84 | ±0.56 | 30.43 | ±0.36 | 28.73 | ±0.64 |
| ***8 H*** | 26.37 | ±0.96 | 28.69 | ±0.66 | 31.66 | ±0.63 | 32.79 | ±0.78 | 29.97 | ±0.43 | 28.85 | ±0.41 | 30.16 | ±0.47 | 27.93 | ±0.75 |
| ***9 H*** | 27.30 | ±1.10 | 29.34 | ±0.63 | 32.29 | ±0.25 | 33.45 | ±1.10 | 28.31 | ±0.56 | 29.24 | ±0.53 | 30.63 | ±0.58 | 28.63 | ±0.57 |
| ***1 D*** | 27.56 | ±1.23 | 30.02 | ±0.62 | 32.01 | ±0.45 | 30.17 | ±0.25 | 34.00 | ±0.21 | 28.54 | ±0.43 | 29.48 | ±0.23 | 29.53 | ±0.35 |
| ***2 D*** | 28.16 | ±1.10 | 29.56 | ±0.36 | 33.45 | ±0.65 | 29.39 | ±0.33 | 33.52 | ±0.35 | 29.11 | ±0.96 | 31.93 | ±0.21 | 28.53 | ±1.23 |
| ***4 D*** | 33.48 | ±0.78 | 34.46 | ±0.52 | 35.69 | ±0.77 | 29.29 | ±0.45 | 34.88 | ±0.46 | 36.15 | ±0.45 | 35.75 | ±0.31 | 34.98 | ±0.25 |
| ***5 D*** | 31.80 | ±0.69 | 32.41 | ±0.53 | 36.03 | ±0.72 | 29.48 | ±0.57 | 32.63 | ±0.53 | 35.31 | ±0.78 | 34.51 | ±0.34 | 32.79 | ±0.65 |
| ***7 D*** | 27.86 | ±0.77 | 30.32 | ±0.78 | 32.21 | ±0.71 | 30.07 | ±0.67 | 33.80 | ±0.64 | 28.84 | ±0.42 | 29.78 | ±0.46 | 29.43 | ±0.77 |
| ***8 D*** | 28.16 | ±1.09 | 29.66 | ±0.36 | 33.75 | ±0.38 | 29.58 | ±1.02 | 29.73 | ±0.33 | 29.11 | ±0.41 | 31.73 | ±0.48 | 28.73 | ±0.88 |
| ***17 D*** | 27.96 | ±1.02 | 30.22 | ±1.22 | 32.41 | ±1.23 | 30.07 | ±0.33 | 29.90 | ±1.81 | 28.94 | ±0.62 | 29.98 | ±0.52 | 29.63 | ±0.52 |
| ***18 D*** | 28.06 | ±0.56 | 29.76 | ±0.74 | 33.85 | ±0.78 | 31.49 | ±0.77 | 29.42 | ±0.76 | 29.21 | ±0.58 | 31.83 | ±0.83 | 28.83 | ±0.84 |
| ***20 D*** | 33.38 | ±1.07 | 34.56 | ±0.65 | 35.59 | ±0.02 | 31.97 | ±1.01 | 28.98 | ±0.88 | 36.05 | ±0.68 | 35.85 | ±0.73 | 34.88 | ±0.58 |
| ***15 O*** | 26.77 | ±0.99 | 28.38 | ±0.74 | 34.20 | ±0.09 | 29.63 | ±0.52 | 32.90 | ±0.58 | 30.22 | ±0.47 | 31.43 | ±0.91 | 28.05 | ±0.11 |
| ***33 O*** | 28.94 | ±0.55 | 31.69 | ±0.55 | 32.98 | ±0.12 | 29.46 | ±0.45 | 34.24 | ±0.41 | 29.83 | ±0.75 | 30.57 | ±0.95 | 30.38 | ±0.65 |
| ***22 O*** | 31.38 | ±0.99 | 31.72 | ±0.65 | 36.50 | ±0.56 | 29.33 | ±0.99 | 32.50 | ±0.91 | 33.55 | ±0.56 | 35.46 | ±0.87 | 31.87 | ±0.25 |
| ***31 O*** | 28.44 | ±1.06 | 31.29 | ±1.22 | 32.38 | ±1.00 | 28.46 | ±1.00 | 35.24 | ±1.00 | 29.33 | ±1.14 | 30.07 | ±0.25 | 30.78 | ±0.36 |
| ***32 O*** | 30.38 | ±0.97 | 30.72 | ±0.99 | 35.50 | ±0.96 | 28.63 | ±0.87 | 33.90 | ±0.88 | 32.55 | ±0.78 | 34.46 | ±0.45 | 32.87 | ±0.47 |
| ***12 O*** | 27.94 | ±0.28 | 30.69 | ±1.23 | 33.98 | ±0.35 | 30.97 | ±0.22 | 30.98 | ±1.01 | 28.83 | ±0.23 | 31.57 | ±0.88 | 29.38 | ±0.91 |
| ***34 O*** | 25.77 | ±0.33 | 29.38 | ±0.85 | 33.20 | ±0.77 | 28.46 | ±0.25 | 34.24 | ±0.20 | 29.22 | ±0.78 | 32.43 | ±0.65 | 27.05 | ±0.47 |
| ***35 O*** | 30.38 | ±0.78 | 30.72 | ±0.91 | 37.50 | ±0.97 | 29.58 | ±0.13 | 32.73 | ±0.10 | 31.55 | ±0.53 | 36.46 | ±0.42 | 30.87 | ±0.38 |
| ***19 O*** | 27.96 | ±0.96 | 30.22 | ±1.01 | 32.41 | ±0.33 | 31.97 | ±0.96 | 31.98 | ±1.53 | 28.94 | ±0.88 | 29.98 | ±0.56 | 29.63 | ±0.37 |
| ***13 OD*** | 28.61 | ±0.56 | 30.48 | ±0.03 | 32.85 | ±0.45 | 29.15 | ±0.65 | 31.61 | ±0.68 | 28.71 | ±1.23 | 30.18 | ±0.25 | 29.32 | ±0.71 |
| ***3 OD*** | 28.61 | ±0.78 | 30.18 | ±0.14 | 32.75 | ±0.58 | 29.45 | ±0.87 | 33.31 | ±0.56 | 28.71 | ±0.56 | 30.28 | ±0.69 | 29.22 | ±0.56 |
| ***6 OD*** | 28.31 | ±0.82 | 30.48 | ±0.75 | 32.85 | ±0.78 | 29.45 | ±0.86 | 33.51 | ±0.87 | 28.61 | ±0.45 | 30.28 | ±0.42 | 29.22 | ±0.14 |
| ***21 OD*** | 29.83 | ±0.47 | 31.58 | ±1.02 | 33.45 | ±0.36 | 29.56 | ±0.32 | 32.90 | ±1.00 | 30.28 | ±0.53 | 31.26 | ±0.47 | 30.30 | ±0.57 |
| ***9 OD*** | 28.12 | ±0.83 | 29.91 | ±0.85 | 37.11 | ±0.75 | 32.86 | ±0.73 | 29.79 | ±0.75 | 30.67 | ±0.75 | 32.05 | ±0.36 | 28.98 | ±0.58 |
| ***10 OD*** | 30.19 | ±1.05 | 32.48 | ±0.35 | 32.67 | ±0.44 | 30.88 | ±1.00 | 30.86 | ±0.22 | 33.21 | ±0.42 | 34.94 | ±0.25 | 30.94 | ±0.85 |
| ***14 OD*** | 29.73 | ±0.77 | 31.78 | ±0.44 | 33.35 | ±0.85 | 29.76 | ±0.65 | 29.00 | ±0.26 | 30.18 | ±0.36 | 31.16 | ±0.14 | 30.20 | ±0.95 |
| ***36 OD*** | 28.41 | ±0.52 | 30.58 | ±0.46 | 32.95 | ±0.78 | 29.35 | ±0.33 | 29.41 | ±0.31 | 28.81 | ±0.85 | 30.18 | ±0.58 | 29.12 | ±0.64 |
| ***16 OD*** | 28.41 | ±0.88 | 30.08 | ±0.43 | 32.45 | ±0.15 | 29.45 | ±0.75 | 29.63 | ±0.72 | 28.61 | ±0.65 | 30.18 | ±0.36 | 29.42 | ±0.34 |
| ***28 IC*** | 31.97 | ±1.07 | 31.98 | ±1.03 | 31.97 | ±0.53 | 29.63 | ±1.00 | 32.90 | ±1.03 | 31.97 | ±0.55 | 31.98 | ±0.14 | 31.97 | ±0.46 |
| ***24 IC*** | 29.63 | ±0.88 | 32.90 | ±0.55 | 29.63 | ±0.86 | 29.46 | ±0.53 | 34.24 | ±0.50 | 29.63 | ±0.22 | 29.11 | ±0.55 | 29.63 | ±0.19 |
| ***11 IC*** | 29.46 | ±0.44 | 34.24 | ±0.45 | 29.46 | ±0.47 | 29.33 | ±0.44 | 32.50 | ±0.41 | 29.46 | ±0.66 | 28.94 | ±0.89 | 29.46 | ±0.18 |
| ***26 IC*** | 29.33 | ±0.48 | 32.50 | ±0.44 | 33.45 | ±0.25 | 28.46 | ±0.48 | 35.24 | ±0.49 | 29.33 | ±0.42 | 29.21 | ±0.24 | 29.33 | ±0.23 |
| ***27 IC*** | 28.31 | ±0.47 | 35.24 | ±0.67 | 37.11 | ±0.63 | 28.63 | ±0.68 | 33.90 | ±0.67 | 28.46 | ±0.53 | 36.05 | ±0.47 | 28.46 | ±0.34 |
| ***23 IC*** | 29.83 | ±0.99 | 29.46 | ±0.65 | 32.67 | ±0.78 | 30.97 | ±0.83 | 30.98 | ±0.88 | 28.63 | ±0.87 | 30.22 | ±0.58 | 29.43 | ±0.28 |
| ***29 IC*** | 28.12 | ±0.92 | 29.33 | ±0.47 | 33.35 | ±0.47 | 28.46 | ±0.91 | 34.24 | ±0.90 | 30.97 | ±0.36 | 30.98 | ±1.36 | 28.73 | ±1.05 |
| ***25 IC*** | 30.19 | ±1.08 | 28.46 | ±1.04 | 28.46 | ±0.23 | 29.58 | ±1.00 | 32.73 | ±1.04 | 28.46 | ±0.33 | 34.24 | ±0.99 | 29.63 | ±0.82 |
| ***30 IC*** | 29.73 | ±0.82 | 28.63 | ±0.25 | 29.58 | ±0.27 | 29.48 | ±0.81 | 32.72 | ±0.82 | 29.58 | ±1.03 | 32.73 | ±0.45 | 28.83 | ±0.66 |

**Table S5.** **Selected sEV** **miR-distribution.** Average Ct values of miR-126, miR-21, miR-296-3p, miR-210, miR-130a, miR-27a, miR-29a, miR-191. H= Healthy subjects; D = Diabetic patients; O= Obese patients; OD= Obese/Diabetic patients; IC = Ischemic patients.
